# Topical wound-care products and their effects on healing, inflammatory biomarkers, and growth in piglets undergoing castration

**DOI:** 10.1186/s40813-026-00492-7

**Published:** 2026-04-21

**Authors:** Laya Kannan Silva Alves, Monique Danielle Pairis-Garcia, Juliana Bonin Ferreira, Victoria Rocha Merenda, Rubia Mitalli Tomacheuski, Pedro Henrique Esteves Trindade, Christopher Siepker, Magdiel Lopez-Soriano

**Affiliations:** 1 Department of Animal Nutrition and Production, School of Veterinary Medicine and Animal Science, University of Sao Paulo, Pirassununga, SP Brazil; 2 Department of Population Health and Pathobiology, College of Veterinary Medicine, North Carolina State University, Raleigh, NC USA; 3 Department of Veterinary Clinical Sciences, College of Veterinary Medicine, Washington State University, Pullman, WA USA; 4 Department of Large Animal Clinical Sciences, College of Veterinary Medicine, Michigan State University, East Lansing, MI USA; 5 Vet Diagnostic & Production Animal Medicine, College of Veterinary Medicine, Iowa State University, Ames, IA USA; 6 Extension Department, University of Missouri, Columbia, MO USA

**Keywords:** Acute phase proteins, Animal welfare, Piglet mortality, Thermography, Weaning, Wound healing

## Abstract

Surgical castration is a routine management procedure in swine production that raises welfare concerns due to pain, inflammation, and risk of post-procedure complications. Topical products are commonly applied to castration wounds, but their efficacy in promoting healing and reducing inflammation has not been systematically evaluated. This study investigated and compared the efficacy of five commercially available antiseptic and barrier topical products on wound healing, inflammatory responses, and growth performance in piglets undergoing surgical castration. One hundred and ninety piglets, 3–5 days-old of age, were evaluated under the following treatments: Iodine, Oinkment^®^, PhytoCare^®^, Vetericyn^®^, Zinc Oxide, or intact controls (NoCast). Treatments were applied immediately after castration (D1). Body weights were recorded at baseline (D0; day before castration) and at weaning. Blood samples were collected on days 0 (baseline), 7, and 14 for analysis of prostaglandin E₂ (PGE₂) and haptoglobin. Infrared thermography (IRT) was used to assess surface temperature. Histological evaluation of wound healing was performed on subsets of piglets on days 7 and 14. No treatment effects were observed on body weight or pre-weaning survival; castrated piglets grew similarly to intact controls. Concentrations of PGE₂ declined over time (*P* < 0.001) but did not differ between treatments, suggesting it may have limited utility as an inflammatory biomarker in neonatal pigs. Haptoglobin concentrations increased across all groups by days 7 and 14, including intact controls, indicating minimal specificity for castration-related inflammation. In contrast, IRT consistently distinguished castrated from intact piglets, supporting its potential as a non-invasive indicator of inflammatory responses. Histological evaluations showed expected time-dependent healing progression, with epidermal thickness correlating with wound severity, but no treatment effects were found. None of the tested topical products were superior to others in regard to wound healing or reduced systemic inflammation under a single-application protocol. While safe and without adverse effects on growth, benefits are unclear when compared to a traditional iodine treatment. The current standard operational procedure for castration requires piglets to receive Iodine after castration to reduce infection risk. Future research should explore no treatment option, repeated applications, microbial wound presence, and behavioral indicators to better evaluate post-castration wound-care strategies.

**Supplementary Information:**

The online version contains supplementary material available at 10.1186/s40813-026-00492-7.

## Introduction

Castration is a prevalent procedure conducted on swine farms in the United States to avert undesirable breeding, diminish aggression, and enhance pork quality [1, 2]. This technique is painful and has been recognized as a major welfare issue within the US swine industry [3, 4]; as well from the global public’s perspective [5, 6]. Surgical castration is typically conducted within the first 7 days of the piglet’s life, utilizing an open technique where two incisions are made in the scrotal skin to expose and remove the testicles [7]. After the testicles are removed, the spermatic cords are severed, and the incisions are left open to heal naturally without sutures [8, 9].

Surgical castration induces marked physiological and behavioral changes in piglets. Acute stress response following castration results in increased plasma cortisol concentrations [10], and tissue-level inflammation indicated by acute phase protein secretion [11]. From a behavioral standpoint, marked deviations in maintenance behaviors and increase performance of pain-associated behaviors can be consistently observed post-procedure [12, 13]. Piglets experience negative affective states of pain and distress, which can have both acute and long-term implications from a welfare standpoint. Although chronic effects of pain and healing in castrated pigs are lacking [14], long-term consequences to pig welfare have been noted in tail docked pigs [15], resulting in hypersensitivity, chronic pain and poor wound healing [16].

In addition to the negative welfare impact to the individual pig, castration also increases the risk of morbidity and mortality during the pre-weaning phase [17–19], with potential complications including excessive bleeding, infection, delayed wound healing, and herniation at the incision site [19–21]. These risks are particularly elevated in lighter pigs or those with comorbidities [18, 22].

In addition to the physiological complications and increased risk of morbidity, these outcomes contribute to an overall negative welfare state for the piglet. According to the Five Domains model [23], such impacts can be systematically interpreted across multiple dimensions of welfare, including health, environment, and nutrition, with each influencing the piglet’s affective state. This framework provides a structure method to evaluate how physical and functional disruptions, such as tissue injury and impaired growth, ultimately shape the mental experiences of the animal. In the context of castration, the procedure directly compromises the health domain (through pain, inflammation, and infection risk), the environment domain (via exposure of open wounds to pathogens), and the nutrition and performance domain (through potential reductions in growth due to morbidity).

Despite the welfare and economic implications of castration, limited research has been conducted evaluating effective and practical strategies for improving post-castration wound healing and decreasing pre-weaning mortality and morbidity. To date, the most common post-castration protocol utilized on-farm is the use of a topical iodine spray applied to the incision site. Iodophore-based formulations such as iodine demonstrate a variety of favorable characteristics including its ability to penetrate biofilms, broad antimicrobial spectrum, low cytotoxicity, good tolerability and known anti-inflammatory properties [24]. Iodine is commonly used in both human and animal settings, with pig producers utilizing iodine frequently following castration to reduce bacterial load and minimize environmental contamination [25–27]. However, no studies to date have evaluated this method nor compared it to alternative wound care products and their effects on the physiological response of the piglet during the wound healing process.

To reduce piglet loss associated with castration-related death, identifying an effective product that improves wound healing rates and decreases infection associated with castration is critical. Therefore, the objective of this study was to compare the efficacy of commercially available topical protective products on wound healing, inflammatory responses, and growth performance in piglets undergoing surgical castration.

## Materials and methods

### Housing and animals

This study was approved by the Institutional Animal Care and Use Committee of North Carolina State University (IACUC protocol 20–113).

The experiment was conducted on a commercial sow farm in the southeastern United States during the summer. Sows and piglets were housed in individual farrowing crates within tunnel-ventilated, fully slatted farrowing rooms maintained at an average temperature of 22 °C ± 1.0 °C. Temperature and ventilation were controlled using a computerized system. Each farrowing crate measured 2.5 m × 0.7 m, with an additional piglet area (2.5 m × 1.3 m). Heat mats were provided for piglets and maintained at approximately 30–35 °C. Lighting was provided from 0600 to 1700 h. Feed and water were offered *ad libitum* to both sows and piglets.

Animal care and handling followed the Guide for the Care and Use of Agricultural Animals in Research and Teaching [28]. Surgical castration was a routine farm practice; therefore, male piglets enrolled in this study were not castrated solely for research purposes.

### Sample size

A priori power analysis was conducted to determine the required sample size to detect differences in inflammatory biomarkers across treatments. Using published mean differences in PGE₂ between castrated and non-castrated piglets [29] and assuming a moderate effect size (f = 0.25), a one-way ANOVA power analysis (α = 0.05, power = 0.80, k = 6 groups) indicated that approximately 35 piglets per treatment group were required. The study concluded with 31–32 piglets per group with complete data due to subsequent morbidity and mortality.

### Inclusion criteria

Piglets were enrolled in the study according to the following criteria: 3–5 days of age, body live weight > 0.5 kg, both testicles fully descended, full and intact tail, no visible clinical signs of disease.

### Experimental design and treatment administration

**Fig. 1:**
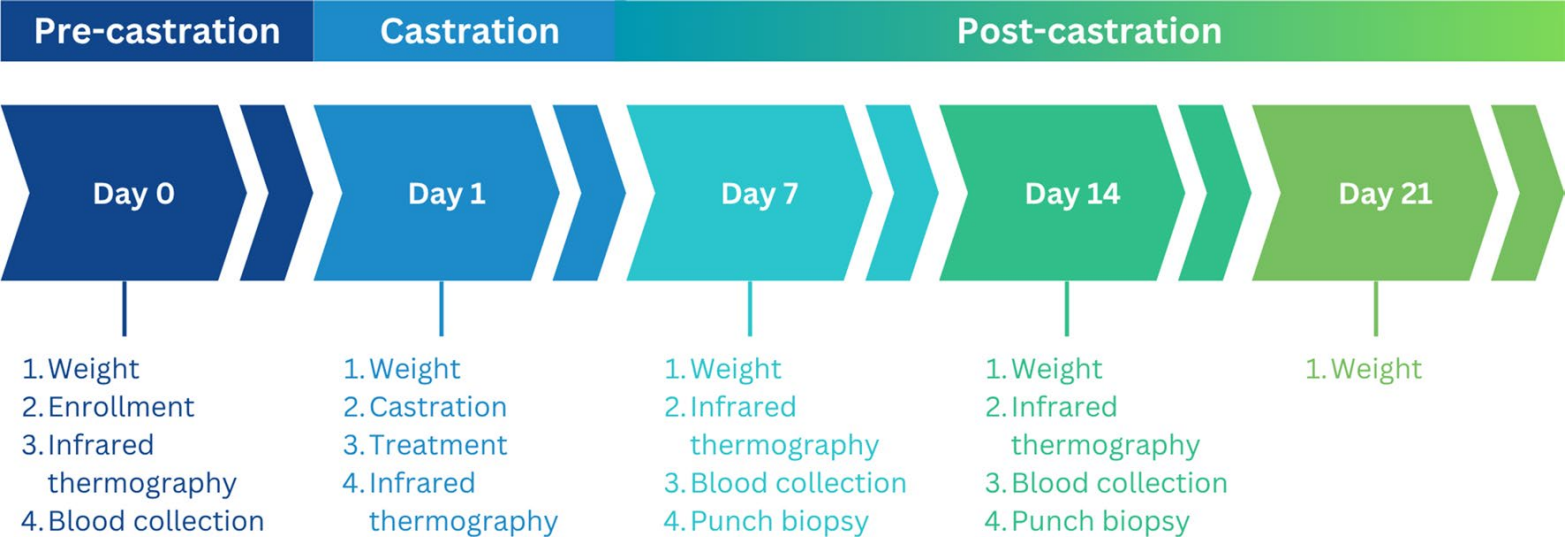
Overview of the experimental timeline and data collection order

A total of 190 Large White x Duroc male piglets from 51 litters were evaluated in this study (study duration = 21 days; average age: 3–5 days; Fig. 1).

At enrollment (D0), piglets were individually identified using ear tags (Allflex Global Piglet ear tags, Allflex Livestock Intelligence, Madison, WI), blocked by litter and body weight, and randomly assigned to one of six treatment groups:

- I (Iodine; AgriLabs, St Joseph, MO, USA): male piglets (*n* = 32) surgically castrated with topical application of iodine at the incision site immediately post-procedure.
- NoCast (Non-castrated): Intact male piglets (*n* = 32) subjected to sham castration with topical application of iodine to the testicles post-sham procedure.
- O (Oinkment^®^; Animal Science Products; Nacogdoches, TX, USA): Male piglets (*n* = 32) surgically castrated with topical application of Oinkment^®^ at the incision site post-procedure.
- PC (PhytoCare Swine Skin Recovery & Care^®^; Precision Health Technologies, Brookings, SD, USA): Male piglets (*n* = 31) surgically castrated with topical application of PhytoCare Swine Skin Recovery & Care^®^ at the incision site post-procedure.
- VP (Vetericyn Plus^®^; Vetericyn, Rialto, CA, USA): Male piglets (*n* = 31) surgically castrated with topical application of Vetericyn Plus^®^ at the incision site post-procedure.
- ZO (Zinc Oxide Ointment USP^®^; Rugby Laboratories, Livonia, MI, USA): Male piglets (*n* = 32) surgically castrated with topical application of Zinc Oxide Ointment USP^®^ at the incision site post-procedure.

All treatments were applied immediately post-castration (D1) according to the manufacturer’s recommendations. Liquid treatments (iodine, Phytocare, Vetericyn) were administered using a spray bottle (1–3 mL per piglet) directly on the incision site, while paste products (Zinc Oxide Ointment and Oinkment) were applied via a gloved hand (2–3 g per piglet) directly on the incision site. Gloves were changed and hands washed in between treatment groups. A summary of the active ingredients and intended functions of each topical product is provided in Table 1.

**Table 1:** Active ingredients and intended functions of the topical products evaluated

Wound-care product	Active ingredient	Mechanisms of wound-healing action	Product’s information	
Iodine Wound Spray^®^	Alpha (p-Nonylphenyl) Omega-Hydroxypoly (oxyethylene); Iodine complex (5%; ≥1% titratable iodine); Water and nonionic surfactant (95%).	Broad-spectrum antimicrobial activity; reduces bacterial load at surgical sites.	10.6084/m9.figshare.31211551 (Additional file 1)	
Oinkment^®^	Ethyl alcohol (72.6%); sodium propionate (0.5%).	Rapid antiseptic effect; antifungal and antimicrobial activity.	10.6084/m9.figshare.31211563 (Additional file 2)	
PhytoCare Swine Skin Recovery & Care^®^	Botanical extracts (Punica granatum, Camellia sinensis, Vitis vinifera) rich in polyphenols.	Antioxidant properties; supports tissue recovery via reactive plant immunity pathways.	10.6084/m9.figshare.31211557 (Additional file 3)	
Vetericyn Plus^®^	Hypochlorous acid (0.012%); electrolyzed water solution (NaCl, phosphates, NaOCl)	Non-cytotoxic antimicrobial effect; wound cleansing without tissue damage.	10.6084/m9.figshare.31211554 (Additional file 4)	
Zinc Oxide Ointment USP^®^	Zinc oxide (20%); inactive barrier / emollient components (cetomacrogol 1000, cetostearyl alcohol, mineral oil, white soft paraffin).	Skin barrier formation; moisture protection; supports epithelial repair.	10.6084/m9.figshare.31211560 (Additional file 5)	

Iodine (I) - AgriLabs, St. Joseph, MO, USA; Oinkment^®^ (O) - Animal Science Products; Nacogdoches, TX, USA; PhytoCare Swine Skin Recovery & Care^®^ (PC) - Precision Health Technologies, Brookings, SD, USA; Vetericyn Plus^®^ (VP) - Vetericyn, Rialto, CA, USA; and Zinc Oxide Ointment USP^®^ (ZO) - Rugby Laboratories, Livonia, MI, USA

A castrated control group with no topical treatment could not be included as a treatment group as it deviated the current standard operating procedure (SOP) for the commercial swine operation and the approved operating protocol required any pig undergoing castration to receive a topical application of iodine at the wound site.

### Castration procedure

Castration was performed within the farrowing room on D1 by a trained researcher (MLS) with over 10 years of swine production experience. Piglets were restrained by holding both hind legs with the head down. A sterile scalpel blade was used to make two, one cm vertical incisions through the scrotal skin over each testicle. The same scalpel blade was used to complete castration within the litter, and a new sterile scalpel blade was used for subsequent litter. Following incision, the testicles were exteriorized, the spermatic cords were torn, and additional spermatic cord tissues removed by traction if needed. Piglets in the non-castrated group underwent a sham procedure in which they were restrained similarly, but only external pressure was applied to the scrotal area without making an incision. The same researcher performed both castration and sham procedures to ensure consistency. Average procedure duration was less than one minute, and behavioral indicators of hypothermia were not observed during the procedure.

### Data collection

All data was collected within the farrowing room near the farrowing crate from which the piglet and its litter was located. Data collection order can be found in Fig. 1.

#### Piglet performance

Piglet body weight and age were recorded at enrollment (D0; day prior to castration), on castration day (D1), and subsequently on days 7, 14, and 21 (weaning). Average body weight (BW) and average daily gain (ADG) were calculated for each treatment at all time points based on live pig weight. Pre-weaning mortality was also assessed throughout the 21-day nursing period.

#### Physiological response

Blood samples were collected to assess prostaglandin E_2_ (PGE_2)_ and haptoglobin concentration for each piglet (n = 190) 24 h prior to castration (D0 - baseline), 7-day post-castration, and 14 days post-castration (Fig. 1). Blood collection was performed using a technique previously described by Dove and Alworth [30]; Merenda et al. [29, 31] and Lopez-Soriano et al. [32]. Samples were obtained via orbital sinus puncture using a 20-22G x 1” Exel^®^ disposable hypodermic needle (Exel International, Quebec, Canada) and collected into 4mL BD^®^ red-top vacutainer serum tubes (Med Vet International, Mettawa, IL).

During the blood collection procedure, one technician restrained the piglet by holding both the front and back legs while positioning the piglet’s back against her chest in a dorsal recumbent position. The phlebotomist knelt close to the piglet and used his non-dominant hand to wrap around the snout, placing his thumb over the upper jaw without restricting the animal’s breathing. The phlebotomist used their dominant hand to insert the needle at a 90° angle into the nictitating membrane (2.0–2.5 cm) targeting the lacrimal caruncle. In order for blood to flow, the phlebotomist held the vacutainer serum tube in their dominant hand and with the thumb removed the lid from the tube and collected blood. If blood was not flowing, the phlebotomist would rotate the needle in a slow motion and withdraw and reinserted the needle a short distance to encourage blood to flow. A total of 3–4 mL of blood was obtained within 5 to 8 s. Afterwards, the phlebotomist carefully withdrew the needle and applied pressure to the puncture site with his thumb for 5 s to cause clotting. Piglets demonstrated minor distress throughout the procedure and resumed normal behaviors within the first 3 min of post-completion.

The tubes were immediately placed in a cooler and centrifuged (2,000 × g for 15 min at 4 °C) within eight hours of collection to separate serum. The serum was then aliquoted into 1.5mL Axygen^®^ microcentrifuge tubes (Axygen Scientific, Corning, NY) at − 80 °C.

The assays for the biomarker analysis were completed six to nine months post-collection. PGE_2_ concentrations were measured using a commercial enzyme-linked immunosorbent assay (ELISA; catalog No. 514531; Cayman Chemical) following the method described by Giorgi et al. [33]. Briefly, serum samples were purified by adding ice-cold acetone (4x the serum volume), followed by incubation at − 20° C for 30 min and centrifugation (3,000 x g for 5 min). The supernatant was transferred to a 13 × 100-mm glass tube, evaporated using a CentriVap concentrator (Labconco), and reconstituted to the original serum volume with kit buffer. An aliquot of the reconstituted sample was derivatized with adjusted kit components and the manufacturer’s protocol were followed. Samples were analyzed in duplicate, and absorbance was measured at 405 nm following 60 min of development (SpectraMax i3; Molecular Devices). The mean PGE_2_ concentration of a reference sample used for repeatability assessment was 12.42 pg/mL (range: 9.90–15.60 pg/mL), with an inter-assay coefficient of variation of 8.61% [31].

Haptoglobin concentrations were determined using the Tridelta Phase^®^ Haptoglobin Assay (catalog No. TP-801; Tridelta Development Ltd., Maynooth, Ireland). The assay utilized Haemoglobin (reagent 1), Chromogen (reagent 2), calibrator/sample diluent, and calibrator (microplate method). A curve was generated for each assay using haptoglobin standards at 2.5, 1.25, 0.625, 0.312, and 0 mg/mL. The assay procedure involved dispensing reagents 1 and 2 into storage vessels within the instrument, followed by aliquoting samples, controls, and calibrators into the appropriate sample cuts. Absorbance was then measured at 600–630 nm while the calibration curve developed. Results were interpreted by calculating the mean absorbance for each sample, control, or standard. A calibration curve was generated by plotting absorbance (600–630 nm) against haptoglobin concentration (mg/mL), and a smooth curve was fitted through the data points. For porcine samples, expected haptoglobin values were categorized as follows: normal range (0.00-2.2- mg/mL) and acute phase range (3.00–8.00 mg/mL). The intra assay and inter assay coefficients of variation were 5.8% and 4.9%, respectively.

#### Inflammatory response

Infrared thermography (IRT) was used to detect changes in skin temperature associated with inflammation. Image collection followed the methodology described by Bates et al. [34], utilizing a portable infrared camera (Degree2Act). Temperature changes were analyzed by comparing the piglets’ skin temperature at different time points: 24 h before castration (D0 - baseline), immediately after castration (D1), and at 7- and 14-day post-castration.

#### Histopathological response

To assess histopathological changes at the incision site, punch biopsies were collected on days 7 and 14 of the trial from five pigs per treatment group. The randomly selected piglets received pain control consisting of 1 mL of 2% lidocaine applied in the inguinal area and flunixin meglumine (2.2 mg/kg) administered intranasally 20 min prior to sample collection.

Punch biopsies were obtained using a 5.0 mm round-tipped cutting tool (Scientific Labwares Disposable Punch Biopsy^®^, Gainesville, VA, USA) from the scrotal skin at the castration incision site. The samples were collected by applying slight downward pressure and rotating the device clockwise. Once the skin was punctured, the punch device was carefully removed, and the skin samples were grasped with forceps. The underlying fat was then separated using scissors. Samples were immediately fixed in 10% formalin [35]. Histological samples were processed using Masson’s trichrome staining, and a board-certified pathologist evaluated them based on the variables described in Table 2.

**Table 2:** Wound healing objective criteria (adapted from Santos et al. [36] and Van de Vyver et al. [37])

Score:	0	1	2	3
Epidermal ulceration	intact (< 5%)	partial (5–25%)	ulceration (25–50%)	complete ulceration (> 50%)
Epidermal thickness index^1^	normal (95–105%)	mild (< 110%)	moderate (110–120%)	marked (> 120%)
Serocellular crusting	absent	mild (25–50%)	moderate (50–75%)	marked (> 75%)
Inflammatory infiltrate	minimal	mild	moderate	marked
Granulation tissue	none/minimal	mild (10–25%)	moderate (25–50%)	proliferative (> 50%)
Dermal hemorrhage	absent	mild	moderate	marked

^1^ETI = ([average thickness of epidermis in wound area]/[average thickness of epidermis in uninjured skin]) × 100

### Statistical analysis

All analyses were conducted using RStudio (version 2024.04.0 Build 735 [38]). The experimental unit for performance, physiological, and inflammatory outcomes was the individual piglet, while histopathological data were analyzed at the biopsy level (*n* = 5 piglets per treatment per timepoint). Variables not meeting normality assumption were transformed through Box-cox or log transformation.

PGE_2_ and haptoglobin concentrations were analyzed using generalized linear mixed models with a Gamma distribution and log link. Fixed effects included treatment, timepoint (day), and their interaction. Random intercepts accounted for piglets nested within litters. Baseline concentrations of the biomarkers were included as covariates, along with piglet weight, infrared body surface temperature (IRTd0) at enrollment. Also, sow parity, and litter characteristics (born alive, stillbirths, and mummified piglets) were used as covariables. Baseline haptoglobin levels were a strong positive predictor of post-treatment concentrations (*P* < 0.001), supporting their inclusion in the model as a covariate.

Infrared thermography and piglet body weight data were analyzed using linear mixed-effects models with the same fixed and random effect’s structure. Litter was modeled as a random intercept. Pre-weaning mortality was compared across treatments using Chi-square or Fisher’s exact tests, as appropriate.

Histopathological scores were analyzed using non-parametric Kruskal–Wallis tests followed by Dunn’s post hoc comparisons. A linear regression model was also fitted to the total histological score to explore associations with treatment, day, treatment x day interaction, and covariates. Least-squares means (LSMeans) were estimated, and pairwise comparisons were adjusted for multiple testing using Holm’s method. Statistical significance was set at α = 0.05.

## Results

### Piglet performance

**Fig. 2:**
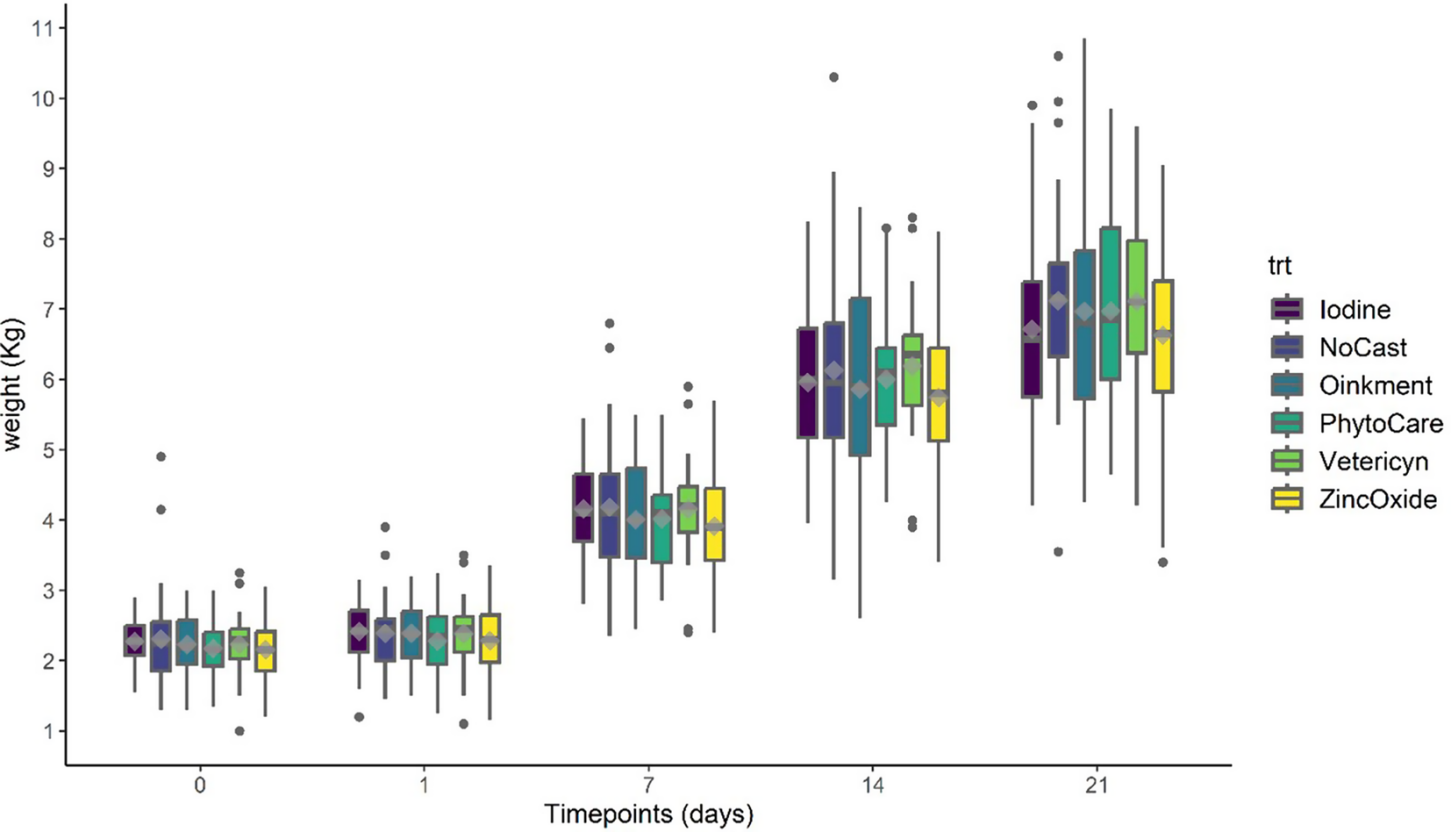
Least-squares mean body weight (kg ± SEM) of piglets across treatments and days. Castrated piglets were treated with Iodine (*n* = 32), Oinkment (*n* = 32), PhytoCare (*n* = 31), Vetericyn (*n* = 31), or Zinc Oxide (*n* = 32). The control group consisted of non-castrated (NoCast) piglets treated with iodine (*n* = 32). Statistical analysis showed no effect of treatment (*P* > 0.05), a significant effect of timepoint (*P* < 0.05), and no treatment × timepoint interaction (*P* > 0.05). Values are presented as least squares mean ± standard error of the mean (LSM ± SEM)

Sow reproductive parameters or piglet pre-weaning mortality (*P* > 0.05) did not differ between treatments; thus, these variables were excluded from subsequent models. Weight relative to castration is depicted in Fig. 2. Treatment and the interaction between treatment and day did not affect body weight over time (*P* > 0.05). However, an effect of day was observed (*P* < 0.05), indicating consistent growth across the 21-day period.

### Physiological response

#### Plasma prostaglandin E₂ (PGE₂)

**Fig. 3:**
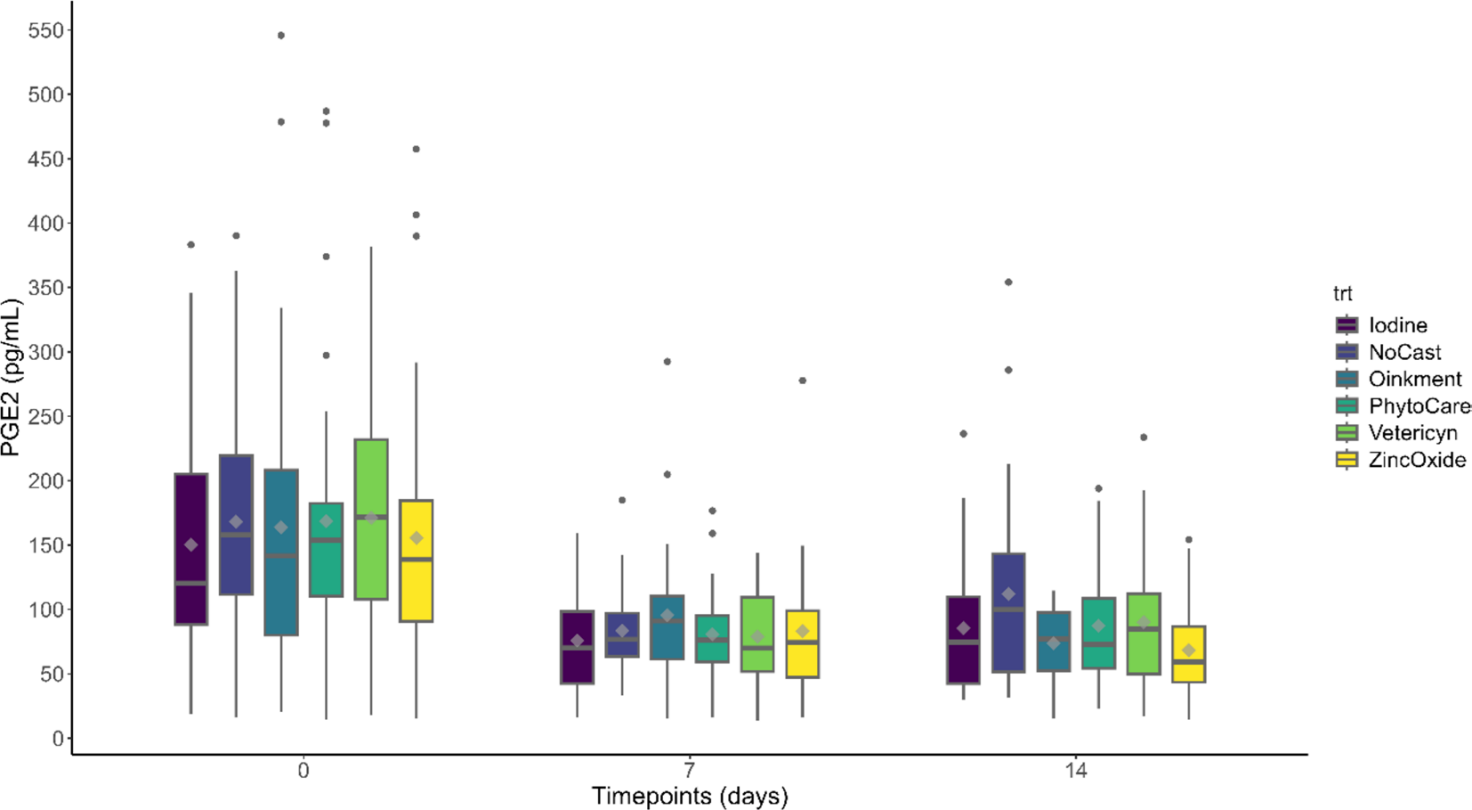
Least-squares mean prostaglandin E₂ (PGE₂) concentrations (pg/mL ± SEM) in piglets across treatments and days. *Castrated piglets were treated with Iodine (*n* = 32), Oinkment (*n* = 32), PhytoCare (*n* = 31), Vetericyn (*n* = 31), or Zinc Oxide (*n* = 32). The control group consisted of non-castrated (NoCast) piglets treated with iodine (*n* = 32). Statistical analysis showed no effect of treatment (*P* > 0.05), a significant effect of timepoint (*P* < 0.05), and no treatment × timepoint interaction (*P* > 0.05). Values are presented as least squares mean ± standard error of the mean (LSM ± SEM)

Plasma PGE₂ metabolite concentrations are presented in Fig. 3 (see Supplementary Table S1 for detailed LSM ± SEM values). A timepoint effect was observed, with PGE₂ concentrations decreasing on day 7 (*P* < 0.001) and day 14 (*P* < 0.001) relative to baseline (day 0). Treatment (*P* > 0.05) or treatment × day interaction (*P* > 0.05) effects were not detected.

#### Plasma haptoglobin concentrations

**Fig. 4:**
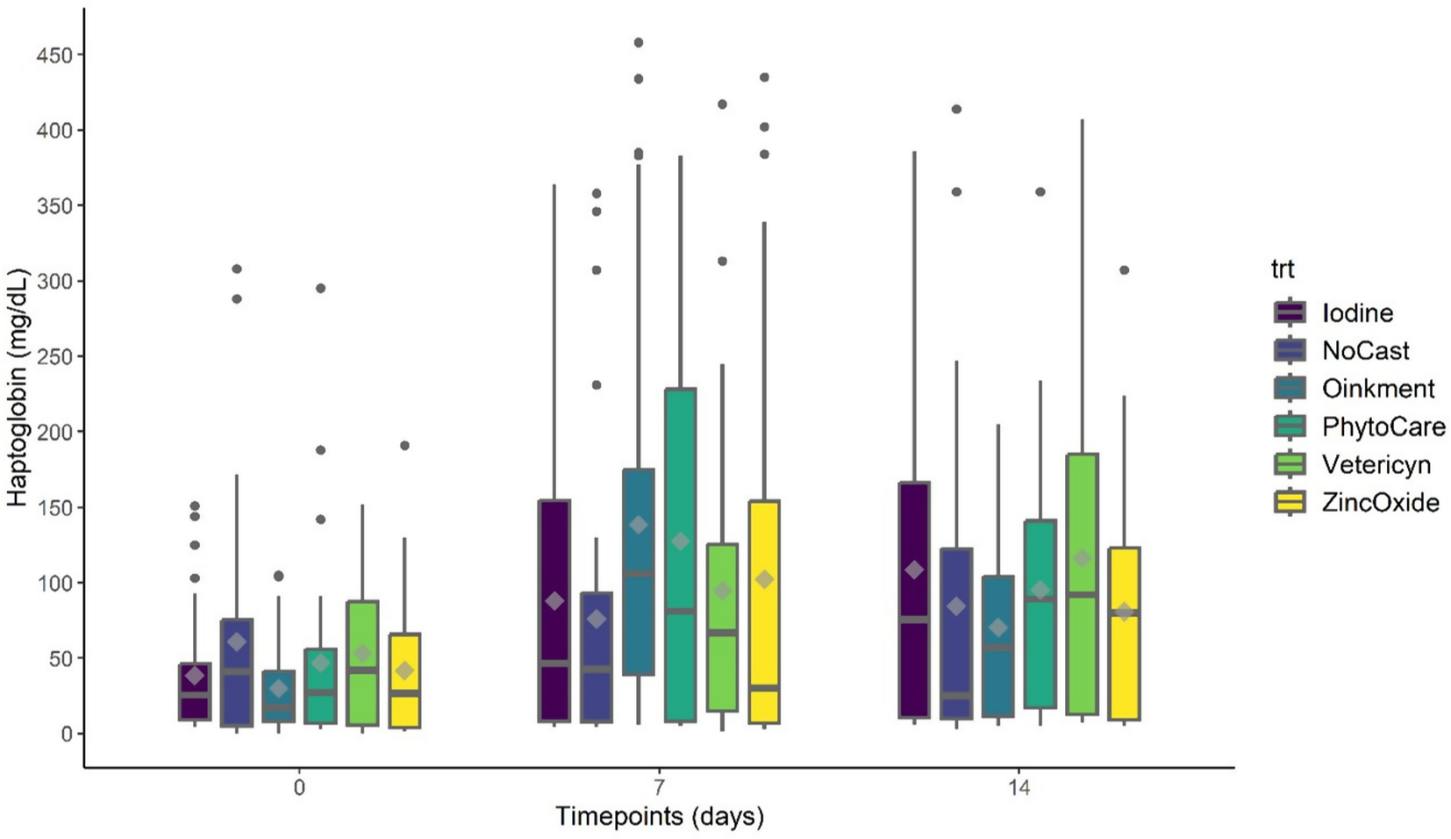
Least-squares mean plasma haptoglobin concentrations (mg/dL ± SEM) in piglets across treatments and timepoints. *Castrated piglets were treated with Iodine (*n* = 32), Oinkment (*n* = 32), PhytoCare (*n* = 31), Vetericyn (*n* = 31), or Zinc Oxide (*n* = 32). The control group consisted of non-castrated (NoCast) piglets treated with iodine (*n* = 32). Statistical analysis showed no effect of treatment (*P* > 0.05), a significant effect of timepoint (*P* < 0.05), and no treatment × timepoint interaction (*P* > 0.05). Values are presented as least squares mean ± standard error of the mean (LSM ± SEM)

Plasma haptoglobin concentrations (mg/dL) are shown in Fig. 4 (see Supplementary Table S2 for detailed LSM ± SEM values). No effects were detected for treatment (*P* > 0.05) or for the treatment × timepoint interaction (*P* > 0.05). However, a timepoint effect was observed, with haptoglobin levels elevated on both day 7 (*P* < 0.001) and day 14 (*P* < 0.001) compared to day 0.

### Inflammatory response

**Fig. 5:**
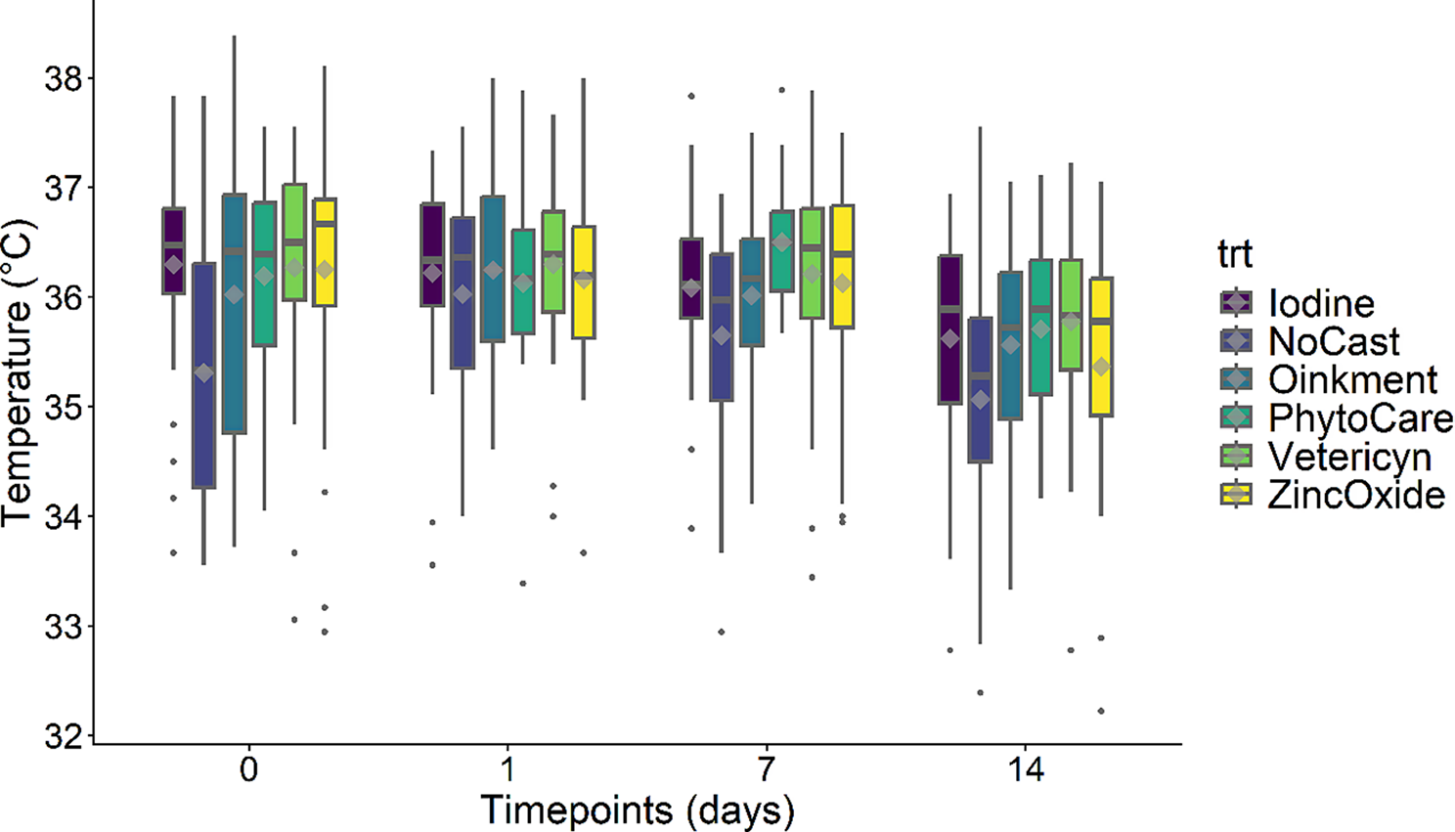
Least-squares mean skin surface temperature (°C ± SEM) measured via infrared thermography in piglets across treatments and timepoints. *Castrated piglets were treated with Iodine (*n* = 32), Oinkment (*n* = 32), PhytoCare (*n* = 31), Vetericyn (*n* = 31), or Zinc Oxide (*n* = 32). The control group consisted of non-castrated (NoCast) piglets treated with iodine (*n* = 32). Statistical analysis showed an effect of day (*P* < 0.05). Values are presented as least squares mean ± standard error of the mean (LSM ± SEM)

Figure 5 displays the measurements obtained from infrared thermography (IRT). A significant effect was detected for day (*P* < 0.05), but treatment (*P* > 0.05) and the treatment × day interaction (*P* > 0.05) were not significant. A marginal reduction in IRT overtime was observed, not attributable to the treatments. Baseline temperature (D0) was a significant positive predictor, underscoring the need to account for initial differences.

### Histopathological response

Histopathological measurements collected on days 7 and 14 are summarized in Table 3 Treatment (*P* > 0.05) or treatment × day interaction (*P* > 0.05) effects were not detected for any of the evaluated histological parameters. However, a timepoint effect was observed (*P* < 0.05) for epidermal thickness index, ulceration, serocellular crusting, granulation tissue, and total combined histological score, with all measures showing a reduction on day 14 compared to day 7, consistent with normal wound healing progression.

**Table 3:** Effect of treatment on histopathological wound healing parameters in castrated piglets on days 7 and 14 (mean ± SEM)

Variables	Treatment													*P*-values		
	Iodine		Oinkment		PhytoCare			Vetericyn			ZincOxide					
	D7	D14	D7	D14	D7	D14		D7	D14		D7	D14		Trt	Day	Trt*Day
Epidermal Ulceration^a^	1.09 ± 0.36	0.41 ± 0.33	1.42 ± 0.36	0.10 ± 0.33	0.00 ± 0.36	0.00 ± 0.33	1.49 ± 0.36		0.43 ± 0.33	0.70 ± 0.36		0.46 ± 0.33	0.143		0.008	0.354
Epidermal Thickness Index^b^	2.80 ± 0.27	2.02 ± 0.39	2.82 ± 0.27	1.57 ± 0.39	3.01 ± 0.27	1.80 ± 0.39	2.22 ± 0.27		2.24 ± 0.39	2.25 ± 0.27		1.92 ± 0.39	0.572		0.003	0.305
Serocellular Crusting^c^	1.83 ± 0.50	0.63 ± 0.35	1.83 ± 0.50	0.63 ± 0.35	1.60 ± 0.50	0.01 ± 0.35	1.59 ± 0.50		0.51 ± 0.35	1.97 ± 0.50		0.46 ± 0.35	0.664		< 0.001	0.974
Inflammatory Infiltrate^d^	1.21 ± 0.38	1.06 ± 0.41	1.69 ± 0.38	0.71 ± 0.41	0.86 ± 0.38	1.25 ± 0.41	1.18 ± 0.38		1.25 ± 0.41	1.92 ± 0.38		1.18 ± 0.41	0.717		0.305	0.476
Granulation Tissue^e^	2.42 ± 0.36	1.81 ± 0.33	2.25 ± 0.36	1.39 ± 0.33	1.81 ± 0.36	1.38 ± 0.33	2.04 ± 0.36		2.04 ± 0.33	2.46 ± 0.36		1.21 ± 0.33	0.644		0.010	0.490
Dermal Hemorrhage^f^	1.18 ± 0.28	0.43 ± 0.38	0.63 ± 0.28	1.04 ± 0.38	0.35 ± 0.28	0.75 ± 0.38	0.59 ± 0.28		1.19 ± 0.38	0.23 ± 0.28		0.98 ± 0.38	0.452		0.214	0.226
Epidermal Thickness (µm)	152.37 ± 16.61	148.37 ± 18.57	149.87 ± 10.06	125.08 ± 7.78	152.13 ± 21.50	102.76 ± 6.36	159.67 ± 29.11		116.28 ± 13.50	128.03 ± 17.03		140.21 ± 30.65	0.421		0.229	0.227
Combined Score	10.36 ± 1.49	6.33 ± 1.59	10.54 ± 1.49	5.46 ± 1.59	7.57 ± 1.49	5.12 ± 1.59	9.01 ± 1.49		7.65 ± 1.59	9.47 ± 1.49		6.22 ± 1.59	0.716		0.003	0.809

^a^ 0: intact (< 5%); 1: partial (5–25%); 2: ulceration (25–50%); 3: complete ulceration (> 50%); ^b^ 0: normal (95–105%); 1: mild (< 110%); 2: moderate (110–120%); 3: marked (> 120%) w/ a r p; ^c^ 0: absent; 1: mild (25–50%); 2: moderate (50–75%); 3: marked (> 75%); ^d^ 0: minimal; 1: mild; 2: moderate; 3: marked; ^e^ 0: none/minimal; 1: mild (10–25%), 2: moderate (25–50%); 3: proliferative (> 50%); ^f^ 0: absent; 1 mild; 2: moderate;3: marked. D7: day 7. D14: day 14. *P*-values represent results from Kruskal–Wallis tests for treatment, day, and treatment × day interaction effects. Superscripts were not used due to the non-parametric nature of the analysis

## Discussion

This study investigated the efficacy of five commercially available topical products in mitigating inflammation and promoting wound healing following surgical castration in piglets under commercial conditions. Surgical castration is a common procedure routinely conducted on a global scale with limited research available regarding effective topical formulations on the castration wound healing. Previous work to this study has largely focused on topical formulations used to reduce acute pain at the time of castration [39–42] while more current work has begun evaluating how topical application of products did or did not effectively support tissue repair or healing [27, 43]. Although some work has explored specific products, to the author’s knowledge, no studies have evaluated these specific commercially available products included in this study. Therefore, this study provides practical insight into how commonly used topical products perform under commercial conditions, contributing to the broader understanding of post-castration wound-care strategies.

Across treatments, no effects were observed on growth performance or pre-weaning survival, suggesting that topical applications did not negatively impact pre-weaning performance. However, castrated piglets maintained similar weights to intact piglets that did not undergo castration. Although it is well established that castration causes growth setbacks [17, 22], our results did not capture these differences. Our findings are similar to Kielly’s et al. [44] who reported weight loss in the first days post-castration but no differences at weaning. In our study, piglets were weighed on a weekly basis, which may have limited our ability to detect subtle transient changes in growth performance occurring within shorter intervals post-procedure. Future studies should include more frequent weight records (e.g., daily in the immediate post-castration period) to better capture growth trajectories.

In addition to the absence of differences in performance measures, inflammatory measures also did not differ among treatment groups, and PGE₂ levels declined across all groups, including non-castrated piglets. This finding aligns with previous studies [10, 32] and suggests that PGE₂ is not a reliable post-operative inflammatory biomarker in neonatal pigs [29, 45]. Its elevated baseline levels may reflect physiological processes such as intestinal development, microbial colonization, or transient neonatal immune activity of pre-weaned piglets, despite undergoing castration [29]. These results indicate that while PGE₂ plays a central role in inflammation, its diagnostic value in early-life castration models is limited.

Similar to PGE_2_ results, haptoglobin did not demonstrate sensitivity as an indicator of castration inflammation. Haptoglobin is an acute-phase protein synthesized in the liver and commonly used as an indicator of systemic inflammation and tissue injury [46]. In this study, haptoglobin concentrations increased significantly by Day 7 and remained elevated on day 14 across all groups, including intact piglets. This pattern indicates that the observed response may not be attributable to castration-related inflammation. Instead, increases could reflect other early-life factors, such as social stress, androgen-driven immune modulation in intact males [47], or subclinical infections [48]. These findings highlight the multifactorial nature of haptoglobin responses in neonatal pigs and suggest it may lack specificity as a biomarker for castration associated inflammation. Future work should consider alternative biomarkers with greater sensitivity such as C-reactive proteins, serum amyloid A [49] or pro-inflammatory cytokines such as IL-1β and TNF-α [50], to more precisely characterize the inflammatory response to castration.

Blood samples were taken from the orbital sinus according to a previously published technique in piglets [29–32, 51, 52], nursery and growing pigs [53, 54] and sows [55]. This well-described procedure has been validated to provoke minimal invasion with bleeding ceasing within seconds and animals resuming normal behavior within minutes. Additionally, because the protocol was applied to all groups (including non-castrated pigs), any transient sampling effect would be nondifferential and therefore improbable to bias between-group comparisons. Finally, we observed expected time-dependent trajectories (e.g., PGE₂ declining over time in all groups; haptoglobin rising at D7 and D14) without treatment effects. These patterns occurred equally in the non-castrated group that were sampled identically, suggesting the biomarker dynamics were not driven by the sampling technique per se.

Infrared thermography (IRT) proved to be a non-invasive and sensitive method for distinguishing inflammatory responses between castrated and intact piglets, however there was no treatment effect, demonstrating that the products did not drive changes to scrotal temperature, but the castration procedure itself.

Castrated piglets consistently exhibited higher skin surface temperatures than their intact counterparts. This is likely due to local inflammation and increased blood perfusion in response to tissue injury [56], consistent with previous work demonstrating that IRT can detect thermal changes associated with castration or other surgical wounds in livestock and companion animals [21, 57–59]. However, IRT changes may not be as responsive to systemic changes and future studies should consider the use of rectal thermography to detect more subtle differences between treatments.

In addition, for this study, spermatic cords were torn instead of cutting based on the approved SOP of the commercial farm. This is a common practice in the U.S., although we acknowledge that in many European countries tearing of spermatic cords instead of cutting is not allowed. Work conducted by Schmid and colleagues [19] demonstrated that piglet behavior is altered with tearing and the tearing technique may result in further inflammation and tissue damage. Because all piglets underwent the same handling, sampling schedule, and farm-mandated castration technique, we cannot entirely rule out that the inherent inflammatory responses associated with these procedures contributed to baseline variability in physiological markers.

Histopathological evaluation revealed time-dependent improvements in wound healing between days 7 and 14, including reductions in ulceration, crusting, granulation tissue, and overall wound scores of all piglets. Epidermal thickness was the only histological variable correlated with total wound severity, suggesting it may be a useful proxy for local inflammatory activity [60] and could potentially serve as a standalone measure for wound healing. Although histopathologic results demonstrated wound healing improvement over the two weeks, no treatment effect was observed. These findings indicate that none of the tested products significantly impaired or enhanced the healing process under the application conditions used. However, without the inclusion of a true control group (castrated piglets without treatment), it is difficult to quantify the true effect of these products.

Limitations of this work should be acknowledged. First, the lack of treatment effects may reflect the single application protocol and limited sample size of the study. A larger sample size and an experimental design permitting repeated or long-term application of the product should be considered. In addition, while physiological and histological measures were included, behavioral and microbiological assessments, including irritability, were not incorporated, limiting the scope of welfare and infection-related insights. Finally, our study design did not include a group of castrated piglets, receiving no wound care, preventing direct evaluation of whether topical treatments confer advantages compared to no intervention. The current SOP for castration on farm requires piglets to receive Iodine as topical antiseptic after castration to reduce infection risk and support wound cleanliness. Moreover, the aim of this trial was to evaluate different commercially available topical products in the livestock industry versus evaluating the outcome of treated versus non-treated. For this reason, all castrated piglets were assigned to one of the topical wound care treatment groups.

## Conclusions

Minimal differences were noted when comparing five commercially available topical products on the impact wound healing, inflammatory responses, and growth performance in piglets undergoing surgical castration. Neither haptoglobin nor prostaglandin E_2_ were effective biomarkers for distinguishing castration-related inflammation in this context, whereas infrared thermography effectively detected post-castration thermal changes associated with tissue injury. Histological healing progressed over time in all groups, with epidermal thickness providing a consistent measure of wound response. All products appeared safe under a single-application protocol and did not impair growth; however, their benefits relative to one another were limited. Importantly, because farm standard operating procedures required the use of iodine on all castrated piglets, this study could not include a castrated untreated control group. Therefore, the findings do not allow conclusions about whether any product improves or impairs wound healing compared to no topical intervention. Future research should incorporate a no-treatment control group and expand assessments to include behavioral indicators of pain and healing, as well as microbiological characterization of incisional sites, to more comprehensively evaluate product performance.

## Supplementary Information

Below is the link to the electronic supplementary material.

Supplementary Material 1

Supplementary Material 2

Supplementary Material 3

Supplementary Material 4

Supplementary Material 5

Supplementary Material 6

## Data Availability

The data that support the findings of this study are available from the corresponding author upon reasonable request.

## References

[1] Weiler U, Font-i-Furnols M, Tomasevič I, Bonneau M. Alternatives to piglet castration: from issues to solutions. Animals. 2021;11:1041. 10.3390/ani11041041. 33917235 10.3390/ani11041041 PMC8067991

[2] Breitenlechner A, Bünger M, Ruczizka UK, Dolezal M, Auer U, Buzanich-Ladinig A. Comparison between intramuscular and intranasal administration of sedative drugs used for piglet castration. Animals. 2024;14:2325. 10.3390/ani14162325. 39199860 10.3390/ani14162325 PMC11350754

[3] Morgan L, Itin-Shwartz B, Koren L, Meyer JS, Matas D, Younis A, Novak S, Weizmann N, Rapaic O, Ahmad WA, Klement E, Raz T. Physiological and economic benefits of abandoning invasive surgical procedures and enhancing animal welfare in swine production. Sci Rep. 2019;9:16093. 10.1038/s41598-019-52677-6. 31695123 10.1038/s41598-019-52677-6 PMC6834556

[4] Miller R, Grott A, Patzkéwitsch D, Döring D, Abendschön N, Deffner P, Reiser J, Ritzmann M, Saller AM, Schmidt P, Senf S, Werner J, Baumgartner C, Zols S, Erhard M, Bergmann S. Behavior of piglets in an observation arena before and after surgical castration with local anesthesia. Animals. 2023;13:529. 10.3390/ani13030529. 36766418 10.3390/ani13030529 PMC9913414

[5] Vanhonacker F, Verbeke W, Tuyttens F. Belgian consumers’ attitude towards surgical castration and immunocastration of piglets. Anim Welf. 2009;18(4):371–80. 10.1017/S0962728600000774.

[6] Hötzel MJ, Yunes MC, Vandresen B, Albernaz-Gonçalves R, Woodroffe RE. On the road to end pig pain: knowledge and attitudes of Brazilian citizens regarding castration. Animals 2020; 10(10), 1826. 10.3390/ani10101826. 33049950 10.3390/ani10101826 PMC7650544

[7] Hokkanen AH, Coutant M, Heinonen M, Norring M, Adam M, Oliviero C, Bergqvist T, Valros A. Two restraining devices in connection to surgical castration with or without local anesthesia: effects on piglet stress. Porc Health Manag. 2025;11:21. 10.1186/s40813-025-00428-7. 10.1186/s40813-025-00428-7 PMC12001485 40234960

[8] Rault JL, Lay DC Jr., Marchant JN. Castration-induced pain in pigs and other livestock. Appl Anim Behav Sci. 2011;135:214–25. 10.1016/j.applanim.2011.10.017.

[9] Coutant M, Malmkvist J, Kaiser M, Foldager L, Herskin MS. Piglets’ acute responses to local anesthetic injection and surgical castration: effects of the injection method and interval between injection and castration. Front Vet Sci. 2022;9:1009858. 10.3389/fvets.2022.1009858. 36246321 10.3389/fvets.2022.1009858 PMC9556771

[10] Nixon E, Carlson AR, Routh PA, Hernandez L, Almond GW, Baynes RE, Messenger KM. Comparative effects of nonsteroidal anti-inflammatory drugs at castration and tail-docking in neonatal piglets. PLoS ONE. 2021;16:e0254409. 10.1371/journal.pone.0254409. 34847143 10.1371/journal.pone.0254409 PMC8631668

[11] Hansson M, Lundeheim N, Nyman G, Johansson G. Effect of local anaesthesia and/or analgesia on pain responses induced by piglet castration. Acta Vet Scand. 2011;53:34. 10.1186/1751-0147-53-34. 10.1186/1751-0147-53-34 PMC3123560 21627797

[12] Hay M, Vulin A, Génin S, Sales P, Prunier A. Assessment of pain induced by castration in piglets: behavioral and physiological responses over the subsequent 5 days. Appl Anim Behav Sci. 2003;82(3):201–18. 10.1016/S0168-1591(03)00059-5.

[13] Robles I, Luna SP, Trindade PH, Lopez-Soriano M, Merenda VR, Viscardi AV, et al. Validation of the Unesp-Botucatu pig composite acute pain scale (UPAPS) in piglets undergoing castration. PLoS ONE. 2023;18(4):e0284218. 10.1371/journal.pone.0284218. 37053294 10.1371/journal.pone.0284218 PMC10101451

[14] Adcock SJJ. Early life painful procedures: Long-Term consequences and implications for farm animal welfare. Front Anim Sci 2021; 2:759522. 10.3389/fanim.2021.759522.

[15] Sandercock DA, Barnett MW, Coe JE, Downing AC, Nirmal AJ, Di Giminiani P, Edwards SA, Freeman TC. Transcriptomics analysis of Porcine caudal dorsal root ganglia in tail-amputated pigs shows long-term effects on many pain-associated genes. Front Vet Sci. 2019;6:314. 10.3389/fvets.2019.00314. 31620455 10.3389/fvets.2019.00314 PMC6760028

[16] Schmid SM, Steinhoff-Wagner J. Impact of routine management procedures on the welfare of suckling piglets. Veterinary Sci 2022; 9(1), 32. 10.3390/vetsci9010032. 10.3390/vetsci9010032 PMC8778417 35051116

[17] Guay K, Salgado G, Thompson G, Backus B, Sapkota A, Chaya W, McGlone JJ. Behavior and handling of physically and immunologically castrated market pigs on farm and going to market. J Anim Sci. 2013;91:5410–7. 10.2527/jas.2012-5726. 24045467 10.2527/jas.2012-5726

[18] Morales J, Dereu A, Manso A, de Frutos L, Piñeiro C, Manzanilla EG. Wuyts, N. Surgical castration with pain relief affects the health and productive performance of pigs in the suckling period. Porc Health Manag. 2017;3:18. 10.1186/s40813-017-0066-1. 10.1186/s40813-017-0066-1 PMC5585944 28879020

[19] Schmid SM, Genter CI, Heinemann C, Steinhoff-Wagner J. Impact of tearing spermatic cords during castration in live and dead piglets and consequences on welfare. Porc Health Manag. 2021;7:17. 10.1186/s40813-021-00200-7. 10.1186/s40813-021-00200-7 PMC7883445 33583429

[20] AVMA (American Veterinary Medical Association). Literature review on the welfare implications of swine castration. 2013. Available at: https://www.avma.org/KB/Resources/LiteratureReviews/Documents/swine_castration_bgnd.pdf.

[21] Viscardi AV, Cull CA, Kleinhenz MD, Montgomery S, Curtis A, Lechtenberg K, Coetzee JF. Using a CO2 surgical laser for piglet castration to reduce pain and inflammation, and to improve wound healing. Kans Agric Exp Stn Res Rep. 2020;6:10. 10.4148/2378-5977.8016. 10.1093/jas/skaa320 PMC7660141 33011759

[22] Telles FG, Luna SPL, Teixeira G, Berto DA. Long-term weight gain and economic impact in pigs castrated under local anaesthesia. Vet Anim Sci. 2016;1:36–9. 10.1016/j.vas.2016.11.003. 32734022 10.1016/j.vas.2016.11.003 PMC7386683

[23] Mellor DJ, Beausoleil NJ. Extending the ‘Five domains’ model for animal welfare assessment to incorporate positive welfare States. Anim Welf. 2015;24:241–53. 10.7120/09627286.24.3.241.

[24] Bigliardi PL, Alsagoff SAL, El-Kafrawi HY, Pyon JK, Wa CTC, Villa MA. Povidone iodine in wound healing: A review of current concepts and practices. Int J Surg. 2017;44:260–8. 10.1016/j.ijsu.2017.06.073. 28648795 10.1016/j.ijsu.2017.06.073

[25] Gooch JW. Barrier dressings for wounds. In: biocompatible polymeric materials and tourniquets for wounds. Topics in applied chemistry. Springer, 2010. Available at: https://primo.qatar-weill.cornell.edu/permalink/974WCMCIQ_INST/ked4uo/alma991000154839706691.

[26] Jacobs L, Neary J. Castration in the U.S. swine industry: animal welfare implications and alternatives. 2020. Available at: http://hdl.handle.net/10919/105585.

[27] Garcia A, Sutherland M, Vasquez G, Quintana A, Thompson G, Willis J, Chandler S, Niure K, McGlone J. An investigation of the use of Ethyl chloride and meloxicam to decrease the pain associated with a single or double incision method of castration in piglets. Front Pain Res. 2023;4:1113039. 10.3389/fpain.2023.1113039. 10.3389/fpain.2023.1113039 PMC10416629 37575637

[28] FASS (Federation of Animal Science Societies). Guide for the care and use of agricultural animals in agricultural research and teaching, 4th ed. 2020. Available at: https://www.asas.org/docs/default-source/default-document-library/agguide_4th.pdf?sfvrsn=56b44ed1_2.

[29] Merenda VR, Lopez-Soriano M, Anderson S, Trindade PHE, Tomacheuski RM, Leidig MS, Messenger K, Ferreira JB, Pairis-Garcia MD. Prostaglandin E2 is an unreliable biomarker for inflammation in castrated piglets: A randomized controlled trial assessing pharmaceutical drug efficiency. Am J Vet Res. 2024;85:96. 10.2460/ajvr.24.04.0096. 10.2460/ajvr.24.04.0096 39047790

[30] Dove CR, Alworth LC. Blood collection from the orbital sinus of swine. Lab Anim (NY). 2014;44:383–4. 10.1038/laban.869. 10.1038/laban.869 26398611

[31] Merenda VR, Wagner BK, Arruda AG, Lopez Soriano M, Montgomery S, Coetzee JF, Pairis-Garcia MD. Impact of transdermal flunixin administration on serum prostaglandin E₂ and cortisol concentrations in piglets following castration. Am J Vet Res. 2022;83(9):ajvr.21.12.0201. 10.2460/ajvr.21.12.0201. 10.2460/ajvr.21.12.0201 35895772

[32] Lopez-Soriano M, Merenda VR, Anderson S, Trindade PHE, Leidig MS, Messenger K, Ferreira JB, Pairis-Garcia MD. Efficacy of inguinal buffered Lidocaine and intranasal flunixin Meglumine on mitigating physiological and behavioral responses to pain in castrated piglets. Front Pain Res. 2023;4:1156873. 10.3389/fpain.2023.1156873. 10.3389/fpain.2023.1156873 PMC10279844 37346473

[33] Giorgi M, Cuniberti B, Ye G, Barbero R, Sgorbini M, Vercelli C, Corazza M, Re G. Oral administration of Tepoxalin in the horse: A PK/PD study. Vet. J. 2011;190:143–9. 10.1016/j.tvjl.2010.09.013. 10.1016/j.tvjl.2010.09.013 21036634

[34] Bates JL, Karriker LA, Stock ML, Pertzborn KM, Baldwin LG, Wulf LW, Lee CJ, Wang C, Coetzee JF. Impact of transmammary-delivered meloxicam on biomarkers of pain and distress in piglets after castration and tail Docking. PLoS ONE. 2014;9:e113678. 10.1371/journal.pone.0113678. 25437866 10.1371/journal.pone.0113678 PMC4249978

[35] Hackworth C. (2019). Skin punch biopsy: An overview for the veterinary nurse. Kansas State Veterinary Diagnostic Laboratory; 2019. Available at: https://ksvdl.org/resources/news/diagnostic_insights_for_technicians/august2019/skin-punch-biopsy.htmL.

[36] Santos TS, Santos IDDD, Pereira-Filho RN, Gomes SVF, Lima-Verde IB, Marques MN, Cardoso JC, Severino P, Souto EB, Albuquerque-Júnior RLC. Histological evidence of wound healing improvement in rats treated with oral administration of hydroalcoholic extract of vitis Labrusca. Curr Issues Mol Biol. 2021;43:335–52. 10.3390/cimb43010028. 34208147 10.3390/cimb43010028 PMC8929082

[37] van de Vyver M, Boodhoo K, Frazier T, Hamel K, Kopcewicz M, Levi B, Maartens M, Machcinska S, Nunez J, Pagani C, Rogers E, Walendzik K, Wisniewska J, Gawronska-Kozak B, Gimble JM. Histology scoring system for murine cutaneous wounds. Stem Cells Dev. 2021;30:1141–52. 10.1089/scd.2021.0124. 34130483 10.1089/scd.2021.0124 PMC9022171

[38] R Core Team. RStudio 2024.04.0 + 735. R: A language and environment for statistical computing. R Foundation for Statistical Computing, Vienna, Austria. 2024. https://www.R-project.org/.

[39] Sutherland MA, Davis BL, Brooks TA, McGlone JJ. Physiology and behavior of pigs before and after castration: effects of two topical anesthetics. Animal. 2010;4:2071–9. 10.1017/S1751731110001291. 22445382 10.1017/S1751731110001291

[40] Gottardo F, Scollo A, Contiero B, Ravagnani A, Tavella G, Bernardini D, De Benedictis GM, Edwards SA. Pain alleviation during castration of piglets: A comparative study of different farm options. J Anim Sci. 2016;94:5077–88. 10.2527/jas.2016-0843. 28046151 10.2527/jas.2016-0843

[41] Lomax S, Harris C, Windsor PA, White PJ. Topical anaesthesia reduces sensitivity of castration wounds in neonatal piglets. PLoS ONE. 2017;12:e0187988. 10.1371/journal.pone.0187988. 29140997 10.1371/journal.pone.0187988 PMC5687763

[42] Sheil ML, Chambers M, Sharpe B. Topical wound anaesthesia: efficacy to mitigate piglet castration pain. Aust Vet J. 2020;98:77–83. 10.1111/avj.12930. 10.1111/avj.12930 PMC7384076 32096229

[43] Prokop D, Spergser J, Hagmüller W, Tichy A, Zitterl-Eglseer K. Efficacy of Norway Spruce ointments and bacterial and fungal alterations in the treatment of castration wounds in piglets. Planta Med. 2021;88:300–12. 10.1055/a-1646-2959. 34624905 10.1055/a-1646-2959

[44] Kielly J, Dewey CE, Cochran M. Castration at 3 days of age temporarily slows growth of pigs. Swine Health Prod. 1999; 7:151–153. Available at: https://www.aasv.org/shap/issues/v7n4/v7n4p151.pdf.

[45] Everaert N, Van Cruchten S, Weström B, Bailey M, Van Ginneken C, Thymann T, Pieper R. A review on early gut maturation and colonization in pigs, including biological and dietary factors affecting gut homeostasis. Anim Feed Sci Technol. 2017;233:89–103. 10.1016/j.anifeedsci.2017.06.011.

[46] Merlot E, Thomas F, Prunier A. Comparison of immune and health markers in intact and neonatally castrated male pigs. Vet Rec. 2013;173:317. 10.1136/vr.101667. 24043704 10.1136/vr.101667

[47] Fardisi M, Thelen K, Groenendal A, Rajput M, Sebastian K, Contreras GA, Moeser AJ. Early weaning and biological sex shape long-term immune and metabolic responses in pigs. Sci Rep. 2023;13:15907. 10.1038/s41598-023-42553-9. 37741873 10.1038/s41598-023-42553-9 PMC10517948

[48] Saco Y, Bassols A. Acute phase proteins in cattle and swine: A review. Vet Clin Pathol. 2022. 10.1111/vcp.13220. 36526287 10.1111/vcp.13220

[49] Pomorska-Mól M, Markowska-Daniel I, Kwit K, Stępniewska K, Pejsak ZC. -reactive protein, haptoglobin, serum amyloid A and pig major acute phase protein response in pigs simultaneously infected with H1N1 swine influenza virus and pasteurella multocida. BMC Vet Res. 2013;9:14. 10.1186/1746-6148-9-14. 23332090 10.1186/1746-6148-9-14 PMC3554491

[50] Llamas Moya S, Boyle LA, Lynch PB, Arkins S. Surgical castration of pigs affects the behavioural response to a low-dose lipopolysaccharide (LPS) challenge after weaning. Appl Anim Behav Sci. 2008;112:40–57. 10.1016/j.applanim.2007.07.001.

[51] Friend DW, Brown RG. Blood sampling from suckling piglets. Anim Res Inst Can Dept Agric. 1971;51:547–9. https://cdnsciencepub.com/doi/pdf/10.4141/cjas71-074?download=true. (Contribution 407).

[52] Reynolds K, Johnson R, Brown J, Friendship R, O’Sullivan TL. Assessing pain control efficacy of meloxicam and ketoprofen when compounded with iron dextran in nursing piglets using a navigation chute. Animals. 2020;10(7):1237. 10.3390/ani10071237. 32708287 10.3390/ani10071237 PMC7401524

[53] Huhn RG, Osweiler GD, Switzer WP. Application of the orbital sinus bleeding technique to swine. Lab Anim Care. 1969;19(3):403–5. 10.6084/m9.figshare.31215859. 4240473

[54] Henry M, Shoveller AK, O’Sullivan TL, Niel L, Friendship R. Effect of varying levels of dietary Tryptophan on aggression and abnormal behavior in growing pigs. Front Vet Sci. 2022;9:849970. 10.3389/fvets.2022.849970. 35720838 10.3389/fvets.2022.849970 PMC9198587

[55] Hill SV, Amezcua MDR, Ribeiro ES, O’Sullivan TL, Friendship RM. Sow hematological parameters in late pregnancy and an investigation as to whether these parameters are predictors of stillbirths in a Canadian Sow herd. Can Vet J. 2024;65(1):42–8. PMCID: PMC10727158. https://pmc.ncbi.nlm.nih.gov/articles/pmid/38164378/. 38164378 PMC10727158

[56] Korkmaz HI, Ulrich MMW, van Wieringen WN, Vlig M, Emmens RW, Meyer KW, Sinnige P, Krijnen PAJ, van Zuijlen PPM, Niessen HWM. The local and systemic inflammatory response in a pig burn wound model with a pivotal role for complement. J Burn Care Res. 2017;38:e796–806. 10.1097/BCR.0000000000000486. 28447971 10.1097/BCR.0000000000000486

[57] Stewart M, Verkerk GA, Stafford KJ, Schaefer AL, Webster JR. Noninvasive assessment of autonomic activity for evaluation of pain in calves, using surgical castration as a model. J Dairy Sci. 2010;93:3602–9. 10.3168/jds.2010-3114. 20655429 10.3168/jds.2010-3114

[58] Bergamasco L, Edwards-Callaway LN, Bello NM, Mijares SH, Cull CA, Rugan S, Mosher RA, Gehring R, Coetzee JF. Unmitigated surgical castration in calves of different ages: cortisol concentrations, heart rate variability, and infrared thermography findings. Animals. 2021;11:2719. 10.3390/ani11092719. 34573687 10.3390/ani11092719 PMC8469829

[59] Saidu AM, Olorunfemi OJ, Laku D. Infrared thermography following castration, otectomy and gastrotomy in Nigerian Indigenous dogs. Sahel J Vet Sci. 2023;20:50–6. 10.54058/saheljvs.v20i1.373.

[60] Greaves P. Integumentary system. In: Greaves P, editor. Histopathology of preclinical toxicity studies. 4th ed. Academic; 2012. pp. 11–68. 10.1016/C2010-0-67226-9.

