# Supplementary material for: Topical wound-care products and their effects on healing, inflammatory biomarkers, and growth in piglets undergoing castration
Source: Porcine Health Manag. 2026 Apr 21;12:23. doi: 10.1186/s40813-026-00492-7 (PMC13097753; doi:10.1186/s40813-026-00492-7)
Supplement: Supplementary file 1 — Supplementary Material 1 [file 40813_2026_492_MOESM1_ESM.pdf]

INDICATIONS: A topical antiseptic for use on horses, cattle, swine and sheep prior to surgical procedures such as castrating and docking, for application to the navel of newborn animals; and for aid in the treatment of minor cuts, bruises, and abrasions.

DIRECTIONS: Remove spray cap from 16 oz. container and refill from gallon container. Replace spray cap tightly. Hold container approximately 4 to 6 inches from the area to be treated. Point valve at the area to be sprayed. Pull trigger of valve and spray area once lightly. May be repeated daily, when necessary, until abraded area is healed.

CAUTION: If redness, irritation, or swelling persists or increases, discontinue use and consult a veterinarian. Not for use on burns or in body cavities or deep wounds.

NOTE: When used on or near the teats or udders of dairy animals, the teats and udders should be thoroughly washed before the next milking to prevent contamination of milk.

® Registered Trademark of Agri Laboratories, Ltd.

Manufactured for:
Agri Laboratories, Ltd.
St. Joseph, MO 64503

LOT

EXP

NDC 57561-027-80

IODINE WOUND SPRAY

TOPICAL ANTISEPTIC

NET CONTENTS:
1 GALLON (3.785 Liters)

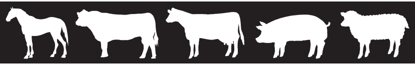

AgriLabs®
Making A Healthy Difference®

Active Ingredients:

Alpha (p-Nonylphenyl) Omega-Hydroxypoly (Oxyethylene) Iodine Complex (providing 1% Minimum Titratable Iodine) 5%

Inactive Ingredients

Water and Nonionic Surfactant 95%

CAUTION: Harmful if swallowed. Do not apply to the eyes, mucous membranes or large areas of abraded skin. Avoid inhalation of mist.

HAZARDOUS: Livestock remedy. Not for human use.

FOR EXTERNAL ANIMAL USE ONLY.
KEEP OUT OF REACH OF CHILDREN
STORE IN A COOL PLACE.
KEEP TIGHTLY CLOSED WHEN NOT IN USE.

Store between 10°-30°C (50°-86°F).

Rev. 04-14

TAKE TIME **OBSERVE LABEL DIRECTIONS**

3 57561 00458 0

SAFETY DATA SHEET

Manufactured for:
Agri Laboratories, Ltd
(AgriLabs)
St. Joseph, MO 64505
Tel: 816-233-9533

Manufactured by:
Centaur Animal Health
Olathe, KS Tel: 913-390-6184
Emergency Phone: 800-424-9300 (Chemtrec)

AgriLabs Product
Trade Name:
Iodine Wound Spray

Iodine Solution 1%

(Solution of Iodine)

NFPA Rating: Health 2, Flammability 0, Reactivity 0 Special 0
HMIS Rating: Health 2, Flammability 0, Physical 1, Reactivity, 0

1. Product Identity

Product Name: Iodine Tincture, 1%
Product Number: 27

2. Hazardous Ingredients

Iodine complex: Irritant

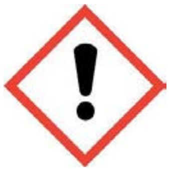

Signal Word: Warning
Hazard Statement: Causes severe eye irritation can cause skin irritation.
Precautionary Statements: Take time observe label directions. If taken internally, gastric disturbances will result. Keep away from children. Keep away from heat & open flame. Keep container closed when not in use. Avoid contact with eyes. Wash skin thoroughly after handling. Antiseptic.

3. Composition

Iodine complex:	Alpha-(p-nonylphenyl) omega hydroxpoly (oxyethylene)-iodine complex<br>CAS: 7553-56-2
Surfactant (NP-12):	CAS: 9016-45-9
Water:	CAS 7732-18-5

Weight%: Iodine Complex: 5%
Water & Surfactant: 95%

4. First Aid Measures	Eye Contact:	Immediately flush eyes with plenty of water for five minutes, then remove contact lens if present. Continue flushing for at least 15 minutes. See medical if irritation persists.
	Inhalation:	Move victim to fresh air if inhaled. Seek medical help if breathing is distressed.
	Ingestion:	Do not induce vomiting. Give victim large quantities of milk or water and seek immediate medical attention. Never give anything by mouth to an unconscious person.
	Skin Contact:	Flush with large quantities of water. Seek medical attention if irritation develops.
5. Fire Fighting Measures	Flash Point:	NE
	Est. Explosive Range Limit	LEL 0.00% UEL 0.00%
	Flash Point Method Used:	N/A
	Extinguishing Media:	Water, carbon dioxide, dry foam.
	Special Fire-Fighting Procedure:	Not applicable
	Unusual Fire/Explosion Hazards:	Flammable, do not exposure to heat, sparks, open flames or other ignition sources. Emits toxic iodine fumes with exposure to high heat.
6. Accidental Release Measures	<u>Steps to take in case of spill, leak or release:</u><br><br>Dilute with water. Neutralize iodine with sodium metabisulfite or sodium thiosulfate with soda ash. Use clay absorbent.	
7. Handling & Storage	<u>Precautions to take in handling and storing:</u>	

	Store away from heat or open flame. Avoid storage near chlorinated products.<br><br><u>Other precautions:</u><br><br>*Thoroughly rinse all measuring devices, and store out of reach of children.*<br><br>Keep container tightly closed when not using.<br><br>Avoid contamination of food or feed.
8. Control Measures	OSHA PEL: NE<br>ACGIH TLV: NE<br><br><u>Ventilation:</u><br><br>Adequate ventilation is required. Avoid breathing vapors.<br><br><u>Protective gloves:</u><br><br>Protective gloves are recommended.<br><br><u>Eye protection:</u><br><br>Recommended.<br><br><u>Other protective clothing or equipment:</u><br><br>Not required.<br><br><u>Work or Hygienic Practices:</u><br><br>Use good personal hygiene when handling this product. Wash hands after use, before smoking or eating, and before using the toilet.
9. Physical Properties	Appearance: Dark brown liquid<br>Odor: Characteristic iodine<br>Specific Gravity: 1.012 @ 25 C°<br>pH: 4.5 – 5.2<br>Boiling point: Not applicable

	Vapor Pressure:	Not applicable
	Evaporation Rate:	Not applicable
	Solubility in Water:	Complete
10. Stability & Reactivity	Stability:	Stable at room temperature
	Incompatibility:	Chlorinated products, strong detergents.
	Hazardous decomposition or by-products:	Emits iodine vapors with high temperature.
	Hazardous polymerization:	Will not occur.
	Conditions to avoid:	High temperatures, mixing with chlorinated products.
11. Toxicological Information	<u>Acute Health Effects</u>	
	Eye Contact:	Will cause moderate to severe irritation.
	Inhalation:	Irritating to nose, throat and respiratory tract.
	Ingestion:	Harmful if swallowed. Swallowing will cause irritation to mucous membranes.
	Skin Contact:	None.
	Signs and symptoms: of over exposure	Redness of eyes, burning mucous membrane, upset stomach.
	Aggravated medical conditions:	None
	Supplemental health information:	Persons with a known allergy to shellfish and or iodine should never use this product, or be in contact with this product.
12. Ecological Information	No additional information is available at this time.	

13. Disposal Information	<u>Waste disposal method:</u><br><br>Follow all local, state and federal regulations.
14. Transport Information	DOT Class: Not hazardous<br>Hazard Class: N/A<br>UN Number: N/A<br>Package code: N/A<br>Shipping Name: Not regulated.
15. Regulatory Information	***This material is exempt from DEA regulation.***<br><br>*The information contained herein is based on the data available to us and is believed to be accurate. However, no warranty is expressed or implied regarding the accuracy of this data or the results to be obtained from the use thereof. We assume no responsibility for injuries from the use of the product described herein.*
16. Additional Information	Date Created: 07/22/2014

DISCLAIMER:
Agri Laboratories, Ltd (AgriLabs) provides the information contained in this SDS sheet in good faith but makes no claim as to its accuracy or comprehensiveness. This SDS sheet is intended as a guide for a properly trained person to utilize appropriate precautionary methods while handling this product. Individuals must exercise their own judgment in determining the appropriateness of this information for a particular purpose. AgriLabs will not be responsible for damages resulting from the use or misuse of the information contained in this Safety Data Sheet.
