# Supplementary material for: Topical wound-care products and their effects on healing, inflammatory biomarkers, and growth in piglets undergoing castration
Source: Porcine Health Manag. 2026 Apr 21;12:23. doi: 10.1186/s40813-026-00492-7 (PMC13097753; doi:10.1186/s40813-026-00492-7)
Supplement: Supplementary file 2 — Supplementary Material 2 [file 40813_2026_492_MOESM2_ESM.pdf]

Rugby Zinc Oxide

Drug Facts

Active ingredient

Zinc oxide 20%

Purpose

Skin Protectant

Uses

- helps treat and prevent diaper rash. Protects chafed skin due to diaper rash and helps seal out wetness.
- dries the oozing and weeping of poison ivy, poison oak and poison sumac.

Warnings

For external use only

When using this product,avoid contact with the eyes

Stop use and ask a doctor ifcondition worsens or does not improve within 7 days

Keep out of reach of children. If swallowed, get medical help or contact a poison control center right away.

Directions

- apply liberally as often as necessary

For diaper rash:

- change wet or soiled diapers promptly
- cleanse the diaper area and allow to dry
- apply ointment liberally, as often as necessary, with each diaper change and especially at bedtime or anytime when exposure to wet diapers may be prolonged

Inactive ingredients

mineral oil, petrolatum

Questions or comments?

Call **1-800-645-2158**9 am - 5 pm EST, Monday – Friday

Store at room temperature 15° - 30°C (59° - 86°F).

R0507

Principal Display Panel – Jar Label

Rugby®NDC 0536-5700-98
SKIN PROTECTANT

Zinc Oxide
Ointment USP
• provides soothing relief from poison ivy,
oak and sumac
• Treats and prevents diaper rash

Net Wt 1 lb (454 g)

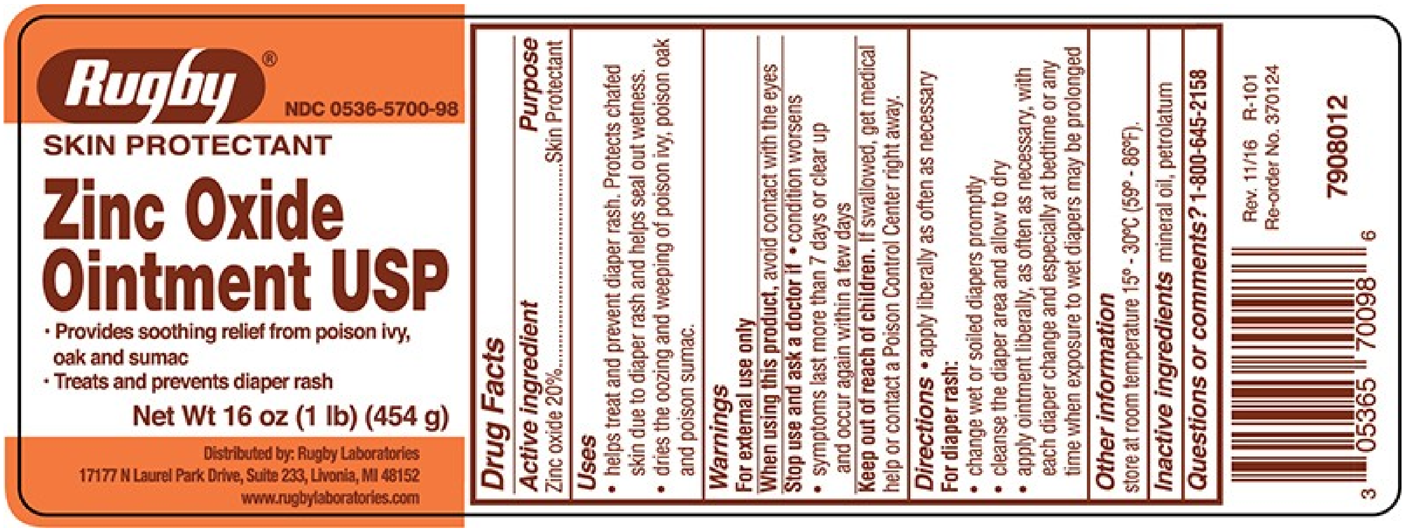

Principal Display Panel - 30 g Carton

Rugby Zinc Oxide Ointment USP

Skin protectant Zinc Oxide 20%

Help Soothe & Protect Chafed Skin

NET WT 30G (1.05oz)

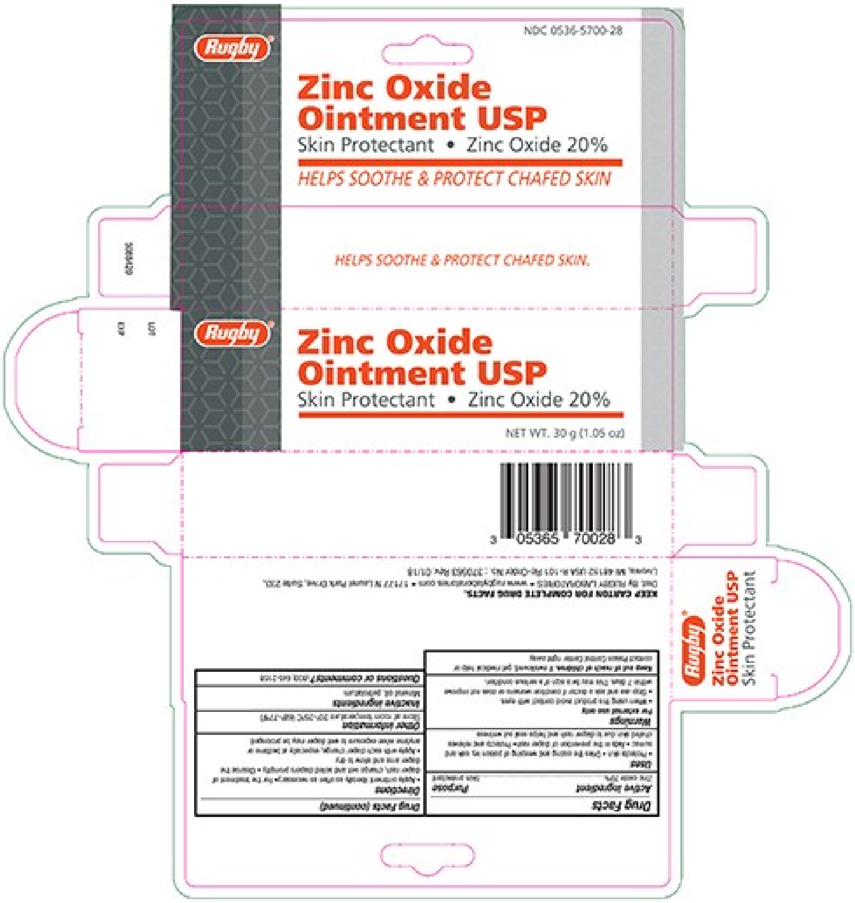

Principal Display Panel - 30 g Tube

Rugby Zinc Oxide Ointment USP

Skin protectant Zinc Oxide 20%

Help Soothe & Protect Chafed Skin

NET WT 30G (1.05oz)

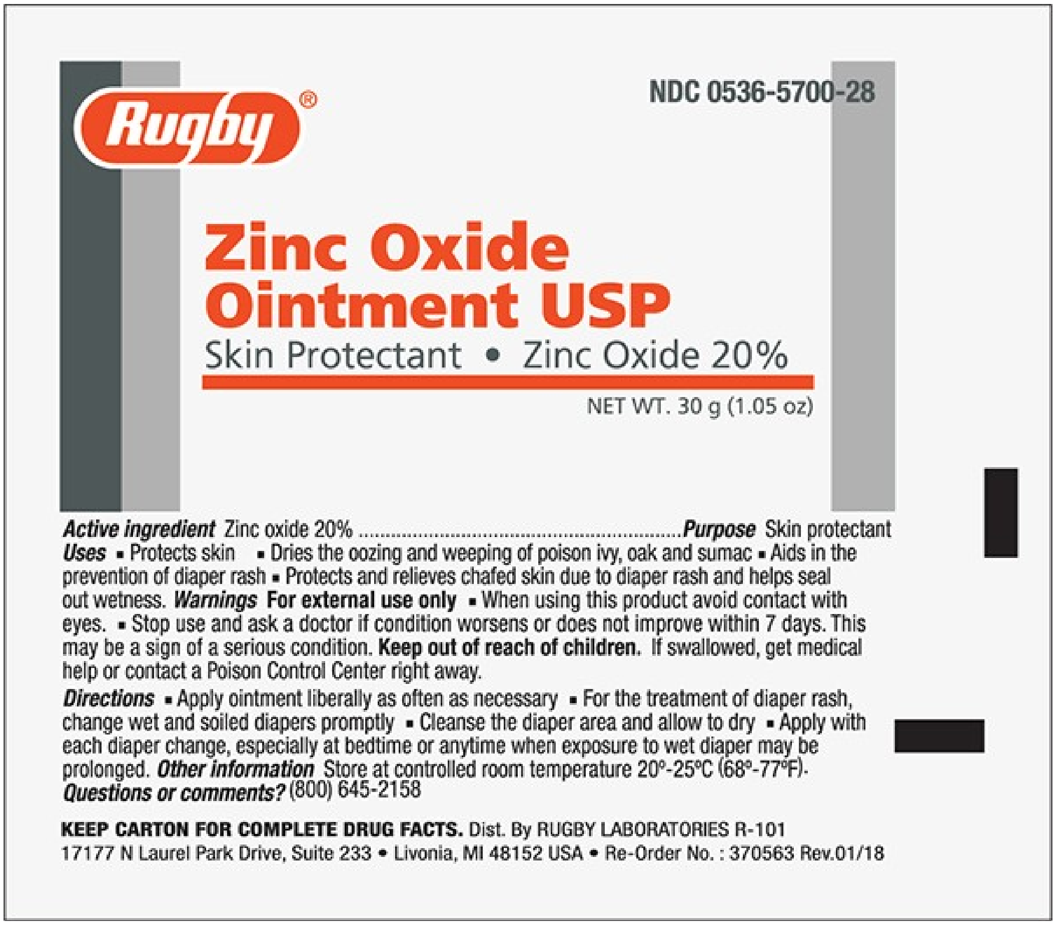

RUGBY ZINC OXIDE

zinc oxide ointment

Product Information

**Product Type**	HUMAN OTC DRUG	**Item Code (Source)**	NDC:0536-5700
**Route of Administration**	TOPICAL		

Active Ingredient/Active Moiety

Ingredient Name	Basis of Strength	Strength
**ZINC OXIDE** (UNII: SOI2LOH54Z) (ZINC OXIDE - UNII:SOI2LOH54Z)	ZINC OXIDE	200 mg in 1 g

Inactive Ingredients

Ingredient Name	Strength
**MINERAL OIL** (UNII: T5L8T28FGP)	
**PETROLATUM** (UNII: 4T6H12BN9U)	

Packaging

#	Item Code	Package Description	Marketing Start Date	Marketing End Date
1	NDC:0536-5700-98	454 g in 1 JAR; Type 0: Not a Combination Product	03/23/2004	03/31/2024

**2**	NDC:0536-5700-28	1 in 1 CARTON	09/24/2018	03/31/2024
**2**		30 g in 1 TUBE; Type 0: Not a Combination Product		
**3**	NDC:0536-5700-25	1 in 1 CARTON	09/06/2018	03/31/2024
**3**		60 g in 1 TUBE; Type 0: Not a Combination Product		

Marketing Information

Marketing Category	Application Number or Monograph Citation	Marketing Start Date	Marketing End Date
OTC Monograph Drug	M016	03/23/2004	03/31/2024

Labeler - Rugby Laboratories (079246066)

Revised: 1/2026 Rugby Laboratories

SAFETY DATA SHEET

Prepared to U.S. OSHA, CMA, ANSI, Canadian WHMIS Standards, REACH, European Union CLP EC 1272/2008 and the Global Harmonization Standard

PART I *What is the material and what do I need to know in an emergency?*

1. SECTION 1 – IDENTIFICATION OF THE SUBSTANCE/MIXTURE AND OF THE COMPANY/UNDERTAKING

TRADE NAME/MATERIAL NAME: Zinc Oxide Ointment

DESCRIPTION:	Zinc Oxide Ointment 20%
NDC #:	00168-0062-02, 0168-0062-16, 0168-0062-31
CHEMICAL NAME (for active ingredient):	Zinc Oxide
CHEMICAL FAMILY:	Metal Oxide
HOW SUPPLIED:	20% Topical Zinc Oxide Ointment
FORMULA (for active ingredient):	ZnO
RELEVANT USE of the SUBSTANCE:	Pharmaceutical for Human Use
USES ADVISED AGAINST	Other than Relevant Use
SUPPLIER/MANUFACTURER'S NAME:	**FOUGERA PHARMACEUTICALS INC.**
ADDRESS:	60 Baylis Road<br>Melville, NY 11747
BUSINESS PHONE/GENERAL SDS INFORMATION:	1-631-454-7677
EMERGENCY PHONE (U.S./Canada/Puerto Rico):	CHEMTEL: (U.S., Canada) 1(800)255-3924 (24 hrs)<br>(International) +1 813 248 0585 (24 hrs)

ALL WHMIS required information is included in appropriate sections based on the ANSI Z400.1-2010 format. This material has been classified in accordance with the hazard criteria of the CPR and the SDS contains all the information required by the CPR. The material is also classified per all applicable EU Directives through EC 1907: 2006, the European Union CLP EC 1272/2008 and the Global Harmonization Standard.

2. HAZARD IDENTIFICATION

GLOBAL HARMONIZATION AND EU CLP REGULATION (EC) 1272/2008 LABELING AND CLASSIFICATION: According to Article 1, item 5 (a) of CLP Regulation (EC) 1272/2008, medicinal products in the finished state for human use, as defined in 2001/83/EC, are excepted from classification and other criteria of 1272/2008.

EU LABELING/CLASSIFICATION: According to Article 1 of European Union Council Directive 92/32/EEC, medical products in the finished state for human use (as defined by European Union Council Directives 67/548/EEC and 87/21/EEC) are not subject to the regulations and administrative provisions of European Union Council Directive 92/32/EEC.

EMERGENCY OVERVIEW: Product Description: This product is a white ointment with a petroleum jelly odor. ***Health Hazards:*** May be harmful if swallowed. Eye contact may cause irritation. ***Flammability Hazards:*** If heated to high temperatures for a prolonged period, this product may ignite. When involved in a fire, this material may decompose and produce irritating vapors and toxic compounds (including carbon, zinc and nitrogen oxides). ***Reactivity Hazards:*** This product is not reactive. ***Environmental Hazards:*** This product has not been tested for environmental effects; however the Zinc Oxide component is acutely and chronically toxic to aquatic organisms. All release to the environment should be avoided. ***Emergency Considerations:*** Emergency responders should wear appropriate protection for situation to which they respond.

3. COMPOSITION and INFORMATION ON INGREDIENTS

CHEMICAL NAME	CAS #	EINECS #	% w/w	**LABEL ELEMENTS**<br>**EU Classification (67/548/EEC)**<br>**GHS & EU Classification (1272/2008 EC)**<br>**Risk Phrases/Hazard Statements**
ACTIVE INGREDIENT				
Zinc Oxide	1314-13-2	215-222-5	20.0%	PUBLISHED and SELF-CLASSIFICATION<br>EU 67/548<br>Classification: Dangerous for the Environment<br>Risk Phrase Codes: R50/53<br>Hazard Symbols: N<br>GHS and EU 1272/2008<br>Classification: Acute Inhalation Toxicity Cat. 5, Aquatic Acute Toxicity Cat. 1, Aquatic Chronic Toxicity Cat. 1<br>Hazard Codes: H333, H400, H410<br>Hazard Symbol/Pictogram: GHS09
Beeswax	8012-89-3	232-383-7	Proprietary	EU 67/548: CLASSIFICATION: NOT APPLICABLE.<br>GHS & EU 1272/2008: CLASSIFICATION: NOT APPLICABLE.
Mineral Oil	8042-47-5	232-455-8	Proprietary	SELF CLASSIFICATION<br>EU 67/548<br>Classification: Carcinogenic Cat. 3<br>Risk Phrases: R45<br>Hazard Symbol: Xn<br>EU/GHS 1272/2008<br>Classification: Carcinogenic Cat. 2, Eye Irritation Cat. 2B<br>Hazard Statement Codes: H351, H320<br>Hazard Symbol/Pictogram: GHS08

See Section 16 for full classification information of product and components.

3. COMPOSITION and INFORMATION ON INGREDIENTS (Continued)

CHEMICAL NAME	CAS #	EINECS #	% w/w	**LABEL ELEMENTS**<br>**EU Classification (67/548/EEC)**<br>**GHS & EU Classification (1272/2008 EC)**<br>**Risk Phrases/Hazard Statements**
EXCIPIENTS (continued)				
White Petrolatum	8009-03-8	232-373-2	Proprietary	<u>EU 67/548</u><br>Classification: Carcinogenic Cat. 2<br>Risk Phrase Codes: R45<br>Hazard Symbols: Xn<br><u>GHS and EU 1272/2008</u><br>Classification: Carcinogenic Cat. 1B<br>Hazard Codes: H350<br>Hazard Symbol/Pictogram: GHS08

See Section 16 for full classification information of product and components.

PART II *What should I do if a hazardous situation occurs?*

4 FIRST-AID MEASURES

PROTECTION OF FIRST AID RESPONDERS: rescuers should wear adequate personal protective equipment. Rescuers should be taken for medical attention, if necessary.

DESCRIPTION OF FIRST AID MEASURES: Contaminated individuals must be taken for medical attention if any adverse effects occur. Persons developing hypersensitivity reactions should receive medical attention. If breathing is difficult, give oxygen. If not breathing, give artificial respiration. Only trained personnel should administer supplemental oxygen and/or cardio-pulmonary resuscitation, if necessary. Remove victim(s) to fresh air, as quickly as possible. Take copy of product label and SDS to physician or other health professional with victim(s).

Skin Exposure: If adverse skin effects occur, discontinue use. Seek medical attention.

Eye Exposure: If this product contaminates the eyes, rinse eyes under gently running water. Use sufficient force to open eyelids and then "roll" eyes while flushing. Minimum flushing is for 20 minutes. The contaminated individual must seek medical attention if any adverse effect continues after rinsing.

Inhalation: If vapors of this product are inhaled, causing irritation, remove victim to fresh air. If necessary, use artificial respiration to support vital functions.

Ingestion: If this product is swallowed, CALL PHYSICIAN OR POISON CONTROL CENTER FOR MOST CURRENT INFORMATION. If professional advice is not available, do not induce vomiting. Never induce vomiting or give diluents (milk or water) to someone who is <u>unconscious, having convulsions, or unable to swallow</u>. If victim is convulsing, maintain an open airway and obtain immediate medical attention.

IMPORTANT SYMPTOMS AND EFFECTS: See Sections 2 (Hazard Identification) and 11 (Toxicological Information).

MEDICAL CONDITIONS AGGRAVATED BY EXPOSURE: Pre-existing skin disorders may be aggravated. Workplace exposure may also aggravate these conditions. Persons who may have hypersensitivity reactions to component, or other disorders described in Section 11 (Toxicological Information) may experience aggravation upon exposure.

INDICATION OF IMMEDIATE MEDICAL ATTENTION AND SPECIAL TREATMENT IF NEEDED: Treat symptoms and eliminate exposure. Persons developing hypersensitivity reactions should receive medical attention. No specific antidote is known. Treatment should be symptomatic and supportive.

5. FIRE-FIGHTING MEASURES

FLASH POINT: Not available.

AUTOIGNITION TEMPERATURE: Not available.

FLAMMABLE LIMITS (in air by volume, %): Not applicable.

FIRE EXTINGUISHING MEDIA: Use extinguishing media appropriate for surrounding fire.

UNSUITABLE FIRE EXTINGUISHING MEDIA: None known.

SPECIAL HAZARDS ARISING FROM THE PRODUCT: If heated to high temperatures for a prolonged period this product can ignite. When involved in a fire, this material may decompose and produce irritating vapors and toxic compounds (including carbon, zinc and nitrogen oxides).

Explosion Sensitivity to Mechanical Impact or Static Discharge: Not sensitive.

SPECIAL PROTECTIVE ACTIONS FOR FIRE-FIGHTERS: Incipient fire responders should wear eye protection. Structural firefighters must wear Self-Contained Breathing Apparatus (SCBA) and full protective equipment. If protective equipment is contaminated by this product, it should be thoroughly washed with running water prior to removal of SCBA respiratory protection. Firefighters whose protective equipment becomes contaminated should thoroughly shower with warm, soapy water and should receive medical evaluation if they experience any adverse effects.

NFPA RATING

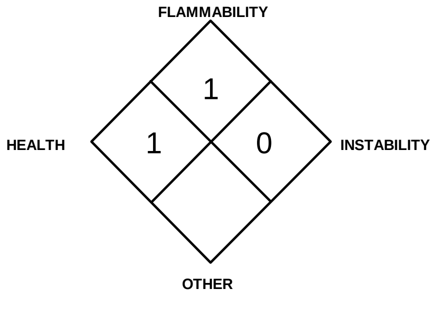

Hazard Scale: **0** = Minimal **1** = Slight **2** = Moderate **3** = Serious **4** = Severe

6. ACCIDENTAL RELEASE MEASURES

PERSONAL PRECAUTIONS, PROTECTIVE EQUIPMENT AND EMERGENCY PROCEDURES: Spill kits, clearly labeled, should be kept in or near preparation and administrative areas. It is suggested that kits include a respirator, chemical splash goggles, two pairs of gloves, two sheets (12” x 12”) of absorbent material, 250-mL and 1-liter spill control pillows and a small scoop to collect glass fragments (if applicable). Absorbents should be incinerable. Finally, the kit should contain two large waste-disposal bags. Avoid generating aerosols from this product. Spills may be slippery.

PROTECTIVE EQUIPMENT:

Small Spills: Wear goggles and gloves while wiping up small spills of this product with polypad or sponge.

Large Spills: Use proper protective equipment, including double nitrile or appropriate gloves, full body gown, and full-face respirator equipped with a High Efficiency Particulate (HEPA) filter. Self-Contained Breathing Apparatus (SCBA) can be used instead of an air-purifying respirator.

METHODS FOR CLEAN-UP AND CONTAINMENT:

Cleanup of Small Spills: The product should be gently covered with absorbent pads. Clean spill with pad and dispose of properly. Decontaminate the spill area (three times) using a bleach and detergent solution and then rinse with clean water.

Large Spills: Review Sections 2, 8, 11 and 12 before proceeding with cleanup. Restrict access to the spill areas. For spills of amounts larger than 5 mL limit spread by gently covering with absorbent sheets, or spill-control pads or pillows. Be sure not to generate aerosols. The dispersion of aerosols into surrounding air and the possibility of inhalation is a serious matter and should be treated as such. Do not apply chemical in-activators as they may produce hazardous by-products. Thoroughly clean all contaminated surfaces three times using a bleach and detergent solution and then rinse with clean water.

All Spills: Use procedures described above and then place all spill residues in an appropriate, labeled container and seal. Move to a secure area. Dispose of in accordance with Federal, State, and local hazardous waste disposal regulations (see Section 13, Disposal Considerations). For spills on water, contain, minimize dispersion and collect. Dispose of recovered product and report spill per regulatory requirements.

ENVIRONMENTAL PRECAUTIONS: Prevent product from entering sewer or confined spaces, waterways, soil or public waters. Do not flush to sewer. For spills on water, contain, minimize dispersion and collect.

REFERENCE TO OTHER SECTIONS: Review Sections 2, 8, 11 and 12 before proceeding with cleanup. See Section 13, Disposal Considerations for more information.

PART III *How can I prevent hazardous situations from occurring?*

7. HANDLING and USE

PRECAUTIONS FOR SAFE HANDLING: All employees who handle this product should be thoroughly trained to handle it safely. As with all chemicals, avoid getting this product ON YOU or IN YOU. Do not eat or drink while handling this product. Appropriate personal protective equipment must be worn (see Section 8, Engineering Controls and Personal Protection). Avoid generation of aerosols.

PRODUCT PREPARATION INSTRUCTIONS FOR MEDICAL PERSONNEL: Handle this material following standard medical practices and following the recommendations presented on the Package Insert.

CONDITIONS FOR SAFE STORAGE: Containers of this product must be properly labeled. Store containers in a cool, dry location, away from direct sunlight and sources of intense heat. Recommended Storage Temperature: 20-25°C (68-77°F) [USP Controlled Room Temperature]. Protect from freezing. Store away from incompatible materials (see Section 10, Stability and Reactivity). Product should be stored in secondary containers. Keep containers tightly closed when not in use. Inspect all incoming containers before storage, to ensure containers are properly labeled and not damaged. Have appropriate extinguishing equipment in the storage area (e.g., sprinkler system, portable fire extinguishers). Empty containers may contain residual product; therefore, empty containers should be handled with care and disposed of properly.

SPECIFIC END USE(S): This product is a human pharmaceutical.

PROTECTIVE PRACTICES DURING MAINTENANCE OF CONTAMINATED EQUIPMENT: When cleaning non-disposable equipment, wear nitrile or other appropriate gloves (double gloving is recommended), goggles, and lab coat. Wipe equipment down with damp sponge or polypad. If applicable, wash equipment using a bleach and detergent solution and then rinse with clean water. Collect all rinsates and dispose of according to applicable waste disposal regulations or waste disposal regulations of Canada. All disposable items contaminated with this product should be disposed of properly.

8. EXPOSURE CONTROLS - PERSONAL PROTECTION

EXPOSURE LIMITS/CONTROL PARAMETERS:

Ventilation and Engineering Controls: Use with adequate ventilation. Follow standard medical product handling procedures. During decontamination of work surfaces, workers should wear the same equipment recommended in Section 6 (Accidental Release Measures) of this SDS.

8. EXPOSURE CONTROLS - PERSONAL PROTECTION (Continued)

EXPOSURE LIMITS/CONTROL PARAMETERS (continued):

Workplace Exposure Limits/Control Parameters:

CHEMICAL NAME	CAS #	EXPOSURE LIMITS IN AIR							
		ACGIH-TLVs		OSHA-PELs		NIOSH-RELs		NIOSH	OTHER
		TWA mg/m^3	STEL mg/m^3	TWA mg/m^3	STEL mg/m^3	TWA mg/m^3	STEL mg/m^3	IDLH mg/m^3	mg/m^3
Zinc Oxide	1314-13-2	2 (resp. fract.)	10 (resp. fract.)	15 (total dust), 5 (resp. fract.) Vacated 1989 PEL: 10 (total dust)	NE	5 (dust only)	15 (ceiling)	500	DFG MAKs: TWA = 0.1 (respiratory fraction), 2 (inhalable fraction) PEAK = 4•MAK 15 min. average value, 1-hr interval, 4 per shift (respirable fraction); 2•MAK 15 min. average value, 1-hr interval, 4 per shift (inhalable fraction) Carcinogen: EPA-II, EPA-D
Beeswax	8012-89-3	NE	NE	NE	NE	NE	NE	NE	NE
Mineral Oil Exposure limits are for oil mist, mineral	8042-47-5	5 (inhal. fract.)	NE	NE	NE	NE	NE	NE	Carcinogen: IARC-3, TLV-A4
White Petrolatum	8009-03-8	NE	NE	NE	NE	NE	NE	NE	NE

NE = Not Established See Section 16 for Definitions of Terms Used.

International Occupational Exposure Limits: Exposure limits available for some excipient components are given below.

MINERAL OIL:
Australia: TWA = 5 mg/m^3, JUL 2008
Belgium: = TWA 5 mg/m^3, STEL = 10 mg/m^3, MAR 2002
Denmark: TWA = 1 mg/m^3, MAY 2011
Hungary: CL = 5 mg/m^3, Carcinogen, SEP 2000
Japan: OEL = 3 mg/m^3 (mist), 1 carc, MAY 2012
Korea: TWA = 5 mg/m^3, STEL = 10 mg/m^3, 2006
Mexico: TWA = 5 mg/m^3; STEL = 10 mg/m^3, 2004
The Netherlands: MAC-TGG = 5 mg/m^3, 2003
New Zealand: TWA = 5 mg/m^3, STEL 10 ppm, JAN 2002
The Philippines: TWA = 5 mg/m^3, JAN 1993
Poland: MAC(TWA) = 5 mg/m^3, MAC(STEL) = 10 mg/m^3, JAN 1999
Russia: STEL = 5 mg/m^3, JUN 2003
Sweden: TWA = 1 mg/m^3; STEL = 3 mg/m^3, JUN 2005
In Argentina, Bulgaria, Colombia, Jordan, Korea, New Zealand, Singapore, Vietnam, New Zealand, Singapore, Vietnam check ACGIH TLV

ZINC OXIDE:
Arab Republic of Egypt: TWA = 5 mg/m^3, JAN 1993
Australia: TWA = 10 mg/m^3 (dust), JUL 2008
Australia: TWA = 5 mg/m^3, STEL = 10 mg/m^3 (fume), JUL 2008
Austria: MAK-TMW = 5 mg/m^3, resp, 2007
Belgium: TWA = 10 mg/m^3 (dust), MAR 2002
Belgium: TWA = 5 mg/m^3, STEL = 10 mg/m^3 (fume), MAR 2002
Denmark: TWA = 4 mg(Zn)/m^3 (fume), MAY 2011
Finland: TWA = 2 mg/m^3, STEL = 10 mg/m^3, NOV 2011
France: VME 10 mg/m^3 (dust), 5 mg/m^3 (fume), FEB 2006

ZINC OXIDE (continued):
Germany: MAK 0.1 mg(Zn)/m^3, resp, 2011
Germany: MAK 2 mg(Zn)/m^3, inhal, 2011
Hungary: TWA = 5 mg/m^3 (resp), STEL = 20 mg/m^3 (resp), SEP 2000
Iceland: TWA 4 mg(Zn)/m^3, NOV 2011
Japan: OEL = 1 mg/m^3 (resp. dust), 4 mg/m^3 (total dust), MAY 2012
Korea: TWA = 10 mg/m^3, 2006
Korea: TWA = 5 mg/m^3, STEL = 10 mg/m^3, 2006
Mexico: TWA = 5 mg/m^3 (fume), 2004
Mexico: TWA = 10 mg/m^3; STEL = 10 mg/m^3 (inhalable), 2004
The Netherlands: MAC-TGG = 5 mg/m^3, 2003
New Zealand: TWA = 5 mg/m^3 (fume), STEL = 10 ppm (fume), JAN 2002
New Zealand: TWA = 10 mg/m^3 (inspirable dust), JAN 2002
Norway: TWA = 5 mg/m^3, JAN 1999
Peru: TWA = 2 mg/m^3; STEL = 10 mg/m^3, JUL 2005
The Philippines: TWA = 1 mg/m^3, JAN 1993
Poland: MAC(TWA) fume = 5 mg/m^3, MAC(STEL) = fume 10 mg/m^3, JAN 1999
Russia: TWA = 0.5 mg/m^3, STEL = 1.5 mg/m^3, JUN 2003
Sweden: TWA = 5 mg/m^3, JUN 2005
Switzerland: CL = 0.125 mg/m^3, fume, JAN 2011
Switzerland: MAK-W = 0.1 mg(Zn)/m^3, KZG-W = 0.4 mg(Zn)/m^3, resp, JAN 2011
Switzerland: MAK-W = 2 mg(Zn)/m^3, KZG-W = 4 mg(Zn)/m^3, inhal, JAN 2011
Thailand: TWA = 5 mg/m^3 (fume), JAN1993
Turkey: TWA = 5 mg/m^3, JAN 1993
In Argentina, Bulgaria, Colombia, Jordan, Korea, New Zealand, Singapore, Vietnam check ACGIH TLV

PROTECTIVE EQUIPMENT: *The following information on appropriate Personal Protective Equipment is provided to assist employers in complying with OSHA regulations found in 29 CFR Subpart I (beginning at 1910.132, including* U.S. Federal OSHA Respiratory Protection (29 CFR 1910.134), OSHA Eye Protection 29 CFR 1910.133, OSHA Hand Protection 29 CFR 1910.138, OSHA Foot Protection 29 CFR 1910.136 and OSHA Body Protection 29 CFR1910.132)*, equivalent standards of Canada (including CSA Respiratory Standard Z94.4-02,* Z94.3-M1982, *Industrial Eye and Face Protectors and* CSA Standard Z195-02, *Protective Footwear), or standards of EU member states (including EN 529:2005 for respiratory PPE, CEN/TR 15419:2006 for hand protection, and CR 13464:1999 for face/eye protection). Please reference applicable regulations and standards for relevant details.*

Respiratory Protection: Maintain airborne contaminant concentrations below exposure limits listed above, if applicable. For materials without listed exposure limits, minimize respiratory exposure. If necessary, use only respiratory protection authorized under appropriate regulations. Oxygen levels below 19.5% are considered IDLH by U.S. OSHA. In such atmospheres, use of a full-facepiece pressure/demand SCBA or a full facepiece, supplied air respirator with auxiliary self-contained air supply is required under U.S. OSHA's Respiratory Protection Standard (1910.134-1998).

Eye Protection: Wear splash goggles or safety glasses as appropriate for the task. If necessary, refer to appropriate regulations.

Skin Protection: Use appropriate protective clothing for the task (e.g., lab coat, etc.). If necessary, refer to the U.S. OSHA Technical Manual (Section VII: Personal Protective Equipment) or other appropriate regulations.

Hand Protection: Wash hands and wrists before putting on and after removing gloves. During manufacture or other similar industrial operations, wear the appropriate hand protection for the process. When used in medical administration of the product, double glove with nitrile or other appropriate gloves to avoid contact and/or absorption of the product. Use double gloves for spill response, as stated in Section 6 (Accidental Release Measures) of this SDS. Because all gloves are to some extent permeable and their permeability increases with time, they should be changed regularly (hourly is preferable) or immediately if torn or punctured. If necessary refer to appropriate regulations.

9. PHYSICAL and CHEMICAL PROPERTIES

FORM: Ointment.
COLOR: White.
MOLECULAR WEIGHT: Mixture.
MOLECULAR FORMULA: Mixture.

9. PHYSICAL and CHEMICAL PROPERTIES (Continued)

ODOR: Petroleum jelly odor.
BOILING POINT: > 121°C (250°F)
EVAPORATION RATE (nBuAc = 1): Not established.
VAPOR PRESSURE (air = 1): Not established.
SOLUBILITY IN WATER: Insoluble.
ODOR THRESHOLD: Not established.
FREEZING/MELTING POINT: Not available.
pH: Not established.
SPECIFIC GRAVITY @20°C (water = 1): 1.01
OTHER SOLUBILITIES: Not known.
COEFFICIENT WATER/OIL DISTRIBUTION: Not established.
HOW TO DETECT THIS SUBSTANCE (warning properties): The appearance of this product can be a distinguishing characteristic to identify it in event of accidental release.

10. STABILITY and REACTIVITY

CHEMICAL STABILITY: This product is stable.
DECOMPOSITION PRODUCTS: ***Combustion:*** If exposed to extremely high temperatures, thermal decomposition may generate irritating fumes and toxic gases (e.g., carbon, zinc and nitrogen oxides). ***Hydrolysis:*** None known.
MATERIALS WITH WHICH SUBSTANCE IS INCOMPATIBLE: This product is generally compatible with other common materials in a medical facility. Acids, caustics, and other chemicals that could affect its performance should be avoided.
POSSIBILITY OF HAZARDOUS REACTIONS/POLYMERIZATION: Will not occur.
CONDITIONS TO AVOID: Avoid heat, light, and contact with incompatible chemicals.

PART IV *Is there any other useful information about this material?*

11. TOXICOLOGICAL INFORMATION

SYMPTOMS OF EXPOSURE BY ROUTE OF EXPOSURE: The health hazard information provided below is pertinent to medical employees handling this product in an occupational setting. This product is designed for application on the skin. The following paragraphs describe the symptoms of exposure by route of exposure.

Inhalation: Although unlikely, due to high viscosity of the product, inhalation of mists or sprays of this product, especially in a poorly ventilated space, may cause irritation, coughing, and sneezing.

Contact with Skin or Eyes: Skin contact is not expected to cause adverse effects. Eye contact can cause irritation, stinging, redness, and tearing.

Skin Absorption: Zinc Oxide can be absorbed into the body through intact skin, but this route of exposure is not expected to produce harmful effects. The amount absorbed depends on a number of factors including the concentration of zinc already in the bloodstream, the pH of the skin, and the amount of Zinc Oxide applied.

Ingestion: Ingestion is not a significant route of occupational exposure. Acute ingestion of large quantities of this product or chronic ingestion caused by poor hygiene practices may cause nausea, vomiting, and diarrhea. Ingestion of large amount may lead to zinc toxicity. The most common symptoms of zinc toxicity include nausea, vomiting, abdominal pain, diarrhea and, in severe cases, vomiting of blood.

Injection: Though not anticipated to be a significant route of exposure for this product, injection (via punctures or lacerations by contaminated objects) may cause redness at the site of injection.

HAZARDOUS MATERIAL IDENTIFICATION SYSTEM

**HEALTH HAZARD**	(BLUE)	1
**FLAMMABILITY HAZARD**	(RED)	1
**PHYSICAL HAZARD**	(YELLOW)	0

PROTECTIVE EQUIPMENT

EYES	RESPIRATORY	HANDS	BODY
	SEE SECTION 8		SEE SECTION 8

For Routine Industrial Use and Handling Applications

Hazard Scale: **0** = Minimal **1** = Slight **2** = Moderate **3** = Serious **4** = Severe * = Chronic hazard

GENERAL TOXICITY INFORMATION: Persons using the product in therapeutic doses may experience temporary blurred vision and mild irritation.
IRRITANCY OF PRODUCT: This product may irritate the eyes. Skin contact is not expected to cause irritation.
SENSITIZATION OF PRODUCT: No information is available on sensitization effects.
HEALTH EFFECTS OR RISKS FROM EXPOSURE: An Explanation in Lay Terms. Exposure to this product may cause the following health effects:

Acute: Ingestion may be harmful. Eye contact may cause irritation.
Chronic: None known.

TARGET ORGANS:

Acute: *Occupational Exposure:* Skin, eyes. *Therapeutic Doses:* Skin.
Chronic: *Occupational Exposure:* None known. *Therapeutic Doses:* None known.

TOXICITY DATA: Only toxicity data available for the active component of this product are presented in this SDS. Additional data are available for the excipient components of this product, but are not presented; Contact Fougera for more information.

ZINC OXIDE:
Standard Draize Test (Skin-Rabbit) 500 mg/24 hours: Mild
Standard Draize Test (Eye-Rabbit) 500 mg/24 hours: Mild

ZINC OXIDE (continued):
LDLo (Oral-Human) 500 mg/kg
TCLo (Inhalation-Human) 600 mg/m^3: Lungs, Thorax, or Respiration: cough, dyspnea, other changes

11. TOXICOLOGICAL INFORMATION (Continued)

TOXICITY DATA (continued):

ZINC OXIDE (continued):
LD_{50} (Oral-Mouse) 7950 mg/kg
LD_{50} (Intraperitoneal-Rat) 240 mg/kg
LC_{50} (Inhalation-Mouse) 2500 mg/m^3
LD (Oral-Rat) > 8437 mg/kg
LD (Intratracheal-Rat) > 4979 µg/kg: Lungs, Thorax, or Respiration: other changes; Biochemical: Enzyme inhibition, induction, or change in blood or tissue levels: dehydrogenases, Enzyme inhibition, induction, or change in blood or tissue levels: other Enzymes
TCLo (Inhalation-Rat) 25 mg/m^3/3 hours: Lungs, Thorax, or Respiration: other changes; Biochemical: Enzyme inhibition, induction, or change in blood or tissue levels: phosphatases, Metabolism (Intermediary): effect on inflammation or mediation of inflammation
TCLo (Inhalation-Rat) 25 mg/m^3/3 hours: Lungs, Thorax, or Respiration: other changes; Biochemical: Metabolism (Intermediary): effect on inflammation or mediation of inflammation
TCLo (Inhalation-Rat) 6.9 mg/m^3/7 days-intermittent: Lungs, Thorax, or Respiration: other changes, changes in lung weight; Biochemical: Enzyme inhibition, induction, or change in blood or tissue levels: multiple enzyme effects
TCLo (Inhalation-Guinea Pig) 5900 µg/m^3/3 hours/3 days-intermittent: Lungs, Thorax, or Respiration: other changes; Biochemical: Enzyme inhibition, induction, or change in blood or tissue levels: phosphatases, Enzyme inhibition, induction, or change in blood or tissue levels: dehydrogenases
TCLo (Inhalation-Guinea Pig) 4600 µg/m^3/3 hours/6 days-intermittent: Lungs, Thorax, or Respiration: other changes, changes in lung weight
TCLo (Inhalation-Mammal-Species Unspecified) 15 mg/m^3/1 hour/84 days-intermittent: Lungs, Thorax, or Respiration: dyspnea, other changes

ZINC OXIDE (continued):
TCLo (Inhalation-Guinea Pig) 5 mg/m^3/6 days-intermittent: Lungs, Thorax, or Respiration: structural or functional change in trachea or bronchi; Biochemical: Metabolism (Intermediary): effect on inflammation or mediation of inflammation
TDLo (Oral-Rat) 17,431 mg/kg/90 days-continuous: Behavioral :changes in motor activity (specific assay)
TDLo (Oral-Rat) 6846 mg/kg: female 1-22 day(s) after conception: Reproductive: Specific Developmental Abnormalities: homeostasis; Effects on Newborn: stillbirth, growth statistics (e.g.%, reduced weight gain)
TDLo (Oral-Chicken) 32,203 mg/kg/4 weeks-continuous: Endocrine: changes in growth hormone, evidence of thyroid hypofunction; Nutritional and Gross Metabolic: weight loss or decreased weight gain
TDLo (Oral-Chicken) 6468 mg/kg/1 week-intermittent: Endocrine: evidence of thyroid hypofunction; Blood: other changes; Nutritional and Gross Metabolic: weight loss or decreased weight gain
TDLo (Oral-Chicken) 12.93 gm/kg/2 weeks-intermittent: Endocrine: changes in growth hormone
TDLo (Oral-Chicken) 25.87 gm/kg/4 weeks-intermittent: Endocrine: other changes
TDLo (Oral-Mammal-Domestic) 3584 mg/kg/4 weeks-intermittent: Gastrointestinal: changes in structure or function of endocrine pancreas; Blood: changes in serum composition (e.g. TP, bilirubin, cholesterol); Nutritional and Gross Metabolic: changes in metals, not otherwise specified
TDLo (Intratracheal-Mammal-Species Unspecified) 250 mg/kg: Lungs, Thorax, or Respiration: structural or functional change in trachea or bronchi, other changes
DNA Adduct (Bacteria-*Escherichia coli*) 3000 ppm
Cytogenetic Analysis (Inhalation-Rat) 100 µg/m^3
Morphological Transformation (Hamster-Embryo) 1 mg/L

CARCINOGENIC INFORMATION: This product has not been tested for carcinogenic effect. Components, including the active ingredient are listed by agencies tracking the carcinogenic potential of chemical compounds, as follows:

MINERAL OIL: ACGIH TLV-A4 (Not Classifiable as a Human Carcinogen); IARC-3 (Unclassifiable as to Carcinogenicity in Humans)
ZINC OXIDE: EPA-II (Inadequate Information to Assess Carcinogenic Potential); EPA-D (Not Classifiable as to Human Carcinogenicity)

The remaining components of this product are not found on the following lists: U.S. EPA, U.S. NTP, U.S. OSHA, U.S. NIOSH, GERMAN MAK, IARC, or ACGIH and therefore are neither considered to be nor suspected to be cancer-causing agents by these agencies.

REPRODUCTIVE TOXICITY INFORMATION: This product has not been rated by the FDA for therapeutic risk in pregnancy.

Mutagenicity: It is not possible to conclude that Zinc Oxide is mutagenic, based on the limited information available. Mutagenic effects were observed in tests of blood cells of workers employed in zinc industries. However, because these workers are also exposed to compounds such as lead and cadmium, no specific conclusions can be drawn about Zinc Oxide. Urine from workers in the rubber industry, who were occupationally exposed to several chemicals including zinc oxide, did not cause gene mutations in bacteria. A positive result was obtained in a limited study using rats. An unconfirmed positive result was obtained in cultured mammalian cells and a negative result was obtained in bacteria.

Embryotoxicity/Teratogenicity: In general, zinc compounds are not known to cause developmental effects in the absence of maternal toxicity. There is no specific human information available for Zinc Oxide. In animal studies, harmful effects were observed in the offspring of rats in one study, which used high doses and did not evaluate maternal toxicity, and in another very limited study.

Reproductive Toxicity: There is no human information available for Zinc Oxide. The only animal study located for Zinc Oxide did not show reproductive effects. In general, zinc compounds are not known to cause reproductive toxicity.

ACGIH BIOLOGICAL EXPOSURE INDICES (BEIs): Currently, there are no ACGIH Biological Exposure Indices (BEIs) determined for components of this product.

12. ECOLOGICAL INFORMATION

ALL WORK PRACTICES MUST BE AIMED AT ELIMINATING ENVIRONMENTAL CONTAMINATION.

MOBILITY: This product has not been tested for soil absorption or mobility.

PERSISTENCE AND BIODEGRADABILITY: This product has not been tested for persistence or biodegradability.

BIOACCUMULATION: This product has not been tested for bioconcentration.

ECOTOXICITY: No specific information is currently available on the effect of this product on plants or animals in the environment. This product may be harmful to contaminated terrestrial and aquatic plant and animal life, especially in large quantities. The following data are available for the Zinc Oxide component.

ZINC OXIDE:
LD_{50} (*Lepomis macrochirus* Bluegill sunfish, weight 0.38 g) 96 hours = > 320 ppm

ZINC OXIDE (continued):
LD_{50} (*Oncorhynchus mykiss* Rainbow trout, weight 0.78 g) 96 hours = 1.1 ppm

RESULTS OF PBT AND vPvB ASSESSMENT: No Data Available. PBT and vPvB assessments are part of the chemical safety report required for some substances in European Union Regulation (EC) 1907/2006, Article 14.

ENVIRONMENTAL EXPOSURE CONTROLS: Controls should be engineered to prevent release to the environment, including procedures to prevent spills, atmospheric release and release to waterways.

OTHER ADVERSE EFFECTS: No component of this product is known to have ozone depletion potential.

13. DISPOSAL CONSIDERATIONS

DISPOSAL METHODS: It is the responsibility of the generator to determine at the time of disposal whether the product meets the criteria of a hazardous waste per regulations of the area in which the waste is generated and/or disposed of. Waste disposal must be in accordance with appropriate Federal, State, and local regulations. This product, if unaltered by use, may be disposed of by treatment at a permitted facility or as advised by your local hazardous waste regulatory authority. Shipment of wastes must be done with appropriately permitted and registered transporters.

DISPOSAL CONTAINERS: Waste materials must be placed in and shipped in appropriate 5-gallon or 55-gallon poly or metal waste pails or drums. Permeable cardboard containers are not appropriate and should not be used. Ensure that any required marking or labeling of the containers be done to all applicable regulations.

PRECAUTIONS TO BE FOLLOWED DURING WASTE HANDLING: Wear proper protective equipment when handling waste materials.

PREPARING WASTES FOR DISPOSAL: Waste disposal must be in accordance with appropriate U.S. Federal, State, and local regulations or with regulations of Canada. This product, if unaltered by handling, may be disposed of by treatment at a permitted facility or as advised by your local hazardous waste regulatory authority. All gowns, gloves, and disposable materials used in the preparation or handling of this product should be disposed of in accordance with established hazardous waste disposal procedures. Handle as if capable of transmitting infectious agents. Incineration is recommended. Reusable equipment should be cleaned with soap and water.

U.S. EPA WASTE NUMBER: Not applicable to wastes consisting only of this product.

EWC WASTE CODE: Wastes from Human or Animal Health Care or Related Research: 18 01 08: Medicines Other Than Those Mentioned in 18 01 07.

14. TRANSPORTATION INFORMATION

U.S. DEPARTMENT OF TRANSPORTATION SHIPPING REGULATIONS: This product is not classified as hazardous under regulations of U.S. DOT 49 CFR 172.101.

TRANSPORT CANADA TRANSPORTATION OF DANGEROUS GOODS REGULATIONS: This product is not classified as Dangerous Goods, per regulations of Transport Canada.

INTERNATIONAL AIR TRANSPORT ASSOCIATION (IATA): This product does not meet the criteria as Dangerous Goods, per rules of IATA.

INTERNATIONAL MARITIME ORGANIZATION (IMO) DESIGNATION: This product is NOT classified as Dangerous Goods by the International Maritime Organization.

EUROPEAN AGREEMENT CONCERNING THE INTERNATIONAL CARRIAGE OF DANGEROUS GOODS BY ROAD (ADR): This product does not meet the criteria as Dangerous Goods of the United Nations Economic Commission for Europe.

TRANSPORT IN BULK ACCORDING TO THE IBC CODE: Not applicable.

ENVIRONMENTAL HAZARDS: This product does not meet the criteria of environmentally hazardous according to the criteria of the UN Model Regulations (as reflected in the IMDG Code, ADR, RID, and ADN) and is not specifically listed in Annex III under MARPOL 73/78.

15. REGULATORY INFORMATION

UNITED STATES REGULATIONS:

U.S. SARA Reporting Requirements: The components of this product are not subject to the reporting requirements of Sections 302, 304, and 313 of Title III of the Superfund Amendments and Reauthorization Act.

U.S. SARA Threshold Planning Quantity (TPQ): There are no specific Threshold Planning Quantities for any component of this product. The default Federal SDS submission and inventory requirement filing threshold of 10,000 lb (4,540 kg) therefore applies, per 40 CFR 370.20.

U.S. CERCLA Reportable Quantities (RQ): Not applicable.

U.S. TSCA Inventory Status: This product is regulated by the Food and Drug Administration; it is not subject to requirements under TSCA.

California Safe Drinking Water and Toxic Enforcement Act (Proposition 65): No component is listed on the California Proposition 65 lists.

Other U.S. Federal Regulations: Regulations of the FDA under the Federal Food, Drug and Cosmetic Act are applicable when this material is used in pharmaceutical preparations. Under the Hazard Communication Standard (HCS), Section (b)(5)(ii) drugs are subject to labeling requirements by the FDA under the Federal Food, Drug and Cosmetic Act and are exempt from labeling provisions of the HCS; this section of the HCS exempts only labeling requirements and not requirements for a Safety Data Sheet for drugs.

CANADIAN REGULATIONS:

Canadian DSL/NDSL Inventory Status: This product regulated by the Therapeutic Products Programme (TPP) of Health Canada and so it is exempt from requirements of the DSL/NDSL Inventory.

Canadian Environmental Protection Act (CEPA) Priorities Substances Lists: No component is on the CEPA Priorities Substances List.

Other Canadian Regulations: Not applicable.

Canadian WHMIS Classification and Symbols: The WHMIS Requirements of the Hazardous Products Act does not apply in respect of the advertising, sale or importation of any cosmetic, device, drug or food within the meaning of the Food and Drugs Act.

15. REGULATORY INFORMATION (Continued)

EUROPEAN REGULATIONS:

Safety, Health, and Environmental Regulations/Legislation Specific for the Product: Formulated, finished medicinal products for human use are subject to Directive 2001/83/EC and subsequent amendments to the directive.

Chemical Safety Assessment: No Data Available. The chemical safety assessment is required for some substances according to European Union Regulation (EC) 1907/2006, Article 14.

16. OTHER INFORMATION

ANSI LABELING (Based on 129.1, Provided to Summarize Occupational Exposure Hazards): **CAUTION!** MAY BE HARMFUL IF ACCIDENTALLY INGESTED. MAY CAUSE SKIN IRRITATION. CONTAINS COMPOUND THAT IS ACUTELY AND CHRONICALLY TOXIC TO AQUATIC ORGANISMS. Do not taste or swallow. Avoid contact with skin or clothing. Avoid breathing mists or sprays. Keep container tightly closed. Use only with adequate ventilation. Wash thoroughly after handling. Wear gloves, goggles, and appropriate body protection during handling or administration. FIRST-AID: In case of contact, flush eyes with plenty of water. If inhaled, remove to fresh air. If not breathing, give artificial respiration. If breathing is difficult, give oxygen. If swallowed, call a physician immediately. Do NOT induce vomiting unless directed by a physician. Never give anything by mouth to an unconscious person. IN CASE OF FIRE: Use water fog, dry chemical, CO_2, or “alcohol” foam. IN CASE OF SPILL: Wipe up spilled product. Place residual in appropriate container and seal. Dispose of according to applicable regulations. Consult Safety Data Sheet for additional information.

GLOBAL HARMONIZATION AND EU CLP REGULATION (EC) 1272/2008 LABELING AND CLASSIFICATION: According to Article 1, item 5 (a) of CLP Regulation (EC) 1272/2008, medicinal products in the finished state for human use, as defined in 2001/83/EC, are excepted from classification and other criteria of 1272/2008.

67/548/EEC EU LABELING/CLASSIFICATION: According to Article 1 of European Union Council Directive 92/32/EEC, medical products in the finished state for human use (as defined by European Union Council Directives 67/548/EEC and 87/21/EEC) are not subject to the regulations and administrative provisions of European Union Council Directive 92/32/EEC.

CLASSIFICATION FOR COMPONENTS:

Full Text Global Harmonization AND EU CLP Regulation (EC) 1272/2008:

Zinc Oxide: This is a published and self-classification.
Classification: Acute Inhalation Toxicity Category 5, Aquatic Acute Toxicity Category 1, Aquatic Chronic Toxicity Category 1
Hazard Statements: H333: May be harmful if inhaled. H400: Very toxic to aquatic life. H410: Very toxic to aquatic life with long-lasting effects.

Mineral Oil: This is a self-classification.
Classification: Carcinogenic Category 2, Eye Irritation Category 2B
Hazard Statements: H351: Suspected of causing cancer. H320: Causes eye irritation.

White Petrolatum: The following is a Self-Classification.
Classification: Carcinogenic Category 1B
Hazard Statements: H350: May cause cancer.

All Other Components: No classification has been published or is applicable.

Full Text EU 67/548/EEC:

Zinc Oxide: This is a published and self-classification.
Classification: Dangerous for the Environment
Risk Phrases: R50/53: Very toxic to aquatic organisms, may cause long-term adverse effects in the aquatic environment.

Mineral Oil: This is a self-classification.
Classification: Carcinogenic Category 3
Risk Phrases: R40: Limited evidence of a carcinogenic effect.

White Petrolatum: The following is a Self-Classification.
Classification: Carcinogenic Category 2
Risk Phrases: R45: May cause cancer.

All Other Components: No classification has been published or is applicable.

This Safety Data Sheet is offered pursuant to OSHA’s Hazard Communication Standard, 29 CFR, 1910.1200. Other government regulations must be reviewed for applicability to this product. To the best of Fougera’s knowledge, the information contained herein is reliable and accurate as of this date; however, accuracy, suitability or completeness are not guaranteed and no warranties of any type, either express or implied, are provided. The information contained herein relates only to this specific product. If this product is combined with other materials, all component properties must be considered. Data may be changed from time to time. Be sure to consult the latest edition.

<u>REVISION DETAILS</u>: May 2015: Review and up-date SDS to comply with EU CLP and the Global Harmonization Standard.
<u>REFERENCES AND DATA SOURCES</u>: Contact the supplier for information.
<u>METHODS OF EVALUATING INFORMATION FOR THE PURPOSE OF CLASSIFICATION</u>: Bridging principles were used to classify this product.

PREPARED BY: CHEMICAL SAFETY ASSOCIATES, Inc. • PO Box 1961, Hilo, HI 96721 • 800/441-3365 • 808/969-4846
DATE OF PRINTING: June 27, 2015

DEFINITION OF TERMS

A large number of abbreviations and acronyms appear on a SDS. Some of these, which are commonly used, include the following:

CAS #: This is the Chemical Abstract Service Number that uniquely identifies each constituent.

EXPOSURE LIMITS IN AIR:

CEILING LEVEL: The concentration that shall not be exceeded during any part of the working exposure.

DFG MAK Germ Cell Mutagen Categories: 1: Germ cell mutagens that have been shown to increase the mutant frequency in the progeny of exposed humans. **2:** Germ cell mutagens that have been shown to increase the mutant frequency in the progeny of exposed mammals.

EXPOSURE LIMITS IN AIR (continued):

DFG MAK Germ Cell Mutagen Categories (continued): 3A: Substances that have been shown to induce genetic damage in germ cells of human of animals, or which produce mutagenic effects in somatic cells of mammals *in vivo* and have been shown to reach the germ cells in an active form. **3B:** Substances that are suspected of being germ cell mutagens because of their genotoxic effects in mammalian somatic cell *in vivo*; in exceptional cases, substances for which there are no *in vivo* data, but that are clearly mutagenic in vitro and structurally related to known in vivo mutagens.

DEFINITION OF TERMS (Continued)

EXPOSURE LIMITS IN AIR (continued):

DFG MAK Germ Cell Mutagen Categories (continued): 4: Not applicable (Category 4 carcinogenic substances are those with non-genotoxic mechanisms of action. By definition, germ cell mutagens are genotoxic. Therefore, a Category 4 for germ cell mutagens cannot apply. At some time in the future, it is conceivable that a Category 4 could be established for genotoxic substances with primary targets other than DNA [e.g. purely aneugenic substances] if research results make this seem sensible.) **5:** Germ cell mutagens, the potency of which is considered to be so low that, provided the MAK value is observed, their contribution to genetic risk for humans is expected not to be significant.

DFG MAK Pregnancy Risk Group Classification: Group A: A risk of damage to the developing embryo or fetus has been unequivocally demonstrated. Exposure of pregnant women can lead to damage of the developing organism, even when MAK and BAT (Biological Tolerance Value for Working Materials) values are observed. **Group B:** Currently available information indicates a risk of damage to the developing embryo or fetus must be considered to be probable. Damage to the developing organism cannot be excluded when pregnant women are exposed, even when MAK and BAT values are observed. **Group C:** There is no reason to fear a risk of damage to the developing embryo or fetus when MAK and BAT values are observed. **Group D:** Classification in one of the groups A–C is not yet possible because, although the data available may indicate a trend, they are not sufficient for final evaluation.

IDLH: Immediately Dangerous to Life and Health. This level represents a concentration from which one can escape within 30-minutes without suffering escape-preventing or permanent injury.

LOQ: Limit of Quantitation.

MAK: Federal Republic of Germany Maximum Concentration Values in the workplace.

NE: Not Established. When no exposure guidelines are established, an entry of NE is made for reference.

NIC: Notice of Intended Change.

NIOSH CEILING: The exposure that shall not be exceeded during any part of the workday. If instantaneous monitoring is not feasible, the ceiling shall be assumed as a 15-minute TWA exposure (unless otherwise specified) that shall not be exceeded at any time during a workday.

NIOSH RELs: NIOSH's Recommended Exposure Limits.

PEL: OSHA's Permissible Exposure Limits. This exposure value means exactly the same as a TLV, except that it is enforceable by OSHA. The OSHA Permissible Exposure Limits are based in the 1989 PELs and the June, 1993 Air Contaminants Rule (Federal Register: 58: 35338-35351 and 58: 40191). Both the current PELs and the vacated PELs are indicated. The phrase, "Vacated 1989 PEL" is placed next to the PEL that was vacated by Court Order.

SKIN: Used when a there is a danger of cutaneous absorption.

STEL: Short Term Exposure Limit, usually a 15-minute time-weighted average (TWA) exposure that should not be exceeded at any time during a workday, even if the 8-hr TWA is within the TLV-TWA, PEL-TWA or REL-TWA.

TLV: Threshold Limit Value. An airborne concentration of a substance that represents conditions under which it is generally believed that nearly all workers may be repeatedly exposed without adverse effect. The duration must be considered, including the 8-hour.

TWA: Time Weighted Average exposure concentration for a conventional 8-hr (TLV, PEL) or up to a 10-hr (REL) workday and a 40-hr workweek.

WEEL: Workplace Environmental Exposure Limits from the AIHA.

HAZARDOUS MATERIALS IDENTIFICATION SYSTEM HAZARD RATINGS:

This rating system was developed by the National Paint and Coating Association and has been adopted by industry to identify the degree of chemical hazards.

<u>HEALTH HAZARD</u>: **0** <u>Minimal Hazard</u>: No significant health risk, irritation of skin or eyes not anticipated. *Skin Irritation*: Essentially non-irritating. Mechanical irritation may occur. PII or Draize = 0. *Eye Irritation*: Essentially non-irritating, minimal effects clearing in < 24 hours. Mechanical irritation may occur. Draize = 0. *Oral Toxicity LD_{50} Rat*: > 5000 mg/kg. *Dermal Toxicity LD_{50} Rat or Rabbit*: > 2000 mg/kg. *Inhalation Toxicity 4-hrs LC_{50} Rat*: > 20 mg/L. **1:** <u>Slight Hazard</u>: Minor reversible injury may occur; may irritate the stomach if swallowed; may defat the skin and exacerbate existing dermatitis. *Skin Irritation*: Slightly or mildly irritating. PII or Draize > 0 < 5. *Eye Irritation*: Slightly to mildly irritating, but reversible within 7 days. Draize > 0 ≤ 25. *Oral Toxicity LD_{50} Rat*: > 500–5000 mg/kg. *Dermal Toxicity LD_{50} Rat or Rabbit*: > 1000–2000 mg/kg. *Inhalation Toxicity LC_{50} 4-hrs Rat*: > 2–20 mg/L. **2** *Moderate Hazard*: Temporary or transitory injury may occur; prolonged exposure may affect the CNS. *Skin Irritation*: Moderately irritating; primary irritant; sensitizer. PII or Draize ≥ 5, with no destruction of dermal tissue. *Eye Irritation*: Moderately to severely irritating; reversible corneal opacity; corneal involvement or irritation clearing in 8–21 days. Draize = 26–100, with reversible effects. *Oral Toxicity LD_{50} Rat*: > 50–500 mg/kg. *Dermal Toxicity LD_{50} Rat or Rabbit*: > 200–1000 mg/kg. *Inhalation Toxicity LC_{50} 4-hrs Rat*: > 0.5–2 mg/L. **3** <u>Serious Hazard</u>: Major injury likely unless prompt action is taken and medical treatment is given; high level of toxicity; corrosive. *Skin Irritation*: Severely irritating and/or corrosive; may cause destruction of dermal tissue, skin burns, and dermal necrosis. PII or Draize > 5–8, with destruction of tissue. *Eye Irritation*: Corrosive, irreversible destruction of ocular tissue; corneal involvement or irritation persisting for more than 21 days. Draize > 80 with effects irreversible in 21 days. *Oral Toxicity LD_{50} Rat*: > 1–50 mg/kg. *Dermal Toxicity LD_{50} Rat or Rabbit*: > 20–200 mg/kg. *Inhalation Toxicity LC_{50} 4-hrs Rat*: > 0.05–0.5 mg/L. **4** <u>Severe Hazard</u>: Life-threatening; major or permanent damage may result from single or repeated exposure; extremely toxic; irreversible injury may result from brief contact. *Skin Irritation*: Not appropriate. Do not rate as a 4, based on skin irritation alone. *Eye Irritation*: Not appropriate. Do not rate as a 4, based on eye irritation alone. *Oral Toxicity LD_{50} Rat*: ≤ 1 mg/kg. *Dermal Toxicity LD_{50} Rat or Rabbit*: ≤ 20 mg/kg. *Inhalation Toxicity LC_{50} 4-hrs Rat*: ≤ 0.05 mg/L.

<u>FLAMMABILITY HAZARD</u>: **0** <u>Minimal Hazard</u>: Materials that will not burn in air when exposure to a temperature of 815.5°C (1500°F) for a period of 5 minutes. **1** <u>Slight Hazard</u>: Materials that must be pre-heated before ignition can occur. Material requires considerable pre-heating, under all ambient temperature conditions before ignition and combustion can occur. This usually includes the following: Materials that will burn in air when exposed to a temperature of 815.5°C (1500°F) for a period of 5 minutes or less; Liquids, solids and semisolids having a flash point at or above 93.3°C (200°F) (i.e. OSHA Class IIIB); and Most ordinary combustible materials (e.g. wood, paper, etc.). **2** <u>Moderate Hazard</u>: Materials that must be moderately heated or exposed to relatively high ambient temperatures before ignition can occur. Materials in this degree would not, under normal conditions, form hazardous atmospheres in air, but under high ambient temperatures or moderate heating may release vapor in sufficient quantities to produce hazardous atmospheres with air.

HAZARDOUS MATERIALS IDENTIFICATION SYSTEM HAZARD RATINGS (continued):

<u>FLAMMABILITY HAZARD (continued)</u>: **2 (continued):** This usually includes the following: Liquids having a flash-point at or above 37.8°C (100°F); Solid materials in the form of course dusts that may burn rapidly but that generally do not form explosive atmospheres; Solid materials in a fibrous or shredded form that may burn rapidly and create flash fire hazards (e.g. cotton, sisal, hemp); and Solids and semisolids (e.g. viscous and slow flowing as asphalt) that readily give off flammable vapors. **3** <u>Serious Hazard</u>: Liquids and solids that can be ignited under almost all ambient temperature conditions. Materials in this degree produce hazardous atmospheres with air under almost all ambient temperatures, or, unaffected by ambient temperature, are readily ignited under almost all conditions. This usually includes the following: Liquids having a flash point below 22.8°C (73°F) and having a boiling point at or above 38°C (100°F) and those liquids having a flash point at or above 22.8°C (73°F) and below 37.8°C (100°F) (i.e. OSHA Class IB and IC); Materials that on account of their physical form or environmental conditions can form explosive mixtures with air and are readily dispersed in air (e.g., dusts of combustible solids, mists or droplets of flammable liquids); and Materials that burn extremely rapidly, usually by reason of self-contained oxygen (e.g. dry nitrocellulose and many organic peroxides). **4** <u>Severe Hazard</u>: Materials that will rapidly or completely vaporize at atmospheric pressure and normal ambient temperature or that are readily dispersed in air, and that will burn readily. This usually includes the following: Flammable gases; Flammable cryogenic materials; Any liquid or gaseous material that is liquid while under pressure and has a flash point below 22.8°C (73°F) and a boiling point below 37.8°C (100°F) (i.e. OSHA Class IA); and Materials that ignite spontaneously when exposed to air at a temperature of 54.4°C (130°F) or below (pyrophoric).

<u>PHYSICAL HAZARD</u>: **0** *Water Reactivity*: Materials that do not react with water. *Organic Peroxides*: Materials that are normally stable, even under fire conditions and will not react with water. *Explosives*: Substances that are Non-Explosive. *Compressed Gases*: No Rating. *Pyrophorics*: No Rating. *Oxidizers*: No 0 rating. *Unstable Reactives*: Substances that will not polymerize, decompose, condense, or self-react.) **1** *Water Reactivity*: Materials that change or decompose upon exposure to moisture. *Organic Peroxides*: Materials that are normally stable, but can become unstable at high temperatures and pressures. These materials may react with water, but will not release energy violently. *Explosives*: Division 1.5 & 1.6 explosives. Substances that are very insensitive explosives or that do not have a mass explosion hazard. *Compressed Gases*: Pressure below OSHA definition. *Pyrophorics*: No Rating. *Oxidizers*: Packaging Group III oxidizers; Solids: any material that in either concentration tested, exhibits a mean burning time less than or equal to the mean burning time of a 3:7 potassium bromate/cellulose mixture and the criteria for Packing Group I and II are not met. Liquids: any material that exhibits a mean pressure rise time less than or equal to the pressure rise time of a 1:1 nitric acid (65%)/cellulose mixture and the criteria for Packing Group I and II are not met. *Unstable Reactives*: Substances that may decompose condense, or self-react, but only under conditions of high temperature and/or pressure and have little or no potential to cause significant heat generation or explosion hazard. Substances that readily undergo hazardous polymerization in the absence of inhibitors. Substances that readily undergo hazardous polymerization in the absence of inhibitors. **2** *Water Reactivity*: Materials that may react violently with water. *Organic Peroxides*: Materials that, in themselves, are normally unstable and will readily undergo violent chemical change, but will not detonate. These materials may also react violently with water. *Explosives*: Division 1.4 explosives. Explosive substances where the explosive effects are largely confined to the package and no projection of fragments of appreciable size or range are expected. An external fire must not cause virtually instantaneous explosion of almost the entire contents of the package. *Compressed Gases*: Pressurized and meet OSHA definition but < 514.7 psi absolute at 21.1°C (70°F) [500 psig]. *Pyrophorics*: No Rating. *Oxidizers*: Packing Group II oxidizers. Solids: any material that, either in concentration tested, exhibits a mean burning time of less than or equal to the mean burning time of a 2:3 potassium bromate/cellulose mixture and the criteria for Packing Group I are not met. Liquids: any material that exhibits a mean pressure rise time less than or equal to the pressure rise of a 1:1 aqueous sodium chlorate solution (40%)/cellulose mixture and the criteria for Packing Group I are not met. *Reactives*: Substances that may polymerize, decompose, condense, or self-react at ambient temperature and/or pressure, but have a low potential (or low risk) for significant heat generation or explosion. Substances that readily form peroxides upon exposure to air or oxygen at room temperature. **3** *Water Reactivity*: Materials that may form explosive reactions with water. *Organic Peroxides*: Materials that are capable of detonation or explosive reaction, but require a strong initiating source or must be heated under confinement before initiation; or materials that react explosively with water. *Explosives*: Division 1.3 explosives. Explosive substances that have a fire hazard and either a minor blast hazard or a minor projection hazard or both, but do not have a mass explosion hazard. *Compressed Gases*: Pressure ≥ 514.7 psi absolute at 21.1°C (70°F) [500 psig]. *Pyrophorics*: No Rating. *Oxidizers*: Packing Group I oxidizers. Solids: any material that, in either concentration tested, exhibits a mean burning time less than the mean burning time of a 3:2 potassium bromate/cellulose mixture. Liquids: any material that spontaneously ignites when mixed with cellulose in a 1:1 ratio, or which exhibits a mean pressure rise time less than the pressure rise time of a 1:1 perchloric acid (50%)/cellulose mixture. *Unstable Reactives*: Substances that may polymerize, decompose, condense, or self-react at ambient temperature and/or pressure and have a moderate potential (or moderate risk) to cause significant heat generation or explosion. **4** *Water Reactivity*: Materials that react explosively with water without requiring heat or confinement. *Organic Peroxides*: Materials that are readily capable of detonation or explosive decomposition at normal temperature and pressures. *Explosives*: Division 1.1 & 1.2 explosives. Explosive substances that have a mass explosion hazard or have a projection hazard. A mass explosion is one that affects almost the entire load instantaneously. *Compressed Gases*: No Rating. *Pyrophorics*: Add to the definition of Flammability 4. *Oxidizers*: No 4 rating. *Unstable Reactives*: Substances that may polymerize, decompose, condense, or self-react at ambient temperature and/or pressure and have a high potential (or high risk) to cause significant heat generation or explosion.

NATIONAL FIRE PROTECTION ASSOCIATION HAZARD RATINGS:

<u>HEALTH HAZARD</u>: **0** Materials that, under emergency conditions, would offer no hazard beyond that of ordinary combustible materials. Gases and vapors with an LC_{50} for acute inhalation toxicity greater than 10,000 ppm. Dusts and mists with an LC_{50} for acute inhalation toxicity greater than 200 mg/L. Materials with an LD_{50} for acute dermal toxicity greater than 2000 mg/kg. Materials with an LD_{50} for acute oral toxicity greater than 2000 mg/kg. Materials essentially non-irritating to the respiratory tract, eyes, and skin.

DEFINITION OF TERMS (Continued)

NATIONAL FIRE PROTECTION ASSOCIATION HAZARD RATINGS (continued):

HEALTH HAZARD (continued): **1** Materials that, under emergency conditions, can cause significant irritation. Gases and vapors with an LC_{50} for acute inhalation toxicity greater than 5,000 ppm but less than or equal to 10,000 ppm. Dusts and mists with an LC_{50} for acute inhalation toxicity greater than 10 mg/L but less than or equal to 200 mg/L. Materials with an LD_{50} for acute dermal toxicity greater than 1000 mg/kg but less than or equal to 2000 mg/kg. Materials that slightly to moderately irritate the respiratory tract, eyes and skin. Materials with an LD_{50} for acute oral toxicity greater than 500 mg/kg but less than or equal to 2000 mg/kg. **2** Materials that, under emergency conditions, can cause temporary incapacitation or residual injury. Gases with an LC_{50} for acute inhalation toxicity greater than 3,000 ppm but less than or equal to 5,000 ppm. Any liquid whose saturated vapor concentration at 20°C (68°F) is equal to or greater than one-fifth its LC_{50} for acute inhalation toxicity, if its LC_{50} is less than or equal to 5000 ppm and that does not meet the criteria for either degree of hazard 3 or degree of hazard 4. Dusts and mists with an LC_{50} for acute inhalation toxicity greater than 2 mg/L but less than or equal to 10 mg/L. Materials with an LD_{50} for acute dermal toxicity greater than 200 mg/kg but less than or equal to 1000 mg/kg. Compressed liquefied gases with boiling points between -30°C (-22°F) and -55°C (-66.5°F) that cause severe tissue damage, depending on duration of exposure. Materials that are respiratory irritants. Materials that cause severe, but reversible irritation to the eyes or are lachrymators. Materials that are primary skin irritants or sensitizers. Materials whose LD_{50} for acute oral toxicity is greater than 50 mg/kg but less than or equal to 500 mg/kg. **3** Materials that, under emergency conditions, can cause serious or permanent injury. Gases with an LC_{50} for acute inhalation toxicity greater than 1,000 ppm but less than or equal to 3,000 ppm. Any liquid whose saturated vapor concentration at 20°C (68°F) is equal to or greater its LC_{50} for acute inhalation toxicity, if its LC_{50} is less than or equal to 3000 ppm and that does not meet the criteria for degree of hazard 4. Dusts and mists with an LC_{50} for acute inhalation toxicity greater than 0.5 mg/L but less than or equal to 2 mg/L. Materials with an LD_{50} for acute dermal toxicity greater than 40 mg/kg but less than or equal to 200 mg/kg. Materials that are corrosive to the respiratory tract. Materials that are corrosive to the eyes or cause irreversible corneal opacity. Materials corrosive to the skin. Cryogenic gases that cause frostbite and irreversible tissue damage. Compressed liquefied gases with boiling points below -55°C (-66.5°F) that cause frostbite and irreversible tissue damage. Materials with an LD_{50} for acute oral toxicity greater than 5 mg/kg but less than or equal to 50 mg/kg. **4** Materials that, under emergency conditions, can be lethal. Gases with an LC_{50} for acute inhalation toxicity less than or equal to 1,000 ppm. Any liquid whose saturated vapor concentration at 20°C (68°F) is equal to or greater than ten times its LC_{50} for acute inhalation toxicity, if its LC_{50} is less than or equal to 1000 ppm. Dusts and mists whose LC_{50} for acute inhalation toxicity is less than or equal to 0.5 mg/L. Materials whose LD_{50} for acute dermal toxicity is less than or equal to 40 mg/kg. Materials whose LD_{50} for acute oral toxicity is less than or equal to 5 mg/kg.

FLAMMABILITY HAZARD: **0** Materials that will not burn under typical fire conditions, including intrinsically noncombustible materials such as concrete, stone, and sand. Materials that will not burn in air when exposed to a temperature of 816°C (1500°F) for a period of 5 minutes in according with Annex D of NFPA 704. **1** Materials that must be preheated before ignition can occur. Materials in this degree require considerable preheating, under all ambient temperature conditions, before ignition and combustion can occur: Materials that will burn in air when exposed to a temperature of 816°C (1500°F) for a period of 5 minutes in according with Annex D of NFPA 704. Liquids, solids, and semisolids having a flash point at or above 93.4°C (200°F) (i.e. Class IIIB liquids). Liquids with a flash point greater than 35°C (95°F) that do not sustain combustion when tested using the *Method of Testing for Sustained Combustibility*, per 49 CFR 173, Appendix H or the UN *Recommendations on the Transport of Dangerous Goods, Model Regulations* (current edition) and the related *Manual of Tests and Criteria* (current edition). Liquids with a flash point greater than 35°C (95°F) in a water-miscible solution or dispersion with a water non-combustible liquid/solid content of more than 85% by weight. Liquids that have no fire point when tested by ASTM D 92, *Standard Test Method for Flash and Fire Points by Cleveland Open Cup*, up to the boiling point of the liquid or up to a temperature at which the sample being tested shows an obvious physical change. Combustible pellets with a representative diameter of greater than 2 mm (10 mesh). Most ordinary combustible materials. Solids containing greater than 0.5% by weight of a flammable or combustible solvent are rated by the closed cup flash point of the solvent. **2** Materials that must be moderately heated or exposed to relatively high ambient temperatures before ignition can occur. Materials in this degree would not under normal conditions form hazardous atmospheres with air, but under high ambient temperatures or under moderate heating could release vapor in sufficient quantities to produce hazardous atmospheres with air. Liquids having a flash point at or above 37.8°C (100°F) and below 93.4°C (200°F) (i.e. Class II and Class IIIA liquids.) Solid materials in the form of powders or coarse dusts of representative diameter between 420 microns (40 mesh) and 2 mm (10 mesh) that burn rapidly but that generally do not form explosive mixtures with air. Solid materials in fibrous or shredded form that burn rapidly and create flash fire hazards, such as cotton, sisal, and hemp. Solids and semisolids that readily give off flammable vapors. Solids containing greater than 0.5% by weight of a flammable or combustible solvent are rated by the closed cup flash point of the solvent. **3** Liquids and solids that can be ignited under almost all ambient temperature conditions. Materials in this degree produce hazardous atmospheres with air under almost all ambient temperatures or, though unaffected by ambient temperatures, are readily ignited under almost all conditions. Liquids having a flash point below 22.8°C (73°F) and having a boiling point at or above 37.8°C (100°F) and those liquids having a flash point at or above 22.8°C (73°F) and below 37.8°C (100°F) (i.e. Class IB and IC liquids). Materials that on account of their physical form or environmental conditions can form explosive mixtures with air and are readily dispersed in air. Flammable or combustible dusts with representative diameter less than 420 microns (40 mesh). Materials that burn with extreme rapidity, usually by reason of self-contained oxygen (e.g. dry nitrocellulose and many organic peroxides). Solids containing greater than 0.5% by weight of a flammable or combustible solvent are rated by the closed cup flash point of the solvent.

NATIONAL FIRE PROTECTION ASSOCIATION HAZARD RATINGS (continued):

FLAMMABILITY HAZARD (continued): **4** Materials that will rapidly or completely vaporize at atmospheric pressure and normal ambient temperature or that are readily dispersed in air and will burn readily. Flammable gases. Flammable cryogenic materials. Any liquid or gaseous materials that is liquid while under pressure and has a flash point below 22.8°C (73°F) and a boiling point below 37.8°C (100°F) (i.e. Class IA liquids). Materials that ignite when exposed to air, Solids containing greater than 0.5% by weight of a flammable or combustible solvent are rated by the closed cup flash point of the solvent.

INSTABILITY HAZARD: **0** Materials that in themselves are normally stable, even under fire conditions. Materials that have an instantaneous power density (product of heat of reaction and reaction rate) at 250°C (482°F) below 0.01 W/mL. Materials that do not exhibit an exotherm at temperatures less than or equal to 500°C (932°F) when tested by differential scanning calorimetry. **1** Materials that in themselves are normally stable, but that can become unstable at elevated temperatures and pressures. Materials that have an instantaneous power density (product of heat of reaction and reaction rate) at 250°C (482°F) at or above 0.01 W/mL and below 10 W/mL. **2** Materials that readily undergo violent chemical change at elevated temperatures and pressures. Materials that have an instantaneous power density (product of heat of reaction and reaction rate) at 250°C (482°F) at or above 10 W/mL and below 100W/mL. **3** Materials that in themselves are capable of detonation or explosive decomposition or explosive reaction, but that require a strong initiating source or that must be heated under confinement before initiation. Materials that have an estimated instantaneous power density (product of heat of reaction and reaction rate) at 250°C (482°F) at or above 100 W/mL and below 1000 W/mL. Materials that are sensitive to thermal or mechanical shock at elevated temperatures and pressures. **4** Materials that in themselves are readily capable of detonation or explosive decomposition or explosive reaction at normal temperatures and pressures. Materials that are sensitive to localized thermal or mechanical shock at normal temperatures and pressures. Materials that have an estimated instantaneous power density (product of heat of reaction and reaction rate) at 250°C (482°F) of 1000 W/mL or greater.

FLAMMABILITY LIMITS IN AIR:

Much of the information related to fire and explosion is derived from the National Fire Protection Association (**NFPA**). Flash Point: Minimum temperature at which a liquid gives off sufficient vapor to form an ignitable mixture with air near the surface of the liquid or within the test vessel used. Autoignition Temperature: Minimum temperature of a solid, liquid, or gas required to initiate or cause self-sustained combustion in air with no other source of ignition. LEL: Lowest concentration of a flammable vapor or gas/air mixture that will ignite and burn with a flame. UEL: Highest concentration of a flammable vapor or gas/air mixture that will ignite and burn with a flame.

TOXICOLOGICAL INFORMATION:

Human and Animal Toxicology: Possible health hazards as derived from human data, animal studies, or from the results of studies with similar compounds are presented. LD_{50}: Lethal Dose (solids & liquids) that kills 50% of the exposed animals. LC_{50}: Lethal Concentration (gases) that kills 50% of the exposed animals. ppm: Concentration expressed in parts of material per million parts of air or water. mg/m^3: Concentration expressed in weight of substance per volume of air. mg/kg: Quantity of material, by weight, administered to a test subject, based on their body weight in kg. TDLo: Lowest dose to cause a symptom. TCLo: Lowest concentration to cause a symptom. TDo, LDLo, and LDo, or TC, TCo, LCLo, and LCo: Lowest dose (or concentration) to cause lethal or toxic effects. **Cancer Information:** IARC: International Agency for Research on Cancer. NTP: National Toxicology Program. RTECS: Registry of Toxic Effects of Chemical Substances. IARC and NTP rate chemicals on a scale of decreasing potential to cause human cancer with rankings from 1 to 4. Subrankings (2A, 2B, etc.) are also used. **Other Information:** BEI: ACGIH Biological Exposure Indices, represent the levels of determinants which are most likely to be observed in specimens collected from a healthy worker who has been exposed to chemicals to the same extent as a worker with inhalation exposure to the TLV.

REPRODUCTIVE TOXICITY INFORMATION:

A mutagen is a chemical that causes permanent changes to genetic material (DNA) such that the changes will propagate through generation lines. An embryo toxin is a chemical that causes damage to a developing embryo (i.e. within the first eight weeks of pregnancy in humans), but the damage does not propagate across generational lines. A teratogen is a chemical that causes damage to a developing fetus, but the damage does not propagate across generational lines. A reproductive toxin is any substance that interferes in any way with the reproductive process.

ECOLOGICAL INFORMATION:

EC: Effect concentration in water. BCF: Bioconcentration Factor, which is used to determine if a substance will concentrate in life forms that consume contaminated plant or animal matter. TLm: Median threshold limit. $\log K_{OW}$ or $\log K_{OC}$: Coefficient of Oil/Water Distribution is used to assess a substance's behavior in the environment.

REGULATORY INFORMATION:

U.S. and CANADA:

This section explains the impact of various laws and regulations on the material. EPA: U.S. Environmental Protection Agency. ACGIH: American Conference of Governmental Industrial Hygienists, a professional association that establishes exposure limits. OSHA: U.S. Occupational Safety and Health Administration. NIOSH: National Institute of Occupational Safety and Health, which is the research arm of OSHA. WHMIS: Canadian Workplace Hazardous Materials Information System. DOT: U.S. Department of Transportation. TC: Transport Canada. SARA: Superfund Amendments and Reauthorization Act. DSL/NDSL: Canadian Domestic/Non-Domestic Substances List. TSCA: U.S. Toxic Substance Control Act. CERCLA: Comprehensive Environmental Response, Compensation, and Liability Act. Marine Pollutant status according to the DOT; CERCLA or Superfund; and various state regulations. This section also includes information on the precautionary warnings that appear on the material's package label.
