# Supplementary material for: Topical wound-care products and their effects on healing, inflammatory biomarkers, and growth in piglets undergoing castration
Source: Porcine Health Manag. 2026 Apr 21;12:23. doi: 10.1186/s40813-026-00492-7 (PMC13097753; doi:10.1186/s40813-026-00492-7)
Supplement: Supplementary file 3 — Supplementary Material 3 [file 40813_2026_492_MOESM3_ESM.pdf]

OINKMENT- ethyl alcohol, sodium propionate liquid
Animal Science Products, Inc.

Disclaimer: This drug has not been found by FDA to be safe and effective, and this labeling has not been approved by FDA. For further information about unapproved drugs, click here.

Oinkment

Active Ingredient

Ethyl Alcohol......................................72.6%

Sodium Propionate..............................0.5%

Purpose

QUICK-DRYING ANTIMICROBIAL FLEXIBLE FILM BANDAGE

FOR VETERINARY USE ONLY

Uses

For use as a topical microbiocidal barrier on the skin of swine to shield from abrasion and guard against contamination that may cause infection

Warnings

- **For external use only.**
- Store at room temperature.
- Not for human use.
- Keep out of reach of children.

Directions

Shake well before use. For best results, clean and dry the wound and surrounding skin for good adhesion. Apply Oinkment with spray tip 6 inches from wound, spraying to completely cover the wound and overlap onto surrounding healthy skin.

Reapply every other day or as required to keep the wound sealed until satisfactory.

ADVANTAGES

• Easy to spray, dries fast to form a flexible film bandage for abrasions, cuts and scrapes
• Covers and seals wound to maintain clean, moist healing environment
• Antibacterial and antifungal activity

• Deters tail biting
• Soothing analgesic action
• Highly visible
• Safe, non-toxic, 100% GRAS (21 CFR) food approved components
• No preslaughter withdrawal required
• Removal and cleanup with water

Keep Out Of reach of Children

Inactive Ingredient

Anhydrous citric acid, Water, FD&C yellow no. 5, FD&C blue no. 1, Methylcellulose

Product label

Bottle label

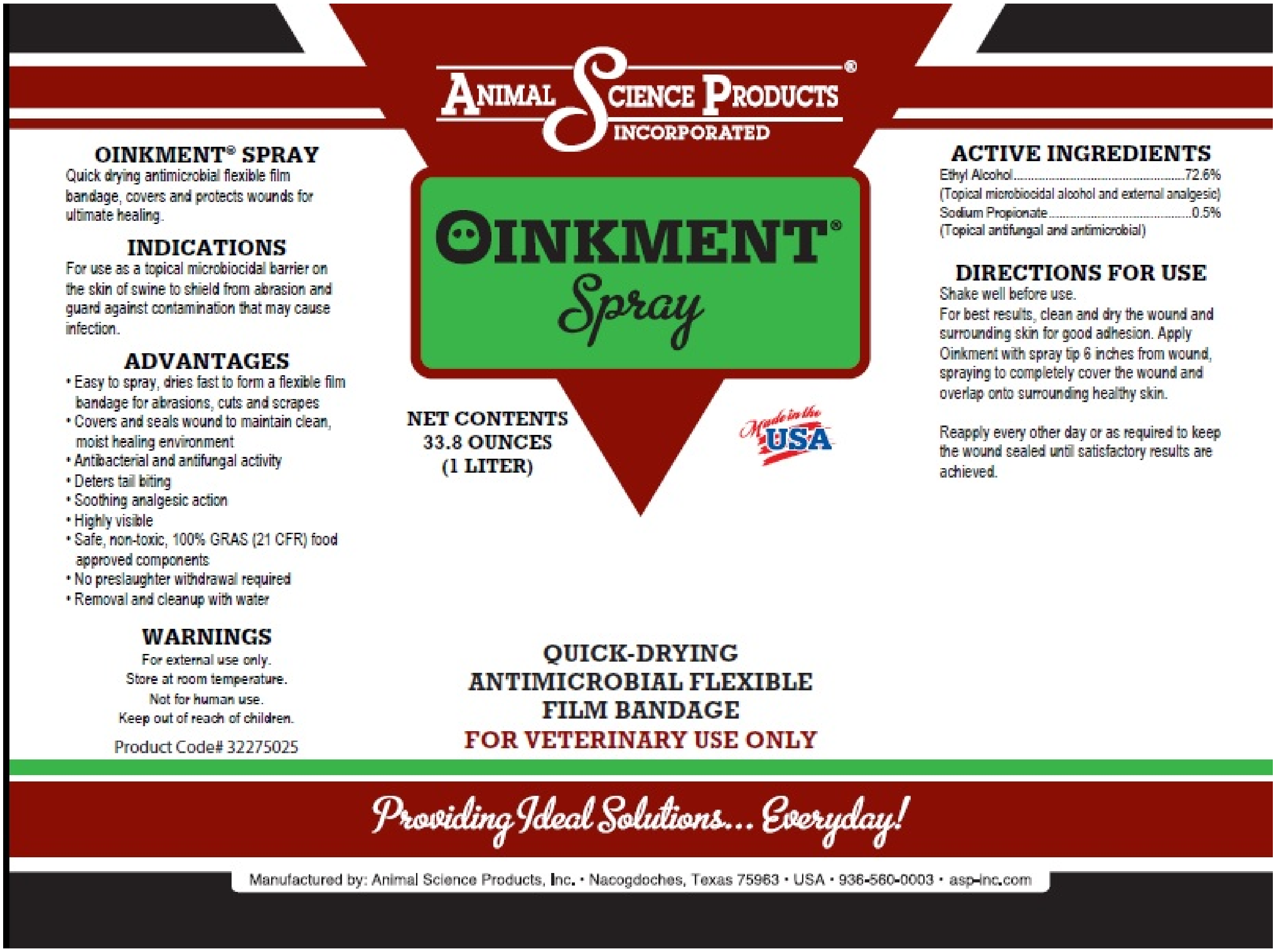

Product label

Box label

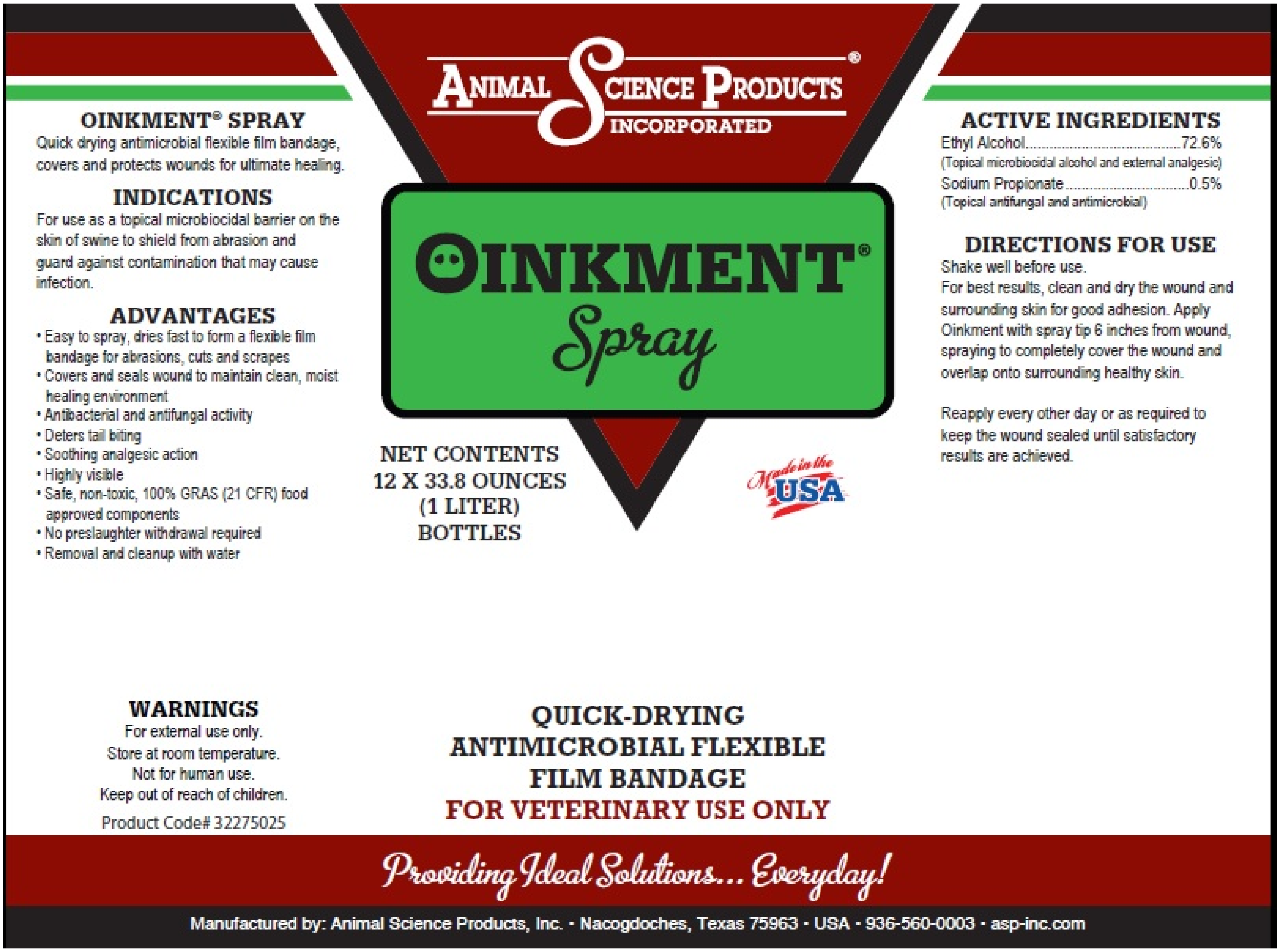

OINKMENT

ethyl alcohol, sodium propionate liquid

Product Information

**Product Type**	OTC ANIMAL DRUG	**Item Code (Source)**	NDC:57932-005
**Route of Administration**	TOPICAL		

Active Ingredient/Active Moiety

Ingredient Name	Basis of Strength	Strength
**ALCOHOL** (UNII: 3K9958V90M) (ALCOHOL - UNII:3K9958V90M)	ALCOHOL	7.2 mg in 1 mL
**SODIUM PROPIONATE** (UNII: DK6Y9P42IN) (PROPIONIC ACID - UNII:JHU490RVYR)	SODIUM PROPIONATE	0.5 mg in 1 mL

Inactive Ingredients

Ingredient Name	Strength
**WATER** (UNII: 059QF0KO0R)	

**ANHYDROUS CITRIC ACID** (UNII: XF417D3PSL)	
**METHYLCELLULOSE, UNSPECIFIED** (UNII: Z944H5SN0H)	
**FD&C YELLOW NO. 5** (UNII: I753WB2F1M)	
**FD&C BLUE NO. 1** (UNII: H3R47K3TBD)	

Packaging

#	Item Code	Package Description	Marketing Start Date	Marketing End Date
**1**	NDC:57932-005-01	12 in 1 BOX		
**1**		1000 mL in 1 BOTTLE		

Marketing Information

Marketing Category	Application Number or Monograph Citation	Marketing Start Date	Marketing End Date
unapproved drug other		05/14/2020	

Labeler - Animal Science Products, Inc. (151824091)

Establishment

Name	Address	ID/FEI	Business Operations
Animal Science Products Inc.		151824091	manufacture

Establishment

Name	Address	ID/FEI	Business Operations
Delta Synthetic Co., Ltd.		656128618	api manufacture

Revised: 10/2025 Animal Science Products, Inc.

SECTION 1: Identification

1.1 Identification

Product Form: Mixture
Product Name: Oinkment®

COMPANY: Animal Science Products, Inc.
3418 Rayburn Drive
Nacogdoches, TX 75961
Phone: 936-560-0003

1.2 Recommended Use

Use of the Mixture: Antimicrobial flexible film bandage

1.3 Emergency Contact

Infotrac
Domestic contact: 800-535-5053
International contact: +1-352-323-3500

SECTION 2: Hazard(s) Identification

2.1 Classification of the substance or mixture

GHS-US Classification

P210 Keep away from heat/sparks/open flames/hot surfaces – No smoking
P233 Keep container tightly closed
P240 Ground/bond container and receiving equipment
P241 Use explosion-proof electrical/ventilating/light/…/equipment
P242 Use only non-sparking tools
P243 Take precautionary measures against static discharge
P264 Wash exposed skin thoroughly after handling (refer to section 7.1 – Precaution for Safe Handling)
P280 Wear protective gloves/protective clothing/eye protection/face protection
P303+P361+P353 IF ON SKIN (or hair): Remove/Take off immediately all contaminated clothing. Rinse skin with water/shower
P305+P351+P338 IF IN EYES: Rinse continuously with water for several minutes. Remove contact lenses if present and easy to do – continue rinsing
P337+P313 If eye irritation persists, get medical advice/attention
P370+P378 In case of fire: Use dry chemical, water spray, alcohol-resistant foam, carbon-dioxide, or fog Spray to extinguish.
P403+P235 Store in a well ventilated place. Keep cool
P501 Dispose of contents/container in accordance with local regulations

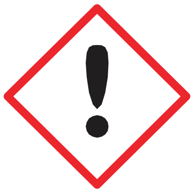

DANGER

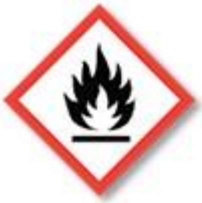

FLAMMABLE

SECTION 3: Composition/Information on Ingredients

Name	Product Identifier (CAS number)	GHS-US Classification
Ethanol SDA 40-B 200 Proof/SD Alcohol	64-17-5	
**PHYSICAL HAZARDS**	Flammable liquid	
**LABEL ELEMENTS**	**DANGER & FLAMMABLE LIQUID**	
**HAZARD SYMBOL**	**DANGER & FLAMMABLE LIQUID**	
**SIGNAL WORD**	**DANGER**	
**PREVENTION**	Observe good industrial hygiene practices.	

SECTION 4: First-Aid Measures

If unconscious, place in the recovery position and seek medical advice.

If the symptoms persist call a physician.

Avoid getting in the eyes. In case of contact with eyes, flush with water for 15 minutes. Protect unharmed eye.

Keep eye wide open while rinsing. Consult a physician for severe cases.

The product should not be an irritant; however, should the skin become irritated, wash with soap and water and discontinue use.

For external use only.

If ingested, contact a physician.

Do not induce vomiting. Never give anything by mouth to an unconscious person.

When symptoms are noticed, or in all cases of doubt, seek medical advice.

Keep out of reach of children.

SECTION 5: Fire Fighting Measures

5.1

Flammable Limits: N/A

Extinguishing Media: Dry chemical, water spray, alcohol-resistant foam, carbon Dioxide, or Fog Spray. Use extinguishing measures that are appropriate to local circumstances and the surrounding environment.

Hazard Combustible Products: Extremely flammable. Container may explode if heated. Closed containers are contained under pressure and may explode if exposed to excess heat for a prolonged period. Burning produces obnoxious and toxic fumes.

In the event of fire the following can be released: Carbon oxides; Acrolein; Sulphur oxides; nitrogen oxides (NOx); Other unidentified organic compounds.

Fire Fighting: Wear self-contained breathing apparatus and protective suit. Fight fire with standard precautions from a reasonable distance. Shield personnel to protect from venting or rupturing containers. Move containers from fire area if safe to do so. Cool closed containers exposed to fire with water spray. Do not allow run-off from fire fighting to enter drains or watercourse. Dike for water control.

SECTION 6: Accidental Release Measures

6.1

Personal Precautions: No action shall be taken involving any personal risk or without suitable training. Evacuate surrounding areas. Keep unnecessary and unprotected personnel from entering. Do not touch or walk through spilled material. Avoid breathing vapor mist. Provided adequate ventilation. Wear appropriate respirator when ventilation is inadequate. Put on personal protective equipment (See Section 8). Use non-slip safety shoes in the area where spills or leaks can occur.

Environmental Precautions: Avoid dispersal of spilled material and runoff and contact with soil, waterways, drains, and sewers. Inform the relevant authorities if the product has caused environmental pollution (sewers, waterways, soil or air).

Stop leak if without risk. Move containers from the spill area. Remove all sources of ignition. Ventilate the area. Prevent further leakage or spillage if safe to do so. Soak up with inert absorbent material (e.g. sand, silica, gel acid binder, universal binder, sawdust). Dispose of via a licensed waste disposal contractor. Move containers from spill area. Approach release from upwind.
Prevent entry into sewers, watercourses, basements or confined areas. Wash spillages into an effluent treatment plant or proceed as follows. Contain and collect spillages into an effluent treatment or proceed as follows. Contain and collect spillage with non-combustible, absorbent materials, sand, earth, vermiculite or diatomaceous earth and place in a container for disposal according to local regulations. Dispose of via a licensed waste disposal contractor. The contaminated absorbent material may pose the same hazard as the spilled product.

Note: See Section 1 for emergency contact information.

SECTION 7: Handling and Storage

7.1 Precautions for safe handling

Avoid contact with skin and eyes.

Non-Hazardous - Use standard nonhazardous handling and storage procedures. Put on appropriate personal protective equipment. (see **Section 8**). Eating drinking and smoking must be prohibited in areas where this material is handled, stored and processed. Workers should wash hands and face before eating, drinking and smoking. Remove contaminated clothing and protective equipment before entering eating areas. Do not ingest. Avoid contact with eyes, skin and clothing. Avoid breathing vapor or mist.

Use only in adequately ventilated place.

Wear appropriate respirator when ventilation is inadequate. Keep in the original container or an approved alternative made from a compatible material, kept tightly closed when not in use. Empty containers which retain product residue can be hazardous. Do not reuse container.

7.2 Conditions for safe storage, including any incompatibilities

Store in accordance with local regulations.

Keep out of reach of children.

Store in original container protected from direct sunlight.

Store in dry cool and well-ventilated area, away from incompatible materials, food and drink.

Keep container tightly closed and sealed until ready for use. Containers that have been opened must be carefully resealed and kept upright to prevent leakage.

Do not store in unlabeled containers. Use appropriate containment to avoid environmental contamination.

No smoking.

Observe label precautions.

Materials to Avoid: Strong Oxidizing Agents

SECTION 8: Exposure Controls/Personal Protection

8.1 Control parameters

Chemical Name / CAS No.	OSHA Exposure Limits	ACGIH Exposure Limits	Other Exposure Limits
Ethanol SDA 40-B 200 Proof/SD Alcohol 64-17-5	PEL 1000.000 ppm TWA VPEL 1000.000 ppm - TWA	TLV 1000.000 ppm - TWA	Not Established

8.2 Exposure controls

Wash hand, forearms and face thoroughly after handling chemical products, before eating, smoking and using the lavatory and at the end of the working period. Appropriate techniques should be used to remove potentially contaminated clothing. Wash contaminated clothing before reusing. Ensure that eyewash stations and safety showers are close to the workstation location.

Use only with adequate ventilation. If user operations generate dust, fumes, gas, vapor or mist, use process enclosures, local exhaust ventilation or other engineering controls to keep worker exposure to airborne contaminants below any recommended or statutory limits.

Environmental exposure controls: Emissions from ventilation or work process equipment should be checked to ensure they comply with the requirement of the environmental protection legislation. In some cases, fume scrubbers, filters or engineering modifications to the process equipment will be necessary to reduce emissions to acceptable levels.

Respiratory: Use a properly fitted, air-purifying or supplied-air respirator complying with an approved standard if a risk assessment indicated this is necessary. Respirator selection must be based on a known or anticipated exposure level, the hazards of the product and the safe working limits of the selected respirator.

Hands: Chemical-resistant, impervious gloves complying with an approved standard should be worn at all times when handling chemical products if a risk assessment indicated this is necessary.

Eyes: Safety eyewear complying with an approved standard should be used when a risk assessment indicates this is necessary to avoid exposure to liquid splashes, mist or dust. **Recommended:** Splash Goggles.

Skin: Personal protective equipment for the body should be selected based on the task being performed and the risks involved and should be approved by a specialist before handling this product.

SECTION 9: Physical and Chemical Properties

9.1 Information on basic physical and chemical properties

a) Appearance: Dark green gel

b) Odor: Characteristic.

**c) Odor Threshold:**	No information available.
**d) pH:**	No information available.
**e) Melting point/freezing point:**	No information available.
**f) Initial boiling point and boiling range:**	No information available.
**g) Flashpoint:**	<21°C
**h) Solubility:**	Dispersible in water
**i) Viscosity**	>1000 cps

SECTION 10: Stability and Reactivity

Stable under normal conditions **STABLE**

Components of this mixture are nonhazardous and non-reactive to other materials.

Under normal conditions of storage and use, hazardous reactions will not occur.

Should avoid heat, flames, and sparks.

Under normal conditions of storage and use, hazardous decomposition products should not be produced.

SECTION 11: Toxicological Information

11.1 Information on toxicological effects

Information on the product as supplied:

**Acute oral toxicity:**	LD50/oral/rat > 1,960 mg/kg (Estimated)
**Component Toxicity:**	Oral LD50/Human: 1,400 mg/kg
**Toxicology Information:**	No data found/ No component listed on (NTP), (IARC) or OSHA database
**Aspiration hazard:**	Due to the viscosity, this product does not present an aspiration hazard.

If contact does occur, This product may cause the following effects: May cause mild eye irritation. The heating of the product will cause the formation of mist and inhalation may cause irritation to the nose, throat and respiratory tract. The product causes green discoloration of the skin.

SECTION 12: Ecological Information

12.1 Toxicity

Information on the components of the product:

**Acute toxicity to fish:**	LC50/Oncorhynchus mykiss/96 hours = 14,200 mg/L. (Estimated)
**Acute toxicity to invertebrates:**	EC50/Daphnia magna/48 hours = 9,268 – 14,221 mg/L. (Estimated)
**Acute toxicity to algae:**	No data available.
**Chronic toxicity to fish:**	No data available.
**Chronic toxicity to invertebrates:**	No data available. Toxicity to microorganisms: No data available.
**Effects on terrestrial Organisms:**	No data available. Sediment toxicity: No data available.

SECTION 13: Disposal Considerations

13.1 Waste treatment methods

Like the US, EPA, state, regional, and other regulatory agencies may have jurisdiction over the disposal of your facility's hazardous waste, it is incumbent upon you, the hazardous waste generator to learn and satisfy the requirements that affect you.

SECTION 14: Transport Information

Agency	Proper Shipping Name	UN Number	Packing Group	Hazard Class
EU	ETHYL ALCOHOL SOLUTION	1170	II	3
IATA/ICA	ETHYL ALCOHOL SOLUTION	1170	II	3
IMDG	ETHYL ALCOHOL SOLUTION	1170	II	3
US DOT	ETHYL ALCOHOL SOLUTION	1170	II	3

SECTION 15: Regulatory Information

Safety, health and environmental regulations/legislation specific for the substance or mixture:

EU Risk Phrases

R11: Highly flammable
R22: Harmful if swallowed
R36: Irritating to eyes

Safety Phrase

S2: Keep out of the reach of children
S3: Keep in a cool place
S7: Keep container tightly closed
S15: Keep away from heat
S16: Keep away from sources of ignition - No smoking
S17: Keep away from combustible material
S20: When using do not eat or drink
S21: When using do not smoke
S25: Avoid contact with eyes
S35: This material and its container must be disposed of in a safe way

S29/35: Do not empty into drains; dispose of this material and its container in a safe way

TSCA 8(a) IUR Exempt/partial exemption: Not Determined

United States inventory (TSCA 8b): All components are listed or exempted

SARA 302/304/311/312 extremely hazardous substances: No products were found

SARA 302/304 emergency planning and notification: No products were found

SARA 302/304/311/312 hazardous chemicals: No products were found.

SARA 311/312 MSDS distribution- chemical inventory - hazard identification: No products were found.

SECTION 16: Regulatory Information

HMIS:		NFPA Ratings: NFPA:	
Health:	2	Health:	2
Flammability:	3	Flammability:	3
Physical Hazard:	1	Instability:	1
Personal Protection:	B	Special	0

Hazardous Material Information System (HMIS)

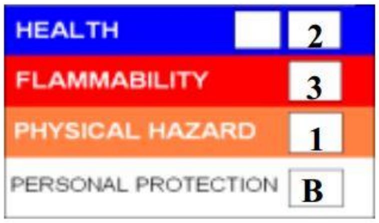

HMIS & NFPA Hazard Rating Legend
* = Chronic Health Hazard
0 = INSIGNIFICANT
1 = SLIGHT
2 = MODERATE
3 = HIGH

National Fire Protection Association (NFPA)

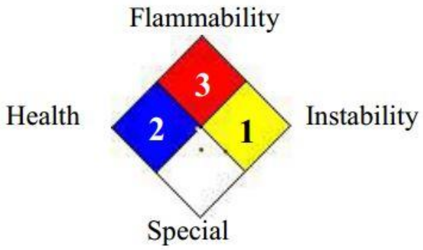

Preparation Date: 12 Nov 2019

SPECIAL NOTICE

The information in this SDS pertains only to the product as shipped. The information provided in this Safety Data Sheet is correct to the best of our knowledge, information and belief at the date of its publication. The information given is designed only as guidance for safe handling, use, processing, storage, transportation, disposal and release and is not to be considered a warranty or quality specification. The information relates only to the specific material designated and may not be valid for such material used in combination with any other materials or in any process, unless specified in the text.
