# Supplementary material for: Topical wound-care products and their effects on healing, inflammatory biomarkers, and growth in piglets undergoing castration
Source: Porcine Health Manag. 2026 Apr 21;12:23. doi: 10.1186/s40813-026-00492-7 (PMC13097753; doi:10.1186/s40813-026-00492-7)
Supplement: Supplementary file 4 — Supplementary Material 4 [file 40813_2026_492_MOESM4_ESM.pdf]

Stability: See below for date of expiration.

Disposal: No special disposal requirements. Vetericyn is environmentally friendly.

Ingredients: Active: Hypochlorous Acid (HOCl) (0.010%) **Inactive:** Electrolyzed Water (H20), Sodium Chloride (NaCl), Sodium Hypochlorite (NaOCl), Lithium Magnesium Sodium Silicate, Sodium Bicarbonate, Phosphates, Sodium Sulfate

Storage: Store at room temperature away from direct sunlight and heat. **DO NOT ALLOW TO FREEZE** because product may lose its potency. After use, seal or close dispenser.

Innovacyn products are manufactured under strict quality guidelines to ISO 9001, ISO 13485 and FDA QSR.

Made in the USA

Manufactured & Bottled by Innovacyn, Inc.
3546 N.Riverside Ave, Rialto CA 92377

vetericyn.com | 866.318.3116

Tell Us Your Story!

Lot Number:

Expiration Date:

- *Non-irritating & non-sensitizing*
- *Safe if licked or ingested*
- *Works on all animal skin types*

Indications: Vetericyn Wound and Skin Care is intended for the OTC management of skin abrasions, burns, lacerations, minor irritations, cuts and intact skin. Safe for use on all animal species.

Directions for use: Shake well before using. Move excess hair from wound area. Clip if necessary. Adjust spray nozzle. Saturate the affected area. If dressing is required, saturate dressing with Vetericyn HydroGel at each dressing change. Repeat 3-4 times per day until wound is healed. No rinsing necessary. HydroGel is designed to stick to the site of application and evaporate slowly, no need to wipe away film or residue. Faster healing is achieved when wounds are cleaned and maintained moist. Safe for use around mouth, nose, ears and eyes.

Use Precautions: For external use only. Discontinue use if irritation develops and contact your veterinarian. **For animal use only. Not for human use.** Keep out of reach of children. Safe if licked by animal. Not for injection. Always consult with a veterinarian for systemic conditions.

8 52009 00209 3

Part Number: 1040; 90007 v4.1

plus

Vetericyn®

advanced cleanser *plus* therapy

Hydro gel

Wound & Skin Care

Veterinarian Recommended

Topical application for wounds, cuts, burns, abrasions, sores & skin irritations

- *Safe & non-toxic*
- *No alcohol, steroids or antibiotics*

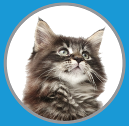
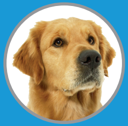
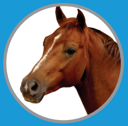
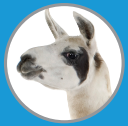

16 fl oz (473 ml)

SAFETY DATA SHEET
Vetericyn Plus® AX160G VF Antimicrobial Wound Barrier Gel Products
Part#AX65001

1. COMPANY IDENTIFICATION

Innovacyn Inc.
3546 N. Riverside Ave
Rialto, CA 92377
(866) 318-3116

Product Identification

Part Number	Size	Description
2045	8 oz. (237 mL)	Vetericyn Plus® VF Antimicrobial Wound Barrier Gel
2047	3 oz. (90 mL)	Vetericyn Plus® VF Antimicrobial Wound Barrier Gel

RECOMMENDED USE IDENTIFICATION

Vetericyn Plus® VF Antimicrobial Wound Barrier Gel is intended for use by veterinary professionals for moistening and debriding wounds of all types, burns, pressure sores, ulcers, abrasions, dermal and mucosal irritations, post-surgical incisions, grafted and donor sites, in addition to moistening and lubricating absorbent wound dressings. Vetericyn Plus® VF Antimicrobial Wound Barrier Gel relieves itch and pain often associated with dermal irritations. Promotes a moist environment which has been proven to help wounds heal faster.

RESTRICTIONS ON USE IDENTIFICATION

For external use only.

2. HAZARDS IDENTIFICATION

NOT HAZARDOUS to humans and animals.

Hazardous Material Identification System (HMIS) Rating:
Health: 0
Flammability: 0
Physical: 0; Reactivity: 0
Personal Protection Index: A

NFPA/HMIS Definitions:
0-Minimal Hazard
1-Slight Hazard
2-Moderate Hazard
3-Serious Hazard
4-Severe Hazard

The following values are obtained using the guidelines prepared by the National Fire Protection Association (NFPA) and American Coatings Association (HMIS)

HAZARDOUS AND/OR REGULATED COMPONENTS
None

HAZARDS DISCLOSURE
As defined by the OSHA Hazard Communication Standard 29 CFR 1910.1200 and defined under the Superfund Amendments & Reauthorization Act (SARA) 311 and 312, this product contains no known hazardous materials.

QA Original

3. COMPOSITION / INFORMATION ON INGREDIENTS

Ingredients	Percentage	CAS #
**Active:** Hypochlorous Acid	0.012%	7790-92-3
Electrolyzed Water	N/A	7732-18-5
Sodium Hypochlorite		7681-52-9
Sodium Chloride		7647-14-5
Lithium Magnesium Sodium Silicate		53320-86-8
Sodium Bicarbonate		144-55-8
Sodium Sulfate		7757-82-6
Phosphates		N/A

4. FIRST AID MEASURES

EYE CONTACT FIRST AID: Product is non-irritant, non-toxic. Flush with water, if wished.
SKIN CONTACT FIRST AID: Product is non-irritant, non-toxic. Flush with water, if wished.
INHALATION FIRST AID: Product is non-irritant, non-toxic. No known risks if inhaled.
INGESTION FIRST AID: Product is non-irritant, non-toxic.

5. FIRE-FIGHTING MEASURES

General Fire Hazards: None, Non-flammable
Hazardous Combustion Products: None, Non-flammable
Extinguishing Media: Product will not burn
Personal Protection: None required
Rate of Burning: Non-flammable

6. ACCIDENTAL RELEASE MEASURES

None required
Spill/Leak: Dike spill will inert absorbent materials to contain and soak up liquid. Place wastes into an appropriate waste disposal container. Product is non-hazardous, non-toxic.

7. HANDLING AND STORAGE

RECOMMENDED STORAGE TEMPERATURE: Ideal storage is at room temperature.
HANDLING: No special handling requirements.
STORAGE: Store at room temperature away from direct sunlight or heat.

8. EXPOSURE CONTROLS/PERSONAL PROTECTION

Permissible Exposure Limit (PEL): None
Threshold Limit Values (TLV): None
Engineering Controls: None
Personal Protective Equipment (PPE): None

9. PHYSICAL AND CHEMICAL PROPERTIES

Appearance: Liquid
Odor: Slightly chlorinated odor
Odor Threshold: Shelf life of product
PH: Balance between 6.0 - 7.5
Boiling Point: Same as water (212 °F / 99.98°C)
Melting Point: Not applicable
Flash Point: Not applicable
Evaporation Rate: Not applicable
Flammability (solid, gas): Non-flammable
Upper Flammable Limit (UFL): Non-flammable
Lower Flammable Limit (LFL): Non-flammable

FLAMMABLE LIMITS IN AIR
Lower Explosive Limit (LEL): None
Upper Explosive Limit (UEL): None

QA Original

Vapor Pressure: Not applicable
Vapor Density: Not applicable
Relative Density: 1.00-1.06
Solubility: Not applicable
Partition coefficient: Not applicable
Auto Ignition Temperature: Not applicable
Decomposition Temperature: Not applicable
Viscosity: 190-490 cPs

10. STABILITY AND REACTIVITY

Reactivity: Not applicable
Chemical Stability: Stable under recommended storage conditions
Possibility of hazardous reactions: Not hazardous
Conditions to avoid: Avoid freezing
Incompatible Materials: None
Hazardous decomposition products: None

11. TOXICOLOGICAL INFORMATION

EYE EFFECTS: No known hazards, non-toxic
SKIN EFFECTS: No known hazards, non-toxic
ORAL LD_{50}: >5000 mg/Kg. No known hazards, non-toxic
DERMAL LD_{50}: >5050 mg/Kg. No known hazards, non-toxic
INHALATION LC_{50}: >2.16 mg/l. No known hazards, non-toxic

Potential Health Affects

EYE: No potential health affects; product is non-hazardous.
SKIN: No potential health affects; product is non-hazardous.
INHALATION: No potential health affects; product is non-hazardous.
INGESTION: No potential health affects; product is non-hazardous.
CARCINOGENICITY INFORMATION: No known cancer hazards.
Product does not contain chemical which are listed in California's Safe Drinking Water & Toxic Enforcement Act of 1986, known as Proposition 65 (Prop 65).

QA Original

12. ECOLOGICAL INFORMATION

Presents no hazards to the environment
Ecotoxicity: Non-hazardous
Persistence and degradability: Unknown
Bioaccumulative potential: Biodegradable, non-hazardous
Mobility in soil: Unknown
Other adverse effects (such as hazardous to the ozone layer): Unknown

13. DISPOSAL CONSIDERATIONS

No special disposal considerations are required. Follow local ordinance for waste or recycling.

14. TRANSPORTATION INFORMATION

UN number: None required
UN proper shipping name: Oxidized water
Transport (D.O.T.) hazard class(es): Not DOT regulated
Freight Packing group, if applicable: 70, PG lll
Environmental hazards (e.g., Marine pollutant (Yes/No): NO
Transport in bulk (according to Annex II of MARPOL 73/78 and the IBC Code): Not applicable
Special precautions: None

15. Regulation

National Fire Protection Association (NFPA)
American Coatings Association (HMIS)
OSHA Hazard Communication Standard 29 CFR 1910.1200
Superfund Amendments & Reauthorization Act (SARA) 311 and 312
. NMFC-National-Motor-Freight-Classification
The US Department of Transportation
Annex II of MARPOL 73/78 and the IBC Code
APPENDIX D TO §1910.1200 - SAFETY DATA SHEETS

16. Other Information

SDS: Vetericyn Plus® AX160G VF Antimicrobial Wound Barrier Gel Products, Part #AX65001
Version: 1.0
Effective: February 25, 2020
DCO No: 071-19H

QA Original
