# Supplementary material for: Topical wound-care products and their effects on healing, inflammatory biomarkers, and growth in piglets undergoing castration
Source: Porcine Health Manag. 2026 Apr 21;12:23. doi: 10.1186/s40813-026-00492-7 (PMC13097753; doi:10.1186/s40813-026-00492-7)
Supplement: Supplementary file 5 — Supplementary Material 5 [file 40813_2026_492_MOESM5_ESM.pdf]

PhytoCare®

SWINE

SKIN RECOVERY AND CARE-SWINE

SWINE

An all-natural, spray-on solution that promotes healing and skin repair.

For use with: Lesions, Cuts, Abrasions, Punctures, Ulcers & Sores

PRECISION HEALTH TECHNOLOGIES

DIRECTIONS: Spray a light coating directly to cuts and abrasions. Apply twice daily until healing is complete (granulation, size reduction). For piglet processing: apply a light coating prior to and after processing.
FOR EXTERNAL SKIN APPLICATION ONLY.
FOR ANIMAL USE ONLY.

PRODUCT # 22011

8 60003 86494 4

ALL-NATURAL CONTENTS: All-Natural Contents: PHT proprietary formulation of PC-BE3 extract (grape blend, vitis vinifera), green tea (camellia sinensis) and pomegranate (punica granatum).

STORAGE TEMPERATURES between 40-80°F, do not allow product to freeze. **NET CONTENTS: 450ML**

Brookings, SD 57006 | 605-696-5606

Lot:

MATERIAL SAFETY DATA SHEET

NAME OF PRODUCT- PhytoCare® Skin Recovery and Care Natural Health Solution
Precision Health Technologies

SECTION 1: PRODUCT AND COMPANY IDENTIFICATION

PRODUCT NAME: PhytoCare® Skin Recovery and Care Natural Health Solution
PRODUCT CODES: ITM-220101

MANUFACTURER: Precision Health Technologies, LLC
ADDRESS: 120 22nd Ave S. #107
Brookings, SD 57006

EMERGENCY PHONE: 605-696-5606

CHEMICAL NAME: Proprietary meta-extraction of Punica granatum, Camellia Sinensis, Vitis vinifera
PRODUCT USE: Animal Health Solution Liquid
PREPARED BY: Product Development

SECTION 2: COMPOSITION/INFORMATION ON INGREDIENTS

INGREDIENTS: Purified Water, Punicus Granatus (Pomegranate) Extract, Camellia Sinensis (Green Tea) Extract, Vitis vinifera Extract

SECTION 3: HAZARDS IDENTIFICATION

EMERGENCY OVERVIEW: No known level of toxicity but may irritate sensitive mucosal tissues at high concentrations

ROUTES OF ENTRY: Oral, topical

POTENTIAL HEALTH EFFECTS

EYES: May cause burning sensation, redness

SKIN: No adverse effects expected on intact skin. Contact on burn or open wounds may cause minor stinging.

INGESTION: Large oral doses may cause minor irritation to the mouth or throat. May cause abdominal discomfort.

INHALATION: Not expected to be a health hazard under normal conditions.

ACUTE HEALTH HAZARDS: None known

CHRONIC HEALTH HAZARDS: None known

MEDICAL CONDITIONS GENERALLY AGGRAVATED BY EXPOSURE: None known

CARCINOGENICITY: None known

SECTION 4: FIRST AID MEASURES

EYES: Rinse immediately with water or saline

SKIN: Not expected to require first aid measures

INGESTION: Drink several glasses of water to dilute.

INHALATION: Not expected to require first aid measures

SECTION 5: FIRE-FIGHTING MEASURES

FLAMMABLE LIMITS IN AIR, UPPER: Not flammable; water based
(% BY VOLUME) LOWER: Not flammable; water based

FLASH POINT: Same as water

SPECIAL FIRE FIGHTING PROCEDURES: **N/A**

UNUSUAL FIRE AND EXPLOSION HAZARDS: **N/A**

HAZARDOUS DECOMPOSITION PRODUCTS: **N/A**

SECTION 6: ACCIDENTAL RELEASE MEASURES

ACCIDENTAL RELEASE MEASURES: Contain and recover liquid when possible. Collect liquid in an appropriate container or absorb with an inert material (e. g., vermiculite, dry sand, earth), and dispose of in a waste container. Moderate amounts may be flushed to the sewer with water.

SECTION 7: HANDLING AND STORAGE

HANDLING AND STORAGE: Store in a cool, well-ventilated dark area. Protect from freezing. Protect containers from physical damage.

OTHER PRECAUTIONS: **Seal tightly.**

SECTION 8: EXPOSURE CONTROLS/PERSONAL PROTECTION

SECTION 8 NOTES: **None required**

SECTION 9: PHYSICAL AND CHEMICAL PROPERTIES

APPEARANCE: **Slightly yellow brownish liquid.**

ODOR: **Slightly herbal.**

PHYSICAL STATE: **Liquid**

pH AS SUPPLIED: **3.0-5.0**

BOILING POINT: **212ºF, 100ºC**

FREEZING POINT: **32ºF, 0ºC**

SECTION 9: PHYSICAL AND CHEMICAL PROPERTIES (con't)

SOLUBILITY IN WATER: **100%**

PERCENT SOLIDS BY WEIGHT: **<1%**

SECTION 10: STABILITY AND REACTIVITY

STABILITY: **<u>Stable under ordinary conditions of use and storage</u>**

CONDITIONS TO AVOID (STABILITY): **Light, Heat, Freezing, Incompatibles**

INCOMPATIBILITY (MATERIAL TO AVOID): **Reducing agents, organic materials, rust, many metals**

HAZARDOUS DECOMPOSITION OR BY-PRODUCTS: **Decomposes to water and trace water soluble non-toxic plant material.**

HAZARDOUS POLYMERIZATION: **Will not occur**

SECTION 11: TOXICOLOGICAL INFORMATION

TOXICOLOGICAL INFORMATION: **No detectable limit found in animal studies**

SECTION 12: ECOLOGICAL INFORMATION

ECOLOGICAL INFORMATION: **Biodegradable. No known toxicity to animals**

SECTION 13: DISPOSAL CONSIDERATIONS

WASTE DISPOSAL METHOD: Dilute with water and flush to the sewer if local ordinances allow, otherwise, whatever cannot be saved for recovery or recycling should be managed in an appropriate and approved waste disposal facility. Processing, use or contamination of this product may change the waste management options. State and local disposal regulations may differ from federal disposal regulations. Dispose of container and unused contents in accordance with federal, state and local requirements.

SECTION 14: TRANSPORT INFORMATION

U.S. DEPARTMENT OF TRANSPORTATION

**PROPER SHIPPING NAME:**	**PhytoCare® Skin Recovery and Care**
**HAZARD CLASS:**	**N/A**
**ID NUMBER:**	
**PACKING GROUP:**	**Group III: least danger**
**LABEL STATEMENT:**	

WATER TRANSPORTATION

**PROPER SHIPPING NAME:**	**PhytoCare® Skin Recovery and Care**
**HAZARD CLASS:**	**N/A**
**ID NUMBER:**	
**PACKING GROUP:**	**Group III: least danger**
**LABEL STATEMENTS:**	

AIR TRANSPORTATION

**PROPER SHIPPING NAME:**	**PhytoCare® Skin Recovery and Care**
**HAZARD CLASS:**	**N/A**
**ID NUMBER:**	
**PACKING GROUP:**	**Group III: least danger**
**LABEL STATEMENTS:**	

OTHER AGENCIES:

SECTION 15: REGULATORY INFORMATION

U.S. FEDERAL REGULATIONS

TSCA (TOXIC SUBSTANCE CONTROL ACT):

CERCLA (COMPREHENSIVE RESPONSE COMPENSATION, AND LIABILITY ACT): **N/A**

SARA TITLE III (SUPERFUND AMENDMENTS AND REAUTHORIZATION ACT): **N/A**

311/312 HAZARD CATEGORIES: **N/A**

313 REPORTABLE INGREDIENTS: **N/A**

STATE REGULATIONS: **Right to know- Chemical name above**

INTERNATIONAL REGULATIONS:

SECTION 16: OTHER INFORMATION

OTHER INFORMATION

PREPARATION INFORMATION: **Dilute for use per label instructions.**

Disclaimer:
****
Precision Health Technologies provides the information contained herein in good faith but makes no representation as to its comprehensiveness or accuracy. This document is intended only as a guide to the appropriate precautionary handling of the material by a properly trained person using this product. Individuals receiving the information must exercise their independent judgment in determining its appropriateness for a particular purpose. Precision Health Technologies MAKES NO REPRESENTATIONS OR WARRANTIES, EITHER EXPRESS OR IMPLIED, INCLUDING WITHOUT LIMITATION ANY WARRANTIES OF MERCHANTABILITY, FITNESS FOR A PARTICULAR PURPOSE WITH RESPECT TO THE INFORMATION SET FORTH HEREIN OR THE PRODUCT TO WHICH THE INFORMATION REFERS. ACCORDINGLY, PRECISION HEALTH TECHNOLOGIES LLC. WILL NOT BE RESPONSIBLE FOR DAMAGES RESULTING FROM USE OF OR RELIANCE UPON THIS INFORMATION.
****

PhytoCare™

SKIN RECOVERY AND CARE

DIRECTIONS

Spray a light coating directly to cuts and abrasions. Apply twice daily until healing is complete (granulation, size reduction). **For piglet processing:** apply a light coating prior to and after processing.

Storage Temperatures between 40-80°F, do not allow product to freeze.

> *This natural technology fills an overlooked niche in improving skin wound healing in pigs.*
>
> **- Dr. Andrew Bents, DVM**

HOW IT WORKS

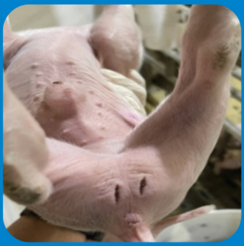

Lidocaine Treatment

VS

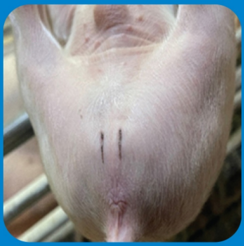

PhytoCare Skin Recovery & Care

RESULTS IN 24 HOURS

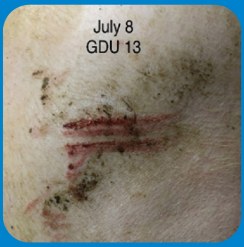

Pre-Treatment

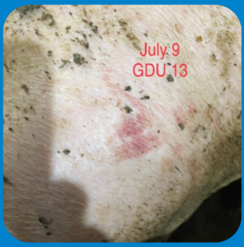

24 hrs Post-Treatment

PhytoCare™ Skin Recovery and Care is an advanced formulation of activated plant polyphenols. Polyphenols use the natural properties of plants to provide rapid healing. For use with: **Lesions, Cuts, Abrasions, Punctures, Ulcers & Sores**

QUICKER CLOSURE

FASTER HEALING

LESS SWELLING

SKIN PROTECTION

EASY APPLICATION

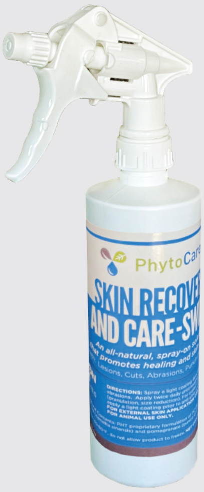

For Animal Use Only

Patent Protected

PRECISION HEALTH TECHNOLOGIES

Brookings, SD USA

**All-Natural Product, no Therapeutic Claims made.*

(605) 696-5606 | customerservice@precisionhealthtech.com | PRODUCT #22011
