# Supplementary material for: Topical wound-care products and their effects on healing, inflammatory biomarkers, and growth in piglets undergoing castration
Source: Porcine Health Manag. 2026 Apr 21;12:23. doi: 10.1186/s40813-026-00492-7 (PMC13097753; doi:10.1186/s40813-026-00492-7)
Supplement: Supplementary file 6 — Supplementary Material 6 [file 40813_2026_492_MOESM6_ESM.docx]

# SUPPLEMENTARY MATERIAL

| **Table S1.** Least-squares means (± SEM) of prostaglandin E₂ (PGE₂, pg/mL) concentrations in male piglets by treatment and timepoint. | | | |
| --- | --- | --- | --- |
| **Treatment** | **Timepoint (day)** | | |
|  | **Day 0** | **Day 7** | **Day 14** |
| Iodine | 133.8 ± 11.8ᵇ | 77.2 ± 7.0ᵃ | 87.5 ± 7.9ᵃ |
| NoCast | 137.8 ± 14.3ᵇ | 78.0 ± 8.0ᵃ | 96.4 ± 10.1ᵃ |
| Oinkment | 135.5 ± 11.9ᶜ | 96.7 ± 8.8ᵇ | 74.7 ± 6.8ᵃ |
| PhytoCare | 135.8 ± 12.1ᵇ | 77.3 ± 7.2ᵃ | 83.3 ± 8.0ᵃ |
| Vetericyn | 143.9 ± 12.9ᵇ | 71.2 ± 6.4ᵃ | 81.2 ± 7.3ᵃ |
| ZincOxide | 126.5 ± 11.2ᵇ | 78.2 ± 7.1ᵃ | 65.5 ± 5.9ᵃ |
| Values are estimated marginal means back transformed from the log scale. Superscript letters (a–c) indicate statistical groupings based on Holm-adjusted *P*-values (α = 0.05) for comparisons among timepoints within each treatment. Means sharing the same letter are not significantly different. | | | |

| **Table S2.** Least-squares means (± SEM) of plasma haptoglobin concentrations (mg/dL) in male piglets by treatment and timepoint. | | | |
| --- | --- | --- | --- |
| **Treatment** | **Timepoint (day)** | | |
|  | **Day 0** | **Day 7** | **Day 14** |
| Iodine | 30.2 ± 6.0ᵃ | 87.6 ± 18.2ᵇ | 122.8 ± 25.9ᵇ |
| NoCast | 27.5 ± 6.1ᵃ | 57.7 ± 12.5ᵇ | 65.0 ± 14.8ᵇ |
| Oinkment | 29.2 ± 5.8ᵃ | 153.8 ± 31.7ᶜ | 87.5 ± 18.2ᵇ |
| PhytoCare | 29.4 ± 5.9ᵃ | 118.0 ± 24.7ᵇ | 95.1 ± 19.8ᵇ |
| Vetericyn | 30.4 ± 6.2ᵃ | 63.6 ± 12.8ᵇ | 86.6 ± 17.7ᵇ |
| ZincOxide | 27.9 ± 5.6ᵃ | 79.1 ± 16.2ᵇ | 82.6 ± 16.8ᵇ |
| Values are estimated marginal means back transformed from the log scale. Superscript letters (a–c) denote statistical groupings based on Holm-adjusted *P*-values (α = 0.05) for comparisons among timepoints within each treatment. Means sharing the same letter are not significantly different. | | | |

**ADDITIONAL FILES**

1. **Additional file 1**

**File format:** Portable Document Format; PDF

**Title of data:** Iodine Wound Spray Label and Safety Data Sheet

**Description of data:** Customer proof label artwork for “Iodine Wound Spray” topical antiseptic product.

1. **Additional file 2**

**File format:** Portable Document Format; PDF

**Title:** Oikment Label and Safety Data Sheet

**Description of data:** Customer proof label artwork for “Oikment” topical antiseptic product.

1. **Additional file 3**

**File format:** Portable Document Format; PDF

**Title:** PhytoCare Skin Recovery and Care - Swine Label and Safety Data Sheet

**Description of data:** Customer proof label artwork for “PhytoCare Skin Recovery and Care - Swine” topical antiseptic product.

1. **Additional file 4**

**File format:** Portable Document Format; PDF

**Title:** Vetericyn Plus Label and Safety Data Sheet

**Description of data:** Customer proof label artwork for “Vetericyn Plus” topical antiseptic product.

1. **Additional file 5**

**File format:** Portable Document Format; PDF

**Title:** Zinc Oxide Ointment USP Label and Safety Data Sheet

**Description of data:** Customer proof label artwork for “Zinc Oxide Ointment USP” topical antiseptic product.
